# Sexual Dimorphisms of Protein-Coding Gene Profiles in Placentas From Women With Systemic Lupus Erythematosus

**DOI:** 10.3389/fmed.2022.798907

**Published:** 2022-03-15

**Authors:** Hui-hui Li, Lin-tao Sai, Shan Tian, Yuan Liu, Colman I. Freel, Kai Wang, Chi Zhou, Jing Zheng, Qiang Shu, Ying-jie Zhao

**Affiliations:** ^1^Department of Obstetrics and Gynecology, Qilu Hospital, Cheeloo College of Medicine, Shandong University, Jinan, China; ^2^Department of Obstetrics and Gynecology, University of Wisconsin-Madison, Madison, WI, United States; ^3^Department of Infectious Diseases, Qilu Hospital, Cheeloo College of Medicine, Shandong University, Jinan, China; ^4^Center for Reproductive Medicine, Jinan Central Hospital, Cheeloo College of Medicine, Shandong University, Jinan, China; ^5^Clinical and Translational Research Center, Shanghai First Maternity and Infant Hospital, Tongji University School of Medicine, Shanghai, China; ^6^School of Animal and Comparative Biomedical Sciences, University of Arizona, Tucson, AZ, United States; ^7^Department of Rheumatology, Qilu Hospital, Cheeloo College of Medicine, Shandong University, Jinan, China; ^8^Shandong Provincial Clinical Research Center for Immune Diseases and Gout, Jinan, China

**Keywords:** systemic lupus erythematosus, protein-coding RNA, placenta, pregnancy, fetal sex

## Abstract

**Background:**

Systemic lupus erythematosus (SLE) may cause pathogenic changes in the placentas during human pregnancy, such as decreased placental weight, intraplacental hematoma, ischemic hypoxic change, placental infarction, and decidual vasculopathy, which contribute to high maternal and fetal mortality and morbidity. Sex-specific adaptations of the fetus are associated with SLE pregnancies. The present study aimed to determine the transcriptomic profiles of female and male placentas from women with SLE.

**Methods:**

RNA sequencing (RNA-seq) was performed to identify differentially expressed protein-coding genes (DEGs) in placentas from women with SLE vs. normal term (NT) pregnancies with female and male fetuses (*n* = 3-5/sex/group). Real-time-quantitative PCR was performed (*n* = 4 /sex/group) to validate the RNA-seq results. Bioinformatics functional analysis was performed to predict the biological functions and pathways of SLE-dysregulated protein-coding genes.

**Results:**

Compared with NT-female (NT-F) placentas, 119 DEGs were identified in SLE-female (SLE-F) placentas. Among these 119 DEGs, five and zero are located on X- and Y-chromosomes, respectively, and four are located on the mitochondrial genome. Compared with NT-male (NT-M) placentas, 458 DEGs were identified in SLE-male (SLE-M) placentas, among which 16 are located on the X-chromosome and zero on the Y-chromosome and mitochondrial genome. Twenty-four DEGs were commonly dysregulated in SLE-F and -M placentas. Functional analysis showed that SLE-dysregulated protein-coding genes were associated with diverse biological functions and pathways, including angiogenesis, cellular response to growth factor stimulus, heparin-binding, HIF (hypoxia-inducible factor)-1 signaling pathway, and Interleukin-17 (IL-17) signaling pathway in both SLE-F and -M placentas. Biological regulations were differentially enriched between SLE-F and -M placentas. Regulation of blood circulation, response to glucocorticoid, and rhythmic process were all enriched in SLE-F, but not SLE-M placentas. In contrast, tumor necrosis factor production, Th17 cell differentiation, and MDA (melanoma differentiation-associated gene)-5 signaling pathway were enriched in SLE-M but not SLE-F placentas.

**Conclusion:**

This report investigated the protein-coding gene profiles of placenta tissues from SLE patients using RNA-seq. The results suggest that the SLE-dysregulated protein-coding genes in placentas may contribute to the pathophysiological progress of SLE pregnancies in a fetal sex-specific manner, leading to adverse pregnancy outcomes.

## Introduction

Systemic lupus erythematosus (SLE) is a chronic autoimmune disease that predominantly affects women of reproductive age, typically causing damages to multiple organ systems (1, 2). SLE pregnancies are associated with maternal complications (e.g., lupus flare, hypertension, preeclampsia, and eclampsia) and fetal complications (e.g., stillbirth, spontaneous abortion, fetal growth restriction, neonatal lupus, and neonatal deaths), and increase the induced abortion rate (3–5).

SLE pregnancies are associated with many placental dysfunctions, (e.g., decreased placental weight, intraplacental hematoma, chronic villitis, thickening of the trophoblast basement membrane, ischemic hypoxic change, placental infarction, decidual vasculopathy, and fetal thrombi) (3, 6). Many mechanisms underlying SLE-induced placental and fetal dysfunction have been proposed. The fetal-placental immune system could in turn, interact with the maternal immune system and mediate maternal immune response (7). For example, anti-DNA antibodies in SLE pregnancies may inhibit trophoblast attachment and migration via cross-reacting with laminin (8). Antiphospholipid antibodies may also alter the placental phospholipid membrane and cause infarctions and edema/swelling (9, 10). In addition, anti-SSA (Sjogren's-syndrome-related antigen A) and anti-SSB (Sjogren's-syndrome-related antigen B) antibodies can cause neonatal lupus and induce fetal injury after crossing the placenta (11). Thus, defects in the placental and fetal responses to autoimmune processes and inflammation are closely related to SLE pregnancy.

Sex-specific adaptations of the fetus have been reported in many complicated pregnancies such as SLE, asthma, and preeclampsia (12–14). Specifically, a lower proportion of male fetuses were born to women with SLE, which may be partially attributed to different chronic inflammation responses between male and female fetuses in early gestation (13). During asthma pregnancy, significantly reduced birth weights were observed in women with female, but not male fetuses (12), suggesting that the inflammatory activities of asthma impacted female, but not male fetuses. Recently, Zhou et al. demonstrated that preeclampsia impaired fetal endothelial function in a fetal sex-dependent manner (14). Given that the placenta is a key organ that closely regulates fetal growth and function, a sex-specific dysregulation of placental growth and function might contribute to the adverse pregnancy outcomes induced by SLE.

Different mechanisms may govern the fetal sex-specific adaptations in various complicated pregnancies. For example, Murphy et al. (12) have reported that female but not male fetuses born to mothers with asthma are associated with a significant increase of maternal circulating monocytes and decreases in placental steroid hydroxylase activity and fetal estriol. Zhou et al. (14) have also reported that fetal-sex specific expression of genes accompanied by preeclampsia-impaired fetal endothelial function. However, mechanisms controlling sex-specific adaptations of SLE pregnancies remain unknown.

In this study, we hypothesized that SLE alters gene expression of placentas, disturbing placental biological functions in a sex-specific manner. We determined the expression profiles of protein-coding genes of placentas from SLE and normal term (NT) pregnancies using RNA sequencing (RNA-seq). Real-time quantitative PCR (RT-qPCR) was conducted to verify RNA-seq results. Functional analysis was conducted to describe the underlying biological functions of differentially expressed protein-coding genes (DEGs) in female and male placentas from SLE pregnancies.

## Materials and Methods

### Ethical Approval

All procedures were conducted in accordance with the Declaration of Helsinki. Two sets of placental tissues were collected from two hospitals. The first set (SLE, *n* = 10 with five female and five male fetuses) was collected in Qilu Hospital, Shandong University. The tissue collection protocol was approved and carried out in accordance with the regulation of the Institutional Review Board of Qilu Hospital, Shandong University. SLE was defined according to the American College of Rheumatology classification criteria (15). The SLE disease activity index (SLEDAI) (16) was used to assess the disease activity of SLE patients. SLEDAI scores were assessed within 1 week before delivery. The second set (NT, *n* = 10 with five female and five male fetuses) was collected in Shanghai First Maternity and Infant Hospital affiliated with Tongji University. The tissue collection protocol was approved and carried out in accordance with the regulation of the Ethical Committee of Shanghai First Maternity and Infant Hospital affiliated with Tongji University. All individuals included in this study were Han Chinese without information on their ancestries. Smokers and patients with cancer or diabetes mellitus were excluded.

### RNA Isolation and Quality Control

Placental tissues were obtained within 30 min after vaginal delivery or cesarean section delivery. Placental villi were dissected beneath the chorionic and basal plates (~1 × 1 cm). Placental tissues were snap-frozen in liquid nitrogen and stored at −80°C. Total RNA was isolated from placental tissues using the RNeasy mini kit (Qiagen, Germany). The concentration and quality of RNA samples were assessed using a NanoDrop One spectrophotometer (Thermo Fisher Scientific, USA) and Agilent 2100 Bioanalyzer (Agilent Technologies, USA). Samples with RNA integrity number (RIN) values ≥ 7.0 were used for sequencing.

### RNA-Seq and Bioinformatics Analysis of Data

We performed RNA-seq analysis of total RNA samples from placentas (*n* = 3-5/sex/group; Supplementary Table 1) as described in supplementary methods. RNA-seq strand-specific libraries were constructed using the VAHTS Total RNA-seq (H/M/R) Library Prep Kit (Vazyme, China) following the manufacturer's instructions. Purified libraries were quantified and validated by Qubit 2.0 Fluorometer and Agilent 2100 bioanalyzer to confirm the insert size and calculate the mole concentration. The library construction and sequencing were performed by Sinotech Genomics Co., Ltd Shanghai, China). Overall, more than 66 million reads per sample were generated. Reads with non-canonical letters or low quality and sequences shorter than 25 nucleotides were removed. Reads were trimmed off using FASTQ software (17). Trimmed reads were mapped to the GRCH38 genome using the HISAT2 software (18). DEGs were identified and analyzed using Cuffdiff (19) and R package edgeR (20), respectively. The fold change (FC) was estimated according to the Fragments Per Kilobase of transcript sequence per Millions base pairs sequenced (FPKM) values (21, 22). The *P-*value significance threshold was set according to the Benjamini-Hochberg false discovery rate (FDR). DEGs were selected using the following criteria: FC > |2| and FDR adjusted *P*-value (*q*-value) <0.05 (14), were considered as significantly modulated and recognized as SLE-dysregulated genes. The RNA-seq data have been uploaded and deposited in NCBI (National Center for Biotechnology Information) Gene Expression Omnibus (GEO) (https://www.ncbi.nlm.nih.gov/geo/, accession number: GSE177029).

### Functional Analysis of SLE-Dysregulated Genes

The Metascape online analysis tool (https://metascape.org/) was used to predict the biological functions and signaling pathways of the SLE-dysregulated genes (23). Biological terms were selected using the following criteria: *P*-value <0.01, enrichment factor >1.5, and term count > 3. The most statistically significant (lowest *p*-value) biological terms within each cluster were chosen to represent the cluster in bar graphs. A subset of representative terms from the cluster was selected and converted as a network plot. Terms with a similarity score (23) > 0.3 were connected by an edge, and the thickness of the edge represents the similarity score. Cytoscape (v3.1.2) was used to visualize the network (24).

### RT-PCR

Total RNA (400 ng/sample) was transcribed into complementary DNA (cDNA) using the HiScript II Q RT SuperMix for qPCR (Vazyme Biotech, cat#: R222-01). Diluted cDNA corresponding to 4 ng of original total RNA was utilized as the template in each RT-qPCR reaction. To validate the RNA-seq results, 10 candidate genes were selected for RT-qPCR analysis (25) (*n* = 4 /sex/group; Supplementary Table 1) using NuHi Robustic SYBR Green Mix. We chose these candidate genes based on the fold changes in SLE vs. NT, relevance to placental function (i.e., angiogenesis and immune responses), expression abundances, and different expression patterns in SLE-F and -M placentas according to RNA-seq data. Primers are listed in Supplementary Table 2. Data were normalized to *GAPDH* and then analyzed using the 2^−Δ*ΔCT*^ method (25, 26).

### Correlation Analysis of SLE-Dysregulated Genes and SLEDAI Scores

After RT-qPCR verification, relative expression levels of SLE-dysregulated genes were used to analyze the correlation with SLEDAI scores.

### Statistical Analyses

Microsoft Excel (2016) for Windows and SigmaPlot (13.0) for Windows were used for statistical analyses. Data were represented as the medians ± standard deviation (SD) or medians with range. Data were analyzed using student's *t*-test or Mann-Whitney Rank Sum Test when applicable. The relationship between the relative expression levels of genes and SLEDAI scores was analyzed by Pearson's correlation coefficient. *P*-values <0.05 were considered statistically significant.

## Results

### Patient Characteristics

All SLE patients received maintenance corticosteroids (prednisone ≤ 15 mg daily) and hydroxychloroquine ( ≤ 400 mg daily) during pregnancy. Three patients in the SLE group were antiphospholipid positive and were on aspirin (100 mg daily) during pregnancy. One SLE patient was positive for anti-SSA antibody. None of the newborns developed neonatal lupus. Patient ages and body mass index (BMI) were similar between NT and SLE pregnancies. However, the newborn body weight for SLE-F was significantly lower than that for NT-F (*P* = 0.026) (Table 1 and Supplementary Table 1). There was one sample from the SLE group with a growth-restricted male fetus (27). Two patients each in SLE and NT group underwent scheduled Cesarean section delivery. Demographic and clinical characteristics are shown in Table 1 and Supplementary Table 1.

**Table 1:** Clinical characteristics.

**Characteristics**	**SLE (*n* = 10)**	**NT (*n* = 10)**	***P***
Age (years), median (range)	29.0 (26–36)	30.5 (28–33)	0.667
BMI, median (range)	25.4 (23.0-32.3)	28.0 (21.8-32.7)	0.663
Gestation age (weeks), median (range)	38.5 (34.9-39.7)	39.1 (38.6-40.1)	0.053
Fetal weight (grams), median (range)	2950.0 (2150.0-3850.0)	3402.0 (2895.0-3730.0)	0.026
Disease duration (months), median (range)	40.0 (10–167)	-	-
SLEDAI score, median (range)	2.5 (0-6)	-	-
ANA > 1:320, yes/no (*n*)	10/0	-	-
Anti-dsDNA, yes/no (*n*)	2/8	-	-
Anti-SSA/SSB	1/9	-	-
Anti-phospholipid, yes/no (*n*)	3/7	-	-
Preeclampsia, yes/no (*n*)	0/10	-	-
Proteinuria, yes/no (*n*)	3/7	-	-
Hypocomplementemia, yes/no (*n*)	3/7	-	-

*SLE, systemic lupus erythematosus; NT, normal term; BMI, body mass index; SLEDAI, systemic lupus erythematosus disease activity index; ANA, antinuclear antibody; SSA, Sjogren's-syndrome-related antigen A; SSB, Sjogren's-syndrome-related antigen B*.

### Distinct Transcriptional Profile in Placentas From SLE Pregnancies

**Figure 1:**
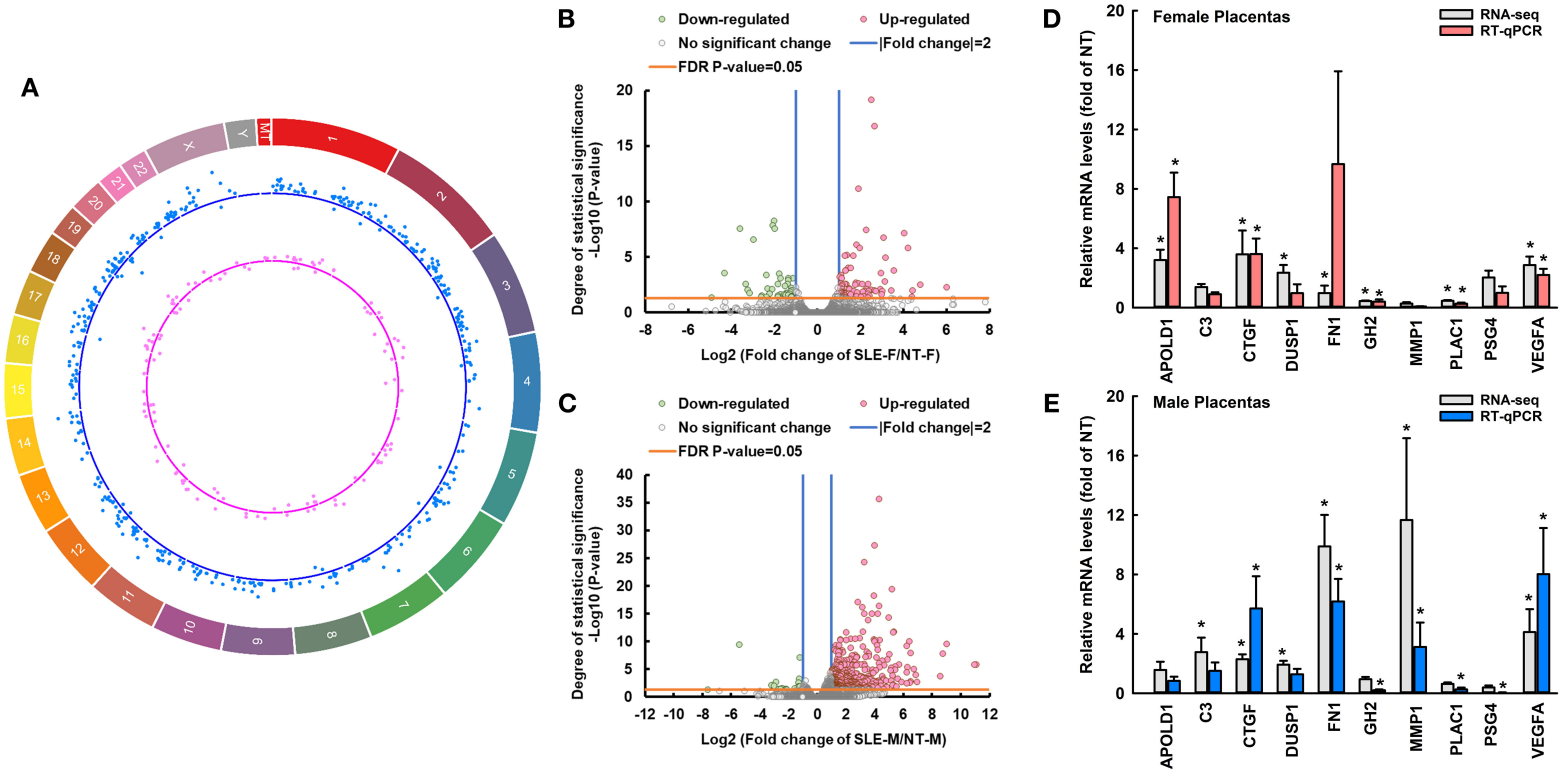
SLE differentially dysregulates transcriptomic profiles of female and male placentas. **(A)** Circos plot illustrating the chromosome location of differentially expressed protein-coding genes between SLE-F vs. NT-F (pink dots) and SLE-M vs. NT-M (blue dots). Each dot represents one differentially expressed gene. The letters and numbers in the outer layer represent the chromosome location. For the scatter plot tracks, dots outside and inside of the centerline represent upregulated and downregulated genes, respectively. **(B,C)** Volcano plots showing differentially expressed genes between SLE-F vs. NT-F, and SLE-M vs. NT-M in RNA-seq. Gray dots: no significant difference; pink and green dots: > two-fold upregulation and downregulation, respectively (FDR-adjusted *P*-value < 0.05) in SLE vs. NT; *n* = 3-5/group. **(D,E)** RT-qPCR validation of SLE-dysregulated genes in female and male placentas. ^*^P < 0.05 vs. NT, *n* = 4/group. MT, mitochondrial DNA; SLE, systemic lupus erythematosus; APOLD1, apolipoprotein L domain containing 1; C3, complement C3; CTGF, connective tissue growth factor; DUSP1, dual specificity phosphatase 1; GH2, growth hormone 2; PLAC1, placenta specific 1; FN1, fibronectin 1; PSG4, pregnancy-specific beta-1-glycoprotein 4; VEGFA, vascular endothelial growth factor A; MMP1, matrix metallopeptidase 1. RNA-seq, RNA sequencing; FDR, false discovery rate. Circos plot showing the location DE-genes was generated using circa software for Windows.

Compared with NT, SLE dysregulated 119 protein-coding genes in female placentas (Figure 1A; Supplementary Table 3), among which five and zero are located on the X- and Y-chromosomes, respectively, and four are located on the mitochondrial genome. Compared with NT, SLE dysregulated 458 protein-coding genes in male placentas (Figure 1A; Supplementary Table 3), among which 16 are located on the X-chromosomes and zero are located on the Y-chromosome or mitochondrial genome (Figure 1A; Supplementary Tables 3, 4).

Compared to NT-F, 77 and 42 genes were upregulated and downregulated in SLE-F placentas, respectively (Figure 1B; Supplementary Table 3). Compared to NT-M, 438 and 20 genes were upregulated and downregulated in SLE-M placentas, respectively (Figure 1C; Supplementary Table 3). Twenty-four genes were commonly dysregulated in SLE-F and -M placentas (Supplementary Table 3), among which 21were commonly upregulated in SLE-F and -M placentas. One (*MATR3*) was upregulated in SLE-F placentas but downregulated in SLE-M placentas, while two (*CP* and *LGALS3BP*) were upregulated in SLE-M placentas but downregulated in SLE-F placentas (Supplementary Table 3). None of these commonly dysregulated genes is on the X- chromosome, Y-chromosome or mitochondrial genome.

The correlation RT-qPCR and RNA-seq results was performed using the fold change (SLE/NT) of 10 selected genes in RT-qPCR and RNA-seq analyses. RT-qPCR data were correlated significantly with RNA-seq analysis (*r* = 0.637, *P* < 0.05) (Figures 1D,E). Specifically, *CTGF, VEGFA*, and *GH2* were upregulated in SLE-F and SLE-M placentas. *MMP1* was downregulated in SLE-F but upregulated in SLE-M placentas. *APOLD1* was upregulated in SLE-F but not in SLE-M placentas. *FN1, PLAC1*, and *PSG4* were upregulated in SLE-M but not in SLE-F placentas. SLE did not alter *C3* and *DUSP1* expression in female and male placentas.

### Functional and Pathway Analyses of SLE-Dysregulated Genes in Placentas

**Figure 2:**
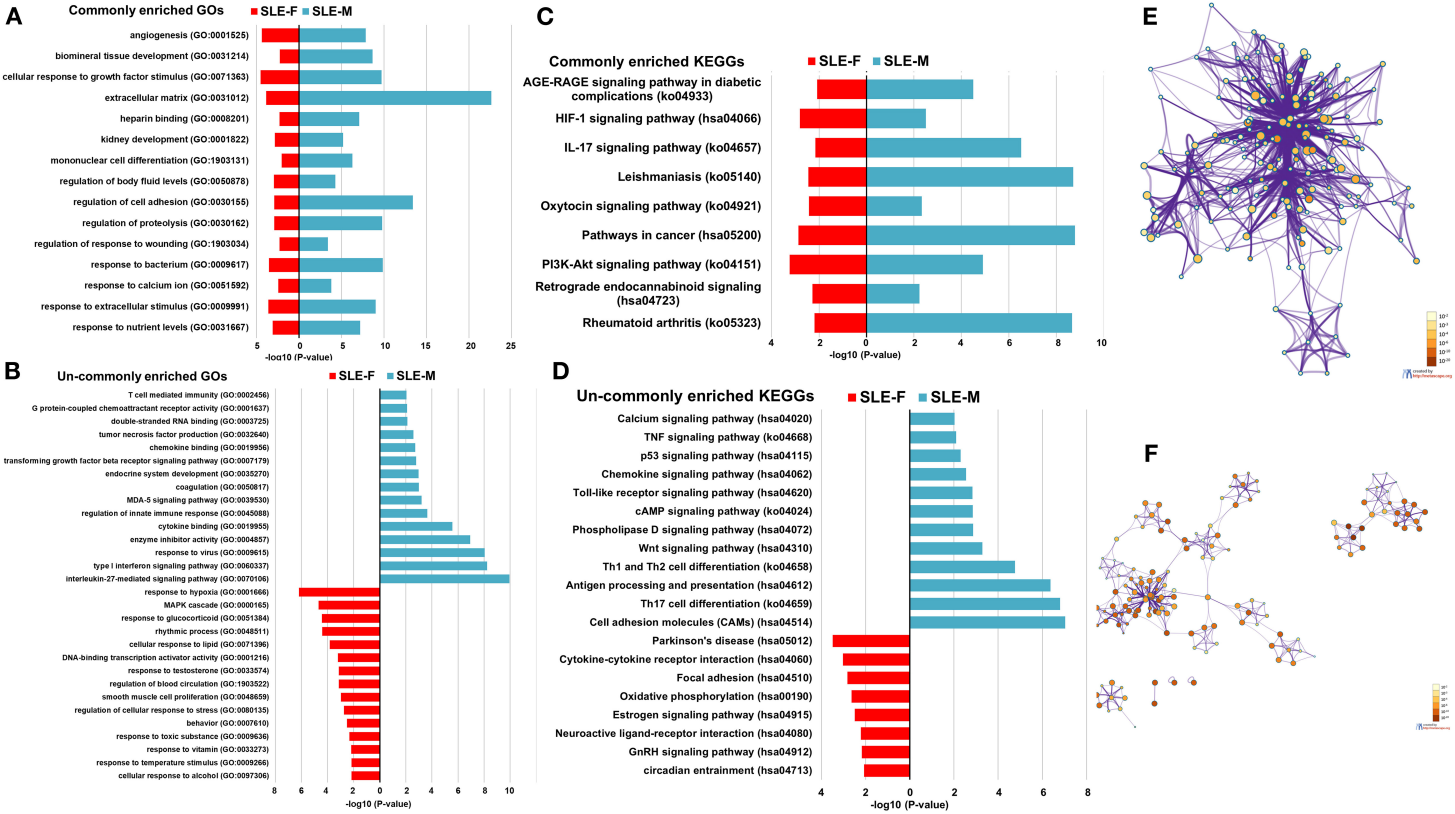
Enrichment analysis of dysregulated protein-coding genes in SLE-F and -M placentas. **(A)** Commonly enriched GOs terms from both SLE-F and -M placentas. **(B)** Un-commonly enriched GO terms from SLE-F and -M placentas, respectively. **(C)** Commonly enriched KEGG terms from both SLE-F and -M placentas. **(D)** Un-commonly enriched KEGG terms from SLE-F and -M placentas, respectively. **(E)** Network of GO and KEGG enriched terms from SLE-F placentas colored by *P*-value, where terms containing more dysregulated genes indicate a more significant *P*-value. **(F)** Network of GO and KEGG enriched terms from SLE-M placentas colored by *P*-value, where terms containing more genes tend to have a more significant *P*-value. SLE, systemic lupus erythematosus; F, female; M, male; GO, Gene Ontology; KEGG, Kyoto Encyclopedia of Genes and Genomes.

SLE-dysregulated protein-coding genes were associated with diverse biological functions and pathways (284 for SLE-F placentas and 422 for SLE-M placentas) (Supplementary Tables 5, 6). Sixty-two biological functions and pathways were commonly enriched in both SLE-F and SLE-M placentas (Supplementary Tables 5, 6). These biological functions and pathways included angiogenesis, cellular response to growth factor stimulus, heparin-binding, kidney development, mononuclear cell differentiation, pathways in cancer, response to calcium ion, HIF (hypoxia-inducible factor)-1 signaling pathway, IL-17 signaling pathway, and PI3K-Akt signaling pathway (Figures 2A,C; Table 2, Supplementary Tables 5, 6).

**Table 2:** SLE dysregulated biological functions in female and male placentas.

**Biological functions**	**Female placentas**		**Male placentas**	
	***P*-value**	**DEG number in SLE vs. NT**	***P*-value**	**DEG number in SLE vs. NT**
Angiogenesis	4.24 × 10^−5^	11	1.44 × 10^−8^	31
Cellular response to growth factor stimulus	3.08 × 10^−5^	12	1.89 × 10^−10^	37
Heparin binding	5.15 × 10^−3^	4	8.41 × 10^−8^	15
HIF-1 signaling pathway	1.56 × 10^−3^	4	3.14 × 10^−3^	7
IL-17 signaling pathway	6.91 × 10^−3^	6	3.02 × 10^−7^	11
Regulation of blood circulation	6.64 × 10^−4^	3		
Response to glucocorticoid	3.69 × 10^−5^	6		
Rhythmic process	3.86 × 10^−5^	8		
GnRH signaling pathway	6.71 × 10^−3^	4		
Estrogen signaling pathway	3.19 × 10^−3^	3		
Tumor necrosis factor production			2.62 × 10^−3^	9
Th17 cell differentiation			1.58 × 10^−7^	12
IL-27-mediated signaling pathway			1.14 × 10^−10^	6
MDA-5 signaling pathway			6.16 × 10^−4^	3
Type I interferon signaling pathway			5.95 × 10^−9^	10

*SLE, systemic lupus erythematosus; NT, normal term; DEG, differentially expressed gene; HIF, Hypoxia-inducible factor; IL, interleukin; GnRH, Gonadotropin-releasing hormone; Th, T helper; MDA, melanoma differentiation-associated gene-5*.

Differential pathway regulations were observed between SLE-F and -M placentas. Regulation of blood circulation, regulation of cellular response to stress, response to glucocorticoid, response to temperature stimulus, response to toxic substance, response to testosterone, rhythmic process, smooth muscle cell proliferation, GnRH (gonadotropin-releasing hormone) signaling pathway, and estrogen signaling pathway were enriched in SLE-F, but not SLE-M placentas (Figures 2B,D; Table 2, Supplementary Tables 5, 6). In contrast, G protein-coupled chemoattractant receptor activity, tumor necrosis factor production, T cell mediated immunity, Th17 cell differentiation, IL-27-mediated signaling pathway, MDA (melanoma differentiation-associated gene)-5 signaling pathway, phospholipase D signaling pathway, transforming growth factor beta receptor signaling pathway, type I interferon signaling pathway, and Wnt signaling pathway were enriched in SLE-M, but not SLE-F placentas (Figures 2B,D; Table 2, Supplementary Tables 5, 6). Figures 2E,F were networks that exhibited the interactions among cluster of genes enriched in biological processes and pathways mentioned above.

Distinct transcriptional profiles in placentas from SLE pregnancies also showed that no significant correlation was found between the relative expression levels of SLE-dysregulated genes and SLEDAI scores (*P* > 0.05, Supplementary Table 7).

## Discussion

To our knowledge, this is the first report that profiles the protein-coding gene expression of human placental tissues from SLE pregnancies using RNA-seq analysis. Overall, more upregulated DEGs were identified than those downregulated. We have further demonstrated that the expression profiles are differentially dysregulated between SLE-F and -M placentas and are associated with differently regulated biological functions and pathways. These data provide clear evidence that SLE differentially regulates the expression of placental protein-coding genes in a fetal sex-dependent manner which may lead to dysregulated placental biological functions.

The mechanisms underlying the fetal sexual dimorphisms of SLE-dysregulated protein-coding gene profiles remain elusive. Expression of DEGs on X-chromosomes is likely to be a major factor that governs these fetal sexual dimorphisms since 4% of DEGs (5 from SLE-F placentas and 16 from SLE-M placentas) are located on the X-chromosome, but no DEGs are on the Y-chromosome. Given that SLE affects women more frequently than men, our current data suggest that the important contribution of X-chromosome-linked genes expression may be associated with the female sex bias in SLE (28). This is in line with a previous study that has shown that a large number of genes that may contribute to the hyperresponsiveness of the female immune system are located on the X chromosome (29). Sex hormones could be another factor that mediates the fetal sexual dimorphisms, as sexual hormones levels are different in the umbilical vein blood of female and male fetuses (30). However, no significant differential expression of androgen or estrogen receptors in SLE-F or -M placentas were detected in this study.

Differential regulation of the mitochondrial genome may also contribute to sexual dimorphisms since our data showed that 4 (*MT-ND2, MT-ND3, MT-CYB*, and *MT-ATP8*; Supplementary Tables 3, 4) SLE-dysregulated genes are located on the mitochondrial genome of SLE-F, but not SLE-M placentas, which comprise 30.8% of mitochondrial protein-coding genes. The primary function of mitochondria is to generate the chemical energy to power cellular responses, which is achieved through the electron-transport chain and oxidative phosphorylation (31). The above four SLE-dysregulated mitochondrial genes participate in encoding polypeptides of the oxidative phosphorylation system (31), suggesting that mitochondrial genes might be actively involved in the pathogenesis of SLE placentas. Mitochondrial dysfunction has been demonstrated to be associated with SLE pathogenesis (32–34). The mitochondrial dysfunction may increase not only oxidative stress but also cell apoptosis in SLE patients and defective bioenergetics (33). Oxidative stress induced by mitochondrial dysfunction is considered an essential downstream contributor for SLE pathogenesis (34). A study has shown that CD4+ T cells from an SLE mouse model have higher basal and activated mitochondrial oxidative metabolism, while inhibition of mitochondrial respiratory-chain complex 1 by treating SLE mice with metformin can prevent autoimmune activation (32). Our findings are in line with the above reports and support the notion that the mitochondrial genome of SLE may contribute to fetal sexual dimorphisms, and mitochondrial dysfunction may be present in SLE placentas.

We have also demonstrated that SLE dysregulates biological processes within placentas in a fetal sex-dependent manner. For instance, regulation of blood circulation was enriched in SLE-F but not in SLE-M placentas. In addition, published studies reported that age-standardized cardiovascular disease incidence, prevalence, and mortality rates are lower in women than men (35, 36). Similar results were reported in SLE cohorts that male lupus patients had more cardiovascular damage than their female counterparts (37). Therefore, the enrichment of regulation of blood circulation suggests a possible protective adaptation of cardiovascular diseases in SLE-F placentas.

Our findings revealed that *MDA-5* was upregulated in SLE-M, but not SLE-F placentas in association with enrichment of MDA-5 [melanoma differentiation-associated gene-5, gene name: *IFIH1* (interferon induced with helicase C domain 1)] signaling pathway only in SLE-M, but not SLE-F placentas. The relationship between MDA-5 and SLE has been proposed by several groups (38, 39). For example, the single nucleotide polymorphisms (SNP) in *MDA-5* has been implicated in the pathogenesis of SLE in Africa-Americans, American, Asian, Brazilian, European, and European-Americans (39). *MDA-5* has also been demonstrated to be associated with increased sensitivity to serum IFN-α and anti-dsDNA antibodies among SLE patients (38). Our results showed, for the first time, the aberrant expression of *MDA-5* in human SLE placentas. Therefore, it is necessary to further investigate the relationship between MDA-5 and SLE pregnancy, especially the function of MDA-5 in SLE placentas.

SLE-F and -M placentas share some common SLE-dysregulated biological processes, e.g., angiogenesis, innate immune responses, and inflammation. This is consistent with the previous observation since these biological processes are essential in placental development and function (40). These data demonstrate that aberrant functions of angiogenesis, innate immune responses, and inflammation may be present in SLE placentas. Specifically, our observations agree with the study that observed a significant increase in angiogenic activity in the serum samples of SLE patients, which was positively associated with SLE disease activity (41, 42). Guilherme et al. reported that serum Vascular Endothelial Growth Factor A (VEGFA) levels were significantly higher in pregnant women with active SLE nephritis than patients with inactive SLE or preeclampsia (42). VEGFA has been demonstrated to be the most potent pro-angiogenic factor and therefore is actively involved in the development of inflammation (43). The gene encoding *VEGFA* is located on chromosome 6 at 6p21.1, one of the major SLE susceptibility loci (44). Another pro-angiogenic counterpart of VEGFA, which is higher in SLE placentas in this study, is CTGF. CTGF, also known as Cellular Communication Network (CCN) Factor 2, belongs to the CCN family. *CTGF* gene is located on chromosome 6 at 6q23.2, which is also closely associated with SLE (45). CTGF is a key regulatory and signaling molecule associated with numerous biological processes, including angiogenesis, wound healing, cell proliferation, tissue regeneration, and fibrosis through interaction with many factors, such as VEGFA and TNF-α (46, 47). Based on our RT-qPCR results, *VEGFA* and *CTGF* mRNA levels were significantly higher in SLE placentas, suggesting that placental angiogenic activity is disrupted in SLE, just like in preeclampsia (48).

Although defined as an autoimmune disease, SLE is characterized by chronic and acute inflammation conditions in multiple organs with the generation of autoantibodies abnormally produced by one's immune system (49). Torricelli et al. reported that IL-17 was increased in serum from pregnant women with SLE (50). Our RNA-seq analysis also showed that the levels of *IL17D*, but not other IL17 family members (e.g., *IL17A, IL17B, IL17C*, and *IL17F)* was elevated in SLE-M placentas, while the IL-17 signaling pathway was enriched in both male and female placentas, supporting that IL-17 signaling pathway is actively involved in the dysregulation of placental immune response in SLE placentas.

Correlation analysis in this study failed to show any correlation between DEGs and SLEDAI scores. However, a small case number (*n* = 8) and narrow SLEDAI scores (ranging from 0 to 6) of SLE patients recruited in this study may have prevented us from elucidating any correlations.

In conclusion, this is the first report of protein-coding gene profiles of placentas tissues from SLE pregnancies with female and male fetuses using RNA-seq analysis. The results indicate that the differential expression of protein-coding genes in the female and male placenta may contribute to the different pathogenesis of SLE pregnancies. There are several limitations in this study, including a relatively small sample size which might not address all sex differences in SLE placental gene expression. In addition, SLEDAI scores of the recruited SLE patients were relatively low, ranging from 0 to 6. Thus, while these low SLEDAI scores may provide a valuable resource, recruiting a large cohort of SLE patients with a broader range of SLEDAI scores is needed to establish a more reliable correlation between DEGs and SLEDAI scores.

## Data Availability Statement

The datasets presented in this study can be found in online repositories. The names of the repository/repositories and accession number(s) can be found in the article/Supplementary Material.

## Ethics Statement

The studies involving human participants were reviewed and approved by the Institutional Review Board of Qilu Hospital, Shandong University and the Scientific and Ethical Committee of Shanghai First Maternity and Infant Hospital affiliated with Tongji University. The patients/participants provided their written informed consent to participate in this study.

## Author Contributions

HHL and LTS collected tissues samples and drafted the manuscript. ST designed the tables and figures. YL participated in manuscript preparation. CF designed the figures and read the manuscript critically. KW collected tissues and read the manuscript critically. CZ analyzed the RNA-seq data and read the manuscript critically. JZ and QS conceived the concept and wrote the manuscript. YJZ designed the work and read the manuscript critically. All authors approved the final version of the manuscript.

## Funding

This study was supported by ECCM Program of Clinical Research Center of Shandong University (2021SDUCRCB010) and Key Technology Research and Development Program of Shandong (CN) (2018GSF118071 to YJZ, and 2018GSF118025 to HHL).

## Conflict of Interest

The authors declare that the research was conducted in the absence of any commercial or financial relationships that could be construed as a potential conflict of interest.

## Publisher's Note

All claims expressed in this article are solely those of the authors and do not necessarily represent those of their affiliated organizations, or those of the publisher, the editors and the reviewers. Any product that may be evaluated in this article, or claim that may be made by its manufacturer, is not guaranteed or endorsed by the publisher.
