# Supplementary material for: Sexual Dimorphisms of Protein-Coding Gene Profiles in Placentas From Women With Systemic Lupus Erythematosus
Source: Front Med (Lausanne). 2022 Mar 15;9:798907. doi: 10.3389/fmed.2022.798907 (PMC8965019; doi:10.3389/fmed.2022.798907)
Supplement: Supplementary file 1 [file Data_Sheet_1.PDF]

TABLE S1. Patient demographics

	Diagnosis	Fetal sex	N	Cesarean section (n)	Maternal BMI	Maternal age (years)	Gestational age (weeks)	Fetal weight (gram)
RNA-seq	NT	Female	3	1	25.97 ± 3.58	30.00 ± 1.73	38.57 ± 0.91	3365.00 ± 271.22
	SLE	Female	3	1	30.44 ± 4.08	32.00 ± 4.00	37.00 ± 1.33	2600.00 ± 500.83
	P-value				0.363	0.711	0.043	0.072
	NT	Male	5	1	30.24 ± 4.55	30.00 ± 1.79	39.57 ± 0.58	3440.00 ± 327.17
	SLE	Male	5	1	25.00 ± 1.12	28.00 ± 2.19	38.57 ± 0.64	3300.00 ± 538.52
	P-value				0.163	0.193	0.273	0.506
RT-qPCR	NT	Female	4	1	27.98 ± 3.20	32.00 ± 1.50	38.64 ± 0.41	3312.50 ± 131.55
	SLE	Female	4	1	31.02 ± 3.57	33.50 ± 3.59	37.14 ± 1.59	2800.00 ± 448.14
	P-value				0.200	0.626	0.064	0.041
	NT	Male	4	1	27.53 ± 4.95	31.00 ± 1.92	38.21 ± 0.55	3535.00 ± 349.82
	SLE	Male	4	1	24.29 ± 0.90	29.00 ± 2.58	38.50 ± 0.55	3100.00 ± 246.22
	P-value				0.486	0.387	0.247	0.193

All values are median ± SD. BMI, body mass index; NT, normal full-term; SLE, systemic lupus erythematosus.

TABLE S2. RT-qPCR primers.

Gene ID	Gene Name	Forward (5'-3')	Reverse (5'-3')
ENSG00000178878	*APOLD1*	TCCTTAGCAAGCATTCGAATCC	TGACTTTGACACCTACCATATTGGAA
ENSG00000125730	*C3*	CTACATCATCGGGAAGGACACT	CATTGTTTCTGGTTCTCTTCGTC
ENSG00000118523	*CTGF*	CAAGACCTGTGCCTGCCATT	TCATGCCATGTCTCCGTACATC
ENSG00000120129	*DUSP1*	AGGCTCTTCACATCCCATTG	AGCCCCTCCCAGAGTTATTG
ENSG00000115414	*FN1*	TGAGGCAACGTGTTATGATGA	TGGCACCGAGATATTCCTTC
ENSG00000136487	*GH2*	CAAAATCGCACAACGATGAC	CCTTGTCCATGTCCTTCCTG
ENSG00000111640	*GAPDH*	TGACTTCAACAGCGACACCCA	CACCCTGTTGCTGTAGCCAAA
ENSG00000196611	*MMP1*	TACGAATTTGCCGACAGAGA	GTCCTTGGGGTATCCGTGTA
ENSG00000170965	*PLAC1*	GTGATCCCAGTGTCATGTGC	CCTGCTCTTGCTGGCTACTC
ENSG00000243137	*PSG4*	CAGACCTCCCCAGCATTTAC	ATATTGTGCCCGTGGGTTAG
ENSG00000112715	*VEGFA*	CCTCACACCATTGAAACCACTA	CAACCACTCACACACACACAAC

TABLE S3. SLE dyregulated protein-coding genes in placentas

Gene ID	Gene location in sex chromosome or mitochondrial DNA	Gene symbol	Female placenta		Male placenta		Comments
			log2 (SLE/NT)	FDR-adjusted *P* -Value	log2 (SLE/NT)	FDR-adjusted *P* -Value	
ENSG00000118523		CTGF	1.84	3.51E-02	1.20	2.30E-03	Differentially expressed in both female and male placentas
ENSG00000142871		CYR61	1.92	2.51E-03	1.31	3.68E-05	Differentially expressed in both female and male placentas
ENSG00000096060		FKBP5	2.54	7.41E-20	2.12	3.27E-04	Differentially expressed in both female and male placentas
ENSG00000177606		JUN	1.98	1.51E-06	1.27	2.52E-04	Differentially expressed in both female and male placentas
ENSG00000119138		KLF9	2.28	4.00E-08	1.73	9.75E-05	Differentially expressed in both female and male placentas
ENSG00000128422		KRT17	2.38	2.23E-02	2.80	2.29E-03	Differentially expressed in both female and male placentas
ENSG00000123358		NR4A1	1.58	1.02E-04	1.49	7.00E-07	Differentially expressed in both female and male placentas
ENSG00000119508		NR4A3	1.78	3.28E-03	1.36	3.33E-03	Differentially expressed in both female and male placentas
ENSG00000099985		OSM	3.04	1.27E-02	2.03	2.50E-04	Differentially expressed in both female and male placentas
ENSG00000143125		PROK1	1.72	1.26E-02	1.64	1.91E-04	Differentially expressed in both female and male placentas
ENSG00000125384		PTGER2	1.58	2.25E-02	1.06	3.44E-02	Differentially expressed in both female and male placentas
ENSG00000073756		PTGS2	2.53	9.42E-03	2.63	4.05E-04	Differentially expressed in both female and male placentas
ENSG00000163661		PTX3	4.23	1.51E-06	3.50	1.13E-03	Differentially expressed in both female and male placentas
ENSG00000108551		RASD1	2.95	7.99E-03	4.16	8.57E-08	Differentially expressed in both female and male placentas
ENSG00000090104		RGS1	4.05	7.13E-08	3.56	1.56E-14	Differentially expressed in both female and male placentas
ENSG00000079215		SLC1A3	1.46	1.01E-04	1.23	3.34E-09	Differentially expressed in both female and male placentas
ENSG00000171621		SPSB1	1.36	4.63E-02	1.10	1.02E-02	Differentially expressed in both female and male placentas
ENSG00000137801		THBS1	1.15	1.89E-02	1.20	2.88E-03	Differentially expressed in both female and male placentas
ENSG00000112715		VEGFA	1.52	7.11E-03	2.04	2.52E-04	Differentially expressed in both female and male placentas
ENSG00000109906		ZBTB16	2.68	1.86E-17	1.69	4.90E-02	Differentially expressed in both female and male placentas

ENSG00000128016	ZFP36	1.83	8.25E-07	1.26	8.58E-05	Differentially expressed in both female and male placentas
ENSG00000280987	MATR3	2.28	7.99E-03	-2.87	2.24E-03	Differentially expressed in both female and male placentas
ENSG00000047457	CP	-3.13	1.75E-02	2.38	2.24E-02	Differentially expressed in both female and male placentas
ENSG00000108679	LGALS3BP	-1.24	1.44E-02	1.19	1.13E-03	Differentially expressed in both female and male placentas
ENSG00000141338	ABCA8	1.52	8.34E-03			Differentially expressed in only female placentas
ENSG00000267426	AC087289.3	6.01	5.24E-03			Differentially expressed in only female placentas
ENSG00000267645	AC105052.3	3.15	1.69E-03			Differentially expressed in only female placentas
ENSG00000185736	ADARB2	2.74	1.26E-02			Differentially expressed in only female placentas
ENSG00000170214	ADRA1B	2.99	2.25E-02			Differentially expressed in only female placentas
ENSG00000178878	APOLD1	1.68	7.90E-06			Differentially expressed in only female placentas
ENSG00000127249	ATP13A4	2.97	3.04E-04			Differentially expressed in only female placentas
ENSG00000125845	BMP2	2.11	2.95E-03			Differentially expressed in only female placentas
ENSG00000168497	CAVIN2	1.39	6.68E-06			Differentially expressed in only female placentas
ENSG00000274276	CBSL	2.68	1.03E-02			Differentially expressed in only female placentas
ENSG00000170160	CCDC144A	2.39	2.88E-03			Differentially expressed in only female placentas
ENSG00000177575	CD163	1.10	2.62E-03			Differentially expressed in only female placentas
ENSG00000221869	CEBPD	1.30	8.15E-06			Differentially expressed in only female placentas
ENSG00000189143	CLDN4	4.42	3.98E-02			Differentially expressed in only female placentas
ENSG00000121898	CPXM2	3.46	1.03E-02			Differentially expressed in only female placentas
ENSG00000197406	DIO3	1.31	3.30E-02			Differentially expressed in only female placentas
ENSG00000120129	DUSP1	1.24	2.03E-02			Differentially expressed in only female placentas
ENSG00000117525	F3	1.15	4.44E-03			Differentially expressed in only female placentas
ENSG00000146192	FGD2	1.17	3.29E-03			Differentially expressed in only female placentas
ENSG00000164379	FOXQ1	2.41	1.31E-02			Differentially expressed in only female placentas

ENSG00000131386		GALNT15	1.23	1.36E-02	Differentially expressed in only female placentas
ENSG00000130037		KCNA5	2.23	1.26E-02	Differentially expressed in only female placentas
ENSG00000180332		KCTD4	2.15	1.36E-02	Differentially expressed in only female placentas
ENSG00000163884		KLF15	3.09	1.78E-07	Differentially expressed in only female placentas
ENSG00000067082		KLF6	1.36	2.95E-03	Differentially expressed in only female placentas
ENSG00000167767		KRT80	1.09	6.32E-03	Differentially expressed in only female placentas
ENSG00000197683		KRTAP26-1	1.33	2.04E-02	Differentially expressed in only female placentas
ENSG00000125148		MT2A	1.10	1.48E-02	Differentially expressed in only female placentas
ENSG00000228253	MT	MT-ATP8	1.04	1.42E-02	Differentially expressed in only female placentas
ENSG00000198727	MT	MT-CYB	1.22	1.52E-02	Differentially expressed in only female placentas
ENSG00000198763	MT	MT-ND2	1.34	1.64E-04	Differentially expressed in only female placentas
ENSG00000198840	MT	MT-ND3	1.14	8.67E-04	Differentially expressed in only female placentas
ENSG00000184022		OR2T10	2.17	3.60E-02	Differentially expressed in only female placentas
ENSG00000196240		OR2T2	2.36	4.66E-02	Differentially expressed in only female placentas
ENSG00000187848		P2RX2	4.45	1.14E-02	Differentially expressed in only female placentas
ENSG00000100767		PAPLN	1.87	1.64E-02	Differentially expressed in only female placentas
ENSG00000004799		PDK4	1.32	1.65E-04	Differentially expressed in only female placentas
ENSG00000179094		PER1	1.92	7.33E-12	Differentially expressed in only female placentas
ENSG00000168490		PHYHIP	1.31	4.65E-02	Differentially expressed in only female placentas
ENSG00000118898		PPL	1.29	2.95E-03	Differentially expressed in only female placentas
ENSG00000183668		PSG9	1.28	2.89E-02	Differentially expressed in only female placentas
ENSG00000072858		SIDT1	2.06	4.03E-02	Differentially expressed in only female placentas
ENSG00000254415		SIGLEC14	4.79	3.29E-03	Differentially expressed in only female placentas
ENSG00000105501		SIGLEC5	2.43	3.10E-02	Differentially expressed in only female placentas

ENSG00000155926		SLA	1.25	1.75E-02	Differentially expressed in only female placentas
ENSG00000158714		SLAMF8	1.80	2.08E-03	Differentially expressed in only female placentas
ENSG00000117479		SLC19A2	1.13	4.45E-04	Differentially expressed in only female placentas
ENSG00000256235		SMIM3	1.17	3.89E-02	Differentially expressed in only female placentas
ENSG00000162804		SNED1	1.49	2.23E-02	Differentially expressed in only female placentas
ENSG00000117155		SSX2IP	1.23	4.03E-02	Differentially expressed in only female placentas
ENSG00000172403		SYNPO2	1.33	4.78E-02	Differentially expressed in only female placentas
ENSG00000131746		TNS4	1.05	6.81E-04	Differentially expressed in only female placentas
ENSG00000157514	X-chromosome	TSC22D3	1.17	8.74E-03	Differentially expressed in only female placentas
ENSG00000036672		USP2	3.19	1.13E-05	Differentially expressed in only female placentas
ENSG00000133316		WDR74	3.58	1.64E-04	Differentially expressed in only female placentas
ENSG00000173175		ADCY5	-1.27	2.04E-02	Differentially expressed in only female placentas
ENSG00000173698	X-chromosome	ADGRG2	-2.36	3.83E-02	Differentially expressed in only female placentas
ENSG00000150594		ADRA2A	-1.44	2.61E-02	Differentially expressed in only female placentas
ENSG00000155189		AGPAT5	-1.20	4.02E-02	Differentially expressed in only female placentas
ENSG00000181754		AMIGO1	-1.76	3.28E-04	Differentially expressed in only female placentas
ENSG00000091879		ANGPT2	-3.57	3.01E-08	Differentially expressed in only female placentas
ENSG00000134343		ANO3	-3.30	2.95E-03	Differentially expressed in only female placentas
ENSG00000165272		AQP3	-1.49	4.06E-03	Differentially expressed in only female placentas
ENSG00000146476		ARMT1	-1.18	6.30E-03	Differentially expressed in only female placentas
ENSG00000100628		ASB2	-2.06	1.57E-08	Differentially expressed in only female placentas
ENSG00000137948		BRDT	-2.29	7.11E-03	Differentially expressed in only female placentas
ENSG00000187699		C2orf88	-1.68	8.83E-04	Differentially expressed in only female placentas
ENSG00000021826		CPS1	-1.95	3.01E-08	Differentially expressed in only female placentas

ENSG00000100368		CSF2RB	-1.10	3.60E-02	Differentially expressed in only female placentas
ENSG00000163739		CXCL1	-1.42	1.81E-02	Differentially expressed in only female placentas
ENSG00000100867		DHRS2	-1.33	8.53E-04	Differentially expressed in only female placentas
ENSG00000126767	X-chromosome	ELK1	-1.18	3.60E-02	Differentially expressed in only female placentas
ENSG00000124019		FAM124B	-1.37	2.97E-03	Differentially expressed in only female placentas
ENSG00000154319		FAM167A	-1.33	1.19E-03	Differentially expressed in only female placentas
ENSG00000179772		FOXS1	-2.07	8.74E-03	Differentially expressed in only female placentas
ENSG00000136487		GH2	-1.17	1.08E-02	Differentially expressed in only female placentas
ENSG00000147536		GINS4	-1.50	2.49E-02	Differentially expressed in only female placentas
ENSG00000170961		HAS2	-2.55	3.17E-02	Differentially expressed in only female placentas
ENSG00000274750		HIST1H3E	-1.81	2.88E-03	Differentially expressed in only female placentas
ENSG00000029559		IBSP	-4.90	4.96E-02	Differentially expressed in only female placentas
ENSG00000159871		LYPD5	-1.99	5.91E-09	Differentially expressed in only female placentas
ENSG00000147381	X-chromosome	MAGEA4	-2.34	4.45E-04	Differentially expressed in only female placentas
ENSG00000166866		MYO1A	-2.96	2.64E-07	Differentially expressed in only female placentas
ENSG00000157064		NMNAT2	-1.39	1.83E-02	Differentially expressed in only female placentas
ENSG00000279012		OR51B2	-2.61	4.30E-02	Differentially expressed in only female placentas
ENSG00000089723		OTUB2	-1.11	1.03E-02	Differentially expressed in only female placentas
ENSG00000090530		P3H2	-1.33	2.91E-02	Differentially expressed in only female placentas
ENSG00000172840		PDP2	-1.70	2.31E-02	Differentially expressed in only female placentas
ENSG00000170965	X-chromosome	PLAC1	-1.11	8.53E-04	Differentially expressed in only female placentas
ENSG00000132972		RNF17	-2.21	2.20E-02	Differentially expressed in only female placentas
ENSG00000127526		SLC35E1	-1.03	2.25E-02	Differentially expressed in only female placentas
ENSG00000163630		SYNPR	-4.29	3.04E-04	Differentially expressed in only female placentas

ENSG00000139973	SYT16	-1.83	3.10E-02			Differentially expressed in only female placentas
ENSG00000142484	TM4SF5	-3.22	9.69E-03			Differentially expressed in only female placentas
ENSG00000160352	ZNF714	-1.83	3.83E-02			Differentially expressed in only female placentas
ENSG00000166535	A2ML1			3.77	5.92E-04	Differentially expressed in only male placentas
ENSG00000188984	AADACL3			5.06	2.47E-08	Differentially expressed in only male placentas
ENSG00000154175	ABI3BP			2.70	4.11E-04	Differentially expressed in only male placentas
ENSG00000265690	AC074143.1			2.22	3.68E-05	Differentially expressed in only male placentas
ENSG00000110455	ACCS			1.01	4.84E-02	Differentially expressed in only male placentas
ENSG00000114948	ADAM23			4.24	2.51E-02	Differentially expressed in only male placentas
ENSG00000134028	ADAMDEC1			1.43	1.04E-02	Differentially expressed in only male placentas
ENSG00000158859	ADAMTS4			1.69	1.23E-02	Differentially expressed in only male placentas
ENSG00000164742	ADCY1			3.01	9.52E-03	Differentially expressed in only male placentas
ENSG00000282608	ADORA3			2.27	2.09E-04	Differentially expressed in only male placentas
ENSG00000153531	ADPRHL1			1.23	3.52E-02	Differentially expressed in only male placentas
ENSG00000184160	ADRA2C			2.70	3.00E-03	Differentially expressed in only male placentas
ENSG00000196526	AFAP1			1.25	1.39E-05	Differentially expressed in only male placentas
ENSG00000185567	AHNAK2			3.96	5.38E-11	Differentially expressed in only male placentas
ENSG00000183773	AIFM3			1.28	3.20E-02	Differentially expressed in only male placentas
ENSG00000263020	AL662899.2			3.48	2.45E-02	Differentially expressed in only male placentas
ENSG00000165092	ALDH1A1			1.73	2.96E-02	Differentially expressed in only male placentas
ENSG00000128918	ALDH1A2			2.58	2.44E-02	Differentially expressed in only male placentas
ENSG00000109107	ALDOC			2.05	3.56E-02	Differentially expressed in only male placentas
ENSG00000179593	ALOX15B			5.52	1.47E-02	Differentially expressed in only male placentas
ENSG00000198796	ALPK2			1.84	2.51E-02	Differentially expressed in only male placentas

ENSG00000174945		AMZ1	3.81	1.22E-15	Differentially expressed in only male placentas
ENSG00000148677		ANKRD1	2.76	2.29E-06	Differentially expressed in only male placentas
ENSG00000180071		ANKRD18A	3.87	1.20E-05	Differentially expressed in only male placentas
ENSG00000185101		ANO9	1.68	1.43E-03	Differentially expressed in only male placentas
ENSG00000011201	X-chromosome	ANOS1	2.37	2.18E-02	Differentially expressed in only male placentas
ENSG00000264230		ANXA8L1	1.00	3.61E-04	Differentially expressed in only male placentas
ENSG00000002726		AOC1	1.86	1.54E-02	Differentially expressed in only male placentas
ENSG00000254979		AP000781.2	5.01	5.31E-15	Differentially expressed in only male placentas
ENSG00000034053		APBA2	2.16	1.60E-02	Differentially expressed in only male placentas
ENSG00000105290		APLP1	3.48	1.33E-02	Differentially expressed in only male placentas
ENSG00000221963		APOL6	1.15	1.51E-05	Differentially expressed in only male placentas
ENSG00000240583		AQP1	1.03	7.59E-05	Differentially expressed in only male placentas
ENSG00000198576		ARC	2.75	2.18E-03	Differentially expressed in only male placentas
ENSG00000111348		ARHGDIB	1.07	2.92E-03	Differentially expressed in only male placentas
ENSG00000136002		ARHGEF4	1.68	4.19E-02	Differentially expressed in only male placentas
ENSG00000172379		ARNT2	1.46	7.77E-03	Differentially expressed in only male placentas
ENSG00000088280		ASAP3	1.63	4.26E-11	Differentially expressed in only male placentas
ENSG00000183734		ASCL2	4.33	2.67E-36	Differentially expressed in only male placentas
ENSG00000128203		ASPHD2	3.30	1.37E-06	Differentially expressed in only male placentas
ENSG00000244617		ASPRV1	1.74	1.27E-02	Differentially expressed in only male placentas
ENSG00000152092		ASTN1	3.00	2.19E-02	Differentially expressed in only male placentas
ENSG00000198513		ATL1	2.24	1.50E-03	Differentially expressed in only male placentas
ENSG00000168874		ATOH8	1.61	3.10E-03	Differentially expressed in only male placentas
ENSG00000166148		AVPR1A	3.54	3.92E-04	Differentially expressed in only male placentas

ENSG00000156966	B3GNT7	3.20	9.13E-10	Differentially expressed in only male placentas
ENSG00000166546	BEAN1	1.20	9.68E-03	Differentially expressed in only male placentas
ENSG00000134107	BHLHE40	1.05	5.94E-05	Differentially expressed in only male placentas
ENSG00000110934	BIN2	1.03	1.82E-04	Differentially expressed in only male placentas
ENSG00000101197	BIRC7	2.28	5.19E-04	Differentially expressed in only male placentas
ENSG00000270181	BIVM-ERCC5	3.93	5.42E-05	Differentially expressed in only male placentas
ENSG00000198797	BRINP2	6.53	1.54E-03	Differentially expressed in only male placentas
ENSG00000235162	C12orf75	1.42	5.77E-03	Differentially expressed in only male placentas
ENSG00000166780	C16orf45	4.25	2.28E-03	Differentially expressed in only male placentas
ENSG00000125730	C3	1.47	2.55E-02	Differentially expressed in only male placentas
ENSG00000074410	CA12	2.86	5.04E-03	Differentially expressed in only male placentas
ENSG00000042493	CAPG	1.63	2.29E-06	Differentially expressed in only male placentas
ENSG00000275385	CCL18	4.64	4.19E-02	Differentially expressed in only male placentas
ENSG00000163823	CCR1	1.01	1.18E-04	Differentially expressed in only male placentas
ENSG00000012124	CD22	1.52	6.20E-03	Differentially expressed in only male placentas
ENSG00000174807	CD248	1.91	9.47E-03	Differentially expressed in only male placentas
ENSG00000103855	CD276	1.14	3.52E-03	Differentially expressed in only male placentas
ENSG00000129226	CD68	1.25	1.26E-03	Differentially expressed in only male placentas
ENSG00000110848	CD69	2.13	4.84E-02	Differentially expressed in only male placentas
ENSG00000112149	CD83	1.27	2.51E-02	Differentially expressed in only male placentas
ENSG00000010278	CD9	1.55	2.79E-07	Differentially expressed in only male placentas
ENSG00000170558	CDH2	3.69	8.15E-04	Differentially expressed in only male placentas
ENSG00000204539	CDSN	5.29	7.03E-03	Differentially expressed in only male placentas
ENSG00000075275	CELSR1	2.33	1.61E-07	Differentially expressed in only male placentas

ENSG00000138135		CH25H	1.82	1.96E-02	Differentially expressed in only male placentas
ENSG00000064886		CHI3L2	2.36	1.01E-02	Differentially expressed in only male placentas
ENSG00000101938	X-chromosome	CHRDL1	10.99	1.55E-06	Differentially expressed in only male placentas
ENSG00000108556		CHRNE	2.03	2.83E-03	Differentially expressed in only male placentas
ENSG00000175040		CHST2	1.72	9.52E-05	Differentially expressed in only male placentas
ENSG00000147119	X-chromosome	CHST7	1.19	3.58E-02	Differentially expressed in only male placentas
ENSG00000179583		CIITA	1.24	3.92E-02	Differentially expressed in only male placentas
ENSG00000138615		CILP	3.33	1.47E-05	Differentially expressed in only male placentas
ENSG00000160161		CILP2	3.20	4.88E-04	Differentially expressed in only male placentas
ENSG00000164442		CITED2	1.76	4.56E-09	Differentially expressed in only male placentas
ENSG00000164007		CLDN19	2.03	5.87E-11	Differentially expressed in only male placentas
ENSG00000258227		CLEC5A	1.73	1.54E-02	Differentially expressed in only male placentas
ENSG00000109684		CLNK	2.62	2.95E-04	Differentially expressed in only male placentas
ENSG00000120885		CLU	1.01	5.16E-04	Differentially expressed in only male placentas
ENSG00000134326		CMPK2	1.74	1.14E-04	Differentially expressed in only male placentas
ENSG00000118432		CNR1	4.11	1.65E-05	Differentially expressed in only male placentas
ENSG00000111799		COL12A1	2.19	1.46E-06	Differentially expressed in only male placentas
ENSG00000065618		COL17A1	2.75	9.22E-08	Differentially expressed in only male placentas
ENSG00000105664		COMP	2.09	4.35E-02	Differentially expressed in only male placentas
ENSG00000145244		CORIN	2.37	2.72E-03	Differentially expressed in only male placentas
ENSG00000178773		CPNE7	5.63	6.42E-04	Differentially expressed in only male placentas
ENSG00000143320		CRABP2	2.24	2.85E-04	Differentially expressed in only male placentas
ENSG00000006016		CRLF1	2.71	6.69E-05	Differentially expressed in only male placentas
ENSG00000175315		CST6	2.00	1.93E-05	Differentially expressed in only male placentas

ENSG00000169862		CTNND2	2.39	2.18E-03	Differentially expressed in only male placentas
ENSG00000163734		CXCL3	3.43	6.07E-04	Differentially expressed in only male placentas
ENSG00000169429		CXCL8	3.61	1.73E-08	Differentially expressed in only male placentas
ENSG00000172215		CXCR6	1.65	6.32E-11	Differentially expressed in only male placentas
ENSG00000095596		CYP26A1	3.39	7.04E-08	Differentially expressed in only male placentas
ENSG00000172817		CYP7B1	4.82	1.12E-03	Differentially expressed in only male placentas
ENSG00000123977		DAW1	4.68	2.29E-06	Differentially expressed in only male placentas
ENSG00000168209		DDIT4	1.66	3.68E-05	Differentially expressed in only male placentas
ENSG00000121690		DEPDC7	3.60	5.61E-08	Differentially expressed in only male placentas
ENSG00000155792		DEPTOR	1.98	1.38E-03	Differentially expressed in only male placentas
ENSG00000211448		DIO2	4.02	1.03E-10	Differentially expressed in only male placentas
ENSG00000107984		DKK1	4.35	5.65E-11	Differentially expressed in only male placentas
ENSG00000171462		DLK2	3.13	2.88E-03	Differentially expressed in only male placentas
ENSG00000161249		DMKN	3.34	4.00E-04	Differentially expressed in only male placentas
ENSG00000163687		DNASE1L3	2.27	9.64E-03	Differentially expressed in only male placentas
ENSG00000187957		DNER	2.48	1.55E-03	Differentially expressed in only male placentas
ENSG00000088538		DOCK3	1.18	3.28E-02	Differentially expressed in only male placentas
ENSG00000183690	X-chromosome	EFHC2	6.53	1.29E-08	Differentially expressed in only male placentas
ENSG00000129521		EGLN3	4.04	5.87E-11	Differentially expressed in only male placentas
ENSG00000120738		EGR1	1.38	2.18E-03	Differentially expressed in only male placentas
ENSG00000179388		EGR3	1.59	8.74E-03	Differentially expressed in only male placentas
ENSG00000167136		ENDOG	1.26	9.64E-03	Differentially expressed in only male placentas
ENSG00000151023		ENKUR	3.85	4.77E-05	Differentially expressed in only male placentas
ENSG00000086289		EPDR1	2.65	1.90E-02	Differentially expressed in only male placentas

ENSG00000133106	EPSTI1	1.34	1.35E-03	Differentially expressed in only male placentas
ENSG00000139055	ERP27	2.25	5.91E-04	Differentially expressed in only male placentas
ENSG00000158220	ESYT3	2.41	5.03E-03	Differentially expressed in only male placentas
ENSG00000157557	ETS2	1.11	1.70E-05	Differentially expressed in only male placentas
ENSG00000012232	EXTL3	1.83	9.66E-09	Differentially expressed in only male placentas
ENSG00000181104	F2R	2.78	6.27E-05	Differentially expressed in only male placentas
ENSG00000184731	FAM110C	5.16	1.30E-03	Differentially expressed in only male placentas
ENSG00000182230	FAM153B	3.89	1.78E-02	Differentially expressed in only male placentas
ENSG00000185442	FAM174B	1.04	1.30E-02	Differentially expressed in only male placentas
ENSG00000183114	FAM43B	5.43	1.47E-05	Differentially expressed in only male placentas
ENSG00000188522	FAM83G	1.19	1.30E-02	Differentially expressed in only male placentas
ENSG00000283486	FAM95C	3.26	3.56E-04	Differentially expressed in only male placentas
ENSG00000086570	FAT2	3.95	6.02E-19	Differentially expressed in only male placentas
ENSG00000136068	FLNB	1.33	5.22E-09	Differentially expressed in only male placentas
ENSG00000102755	FLT1	1.17	1.44E-05	Differentially expressed in only male placentas
ENSG00000115414	FN1	3.31	5.46E-25	Differentially expressed in only male placentas
ENSG00000086205	FOLH1	4.53	2.04E-04	Differentially expressed in only male placentas
ENSG00000170345	FOS	1.14	7.57E-04	Differentially expressed in only male placentas
ENSG00000125740	FOSB	1.60	5.41E-05	Differentially expressed in only male placentas
ENSG00000175592	FOSL1	2.22	1.47E-02	Differentially expressed in only male placentas
ENSG00000150907	FOXO1	1.51	3.19E-06	Differentially expressed in only male placentas
ENSG00000187474	FPR3	1.44	2.18E-03	Differentially expressed in only male placentas
ENSG00000070404	FSTL3	2.83	9.43E-18	Differentially expressed in only male placentas
ENSG00000094755	GABRP	2.57	1.56E-02	Differentially expressed in only male placentas

ENSG00000116717		GADD45A	1.09	2.63E-03	Differentially expressed in only male placentas
ENSG00000144278		GALNT13	3.62	1.17E-02	Differentially expressed in only male placentas
ENSG00000185274		GALNT17	6.98	2.27E-03	Differentially expressed in only male placentas
ENSG00000115339		GALNT3	1.58	1.82E-06	Differentially expressed in only male placentas
ENSG00000139629		GALNT6	1.42	5.28E-05	Differentially expressed in only male placentas
ENSG00000180447		GAS1	2.38	1.53E-03	Differentially expressed in only male placentas
ENSG00000187210		GCNT1	1.46	2.15E-02	Differentially expressed in only male placentas
ENSG00000164949		GEM	1.84	2.24E-03	Differentially expressed in only male placentas
ENSG00000099998		GGT5	2.08	4.13E-04	Differentially expressed in only male placentas
ENSG00000149328		GLB1L2	3.92	1.87E-03	Differentially expressed in only male placentas
ENSG00000139278		GLIPR1	1.51	7.41E-09	Differentially expressed in only male placentas
ENSG00000089639		GMIP	1.05	2.63E-04	Differentially expressed in only male placentas
ENSG00000179921		GPBAR1	1.23	1.03E-03	Differentially expressed in only male placentas
ENSG00000277494		GPIHBP1	1.97	1.18E-02	Differentially expressed in only male placentas
ENSG00000013588		GPRC5A	2.78	6.65E-08	Differentially expressed in only male placentas
ENSG00000185477		GPRIN3	1.27	9.80E-04	Differentially expressed in only male placentas
ENSG00000196208		GREB1	5.73	1.75E-06	Differentially expressed in only male placentas
ENSG00000141449		GREB1L	1.11	8.64E-04	Differentially expressed in only male placentas
ENSG00000125675	X-chromosome	GRIA3	6.24	4.16E-03	Differentially expressed in only male placentas
ENSG00000196277		GRM7	1.97	3.78E-02	Differentially expressed in only male placentas
ENSG00000140511		HAPLN3	2.42	1.65E-05	Differentially expressed in only male placentas
ENSG00000182782		HCAR2	1.55	6.12E-03	Differentially expressed in only male placentas
ENSG00000255398		HCAR3	1.48	1.97E-02	Differentially expressed in only male placentas
ENSG00000130589		HELZ2	1.43	1.58E-04	Differentially expressed in only male placentas

ENSG00000089472	X-chromosome	HEPH	6.24	4.00E-05	Differentially expressed in only male placentas
ENSG00000138642		HERC6	1.34	5.48E-04	Differentially expressed in only male placentas
ENSG00000159399		HK2	2.00	7.07E-05	Differentially expressed in only male placentas
ENSG00000204525		HLA-C	1.26	3.06E-04	Differentially expressed in only male placentas
ENSG00000231389		HLA-DPA1	1.18	1.48E-03	Differentially expressed in only male placentas
ENSG00000196735		HLA-DQA1	2.54	1.90E-03	Differentially expressed in only male placentas
ENSG00000179344		HLA-DQB1	2.24	8.44E-03	Differentially expressed in only male placentas
ENSG00000204287		HLA-DRA	1.06	4.31E-03	Differentially expressed in only male placentas
ENSG00000196126		HLA-DRB1	1.40	2.50E-02	Differentially expressed in only male placentas
ENSG00000204632		HLA-G	5.31	2.86E-12	Differentially expressed in only male placentas
ENSG00000148357		HMCN2	1.68	4.18E-02	Differentially expressed in only male placentas
ENSG00000175879		HOXD8	3.00	2.91E-03	Differentially expressed in only male placentas
ENSG00000128709		HOXD9	4.11	8.70E-03	Differentially expressed in only male placentas
ENSG00000117594		HSD11B1	1.66	2.24E-02	Differentially expressed in only male placentas
ENSG00000004776		HSPB6	1.75	1.19E-02	Differentially expressed in only male placentas
ENSG00000142798		HSPG2	1.54	6.07E-10	Differentially expressed in only male placentas
ENSG00000169495		HTRA4	2.14	1.91E-05	Differentially expressed in only male placentas
ENSG00000142149		HUNK	1.66	2.62E-03	Differentially expressed in only male placentas
ENSG00000165949		IFI27	1.67	1.80E-07	Differentially expressed in only male placentas
ENSG00000068079		IFI35	1.10	3.65E-03	Differentially expressed in only male placentas
ENSG00000137965		IFI44	1.77	2.23E-04	Differentially expressed in only male placentas
ENSG00000137959		IFI44L	2.63	4.92E-07	Differentially expressed in only male placentas
ENSG00000115267		IFIH1	1.02	2.80E-04	Differentially expressed in only male placentas
ENSG00000185745		IFIT1	2.25	1.15E-05	Differentially expressed in only male placentas

ENSG00000119917	IFIT3	1.65	1.70E-04	Differentially expressed in only male placentas
ENSG00000185885	IFITM1	1.61	1.97E-03	Differentially expressed in only male placentas
ENSG00000146678	IGFBP1	11.09	1.45E-06	Differentially expressed in only male placentas
ENSG00000115457	IGFBP2	1.40	4.52E-02	Differentially expressed in only male placentas
ENSG00000141753	IGFBP4	1.24	8.03E-04	Differentially expressed in only male placentas
ENSG00000167779	IGFBP6	3.35	2.37E-03	Differentially expressed in only male placentas
ENSG00000164136	IL15	1.39	1.73E-02	Differentially expressed in only male placentas
ENSG00000172458	IL17D	2.49	8.01E-03	Differentially expressed in only male placentas
ENSG00000163701	IL17RE	2.47	3.62E-08	Differentially expressed in only male placentas
ENSG00000125538	IL1B	2.13	1.27E-02	Differentially expressed in only male placentas
ENSG00000115590	IL1R2	5.34	1.80E-12	Differentially expressed in only male placentas
ENSG00000115602	IL1RL1	2.01	2.01E-04	Differentially expressed in only male placentas
ENSG00000104998	IL27RA	1.29	1.09E-02	Differentially expressed in only male placentas
ENSG00000100385	IL2RB	3.19	1.73E-12	Differentially expressed in only male placentas
ENSG00000163362	INAVA	2.48	1.82E-06	Differentially expressed in only male placentas
ENSG00000123999	INHA	1.03	4.84E-02	Differentially expressed in only male placentas
ENSG00000163083	INHBB	2.72	2.72E-03	Differentially expressed in only male placentas
ENSG00000185507	IRF7	1.23	2.61E-03	Differentially expressed in only male placentas
ENSG00000187608	ISG15	2.66	3.97E-11	Differentially expressed in only male placentas
ENSG00000129009	ISLR	2.88	1.04E-03	Differentially expressed in only male placentas
ENSG00000100593	ISM2	1.58	7.41E-09	Differentially expressed in only male placentas
ENSG00000156886	ITGAD	2.97	1.84E-02	Differentially expressed in only male placentas
ENSG00000115221	ITGB6	1.07	2.18E-03	Differentially expressed in only male placentas
ENSG00000189159	JPT1	1.94	1.73E-05	Differentially expressed in only male placentas

ENSG00000158445		KCNB1	2.74	1.05E-02	Differentially expressed in only male placentas
ENSG00000143473		KCNH1	2.93	2.23E-02	Differentially expressed in only male placentas
ENSG00000153822		KCNJ16	2.45	1.86E-02	Differentially expressed in only male placentas
ENSG00000184261		KCNK12	4.20	5.10E-10	Differentially expressed in only male placentas
ENSG00000100196		KDELR3	1.37	1.14E-03	Differentially expressed in only male placentas
ENSG00000136883		KIF12	4.26	3.50E-09	Differentially expressed in only male placentas
ENSG00000186847		KRT14	6.74	1.15E-05	Differentially expressed in only male placentas
ENSG00000167618		LAIR2	3.29	4.11E-04	Differentially expressed in only male placentas
ENSG00000053747		LAMA3	2.68	2.17E-06	Differentially expressed in only male placentas
ENSG00000091128		LAMB4	1.08	3.08E-02	Differentially expressed in only male placentas
ENSG00000058085		LAMC2	1.36	3.09E-04	Differentially expressed in only male placentas
ENSG00000187942		LDLRAD2	1.65	2.58E-03	Differentially expressed in only male placentas
ENSG00000182195	X-chromosome	LDOC1	2.71	6.82E-03	Differentially expressed in only male placentas
ENSG00000143768		LEFTY2	3.15	4.90E-02	Differentially expressed in only male placentas
ENSG00000131981		LGALS3	1.17	3.35E-05	Differentially expressed in only male placentas
ENSG00000113083		LOX	2.62	3.03E-03	Differentially expressed in only male placentas
ENSG00000064547		LPAR2	1.62	6.85E-05	Differentially expressed in only male placentas
ENSG00000172061		LRRC15	5.89	2.06E-03	Differentially expressed in only male placentas
ENSG00000171017		LRRC8E	1.25	2.24E-02	Differentially expressed in only male placentas
ENSG00000177363		LRRN4CL	4.87	1.81E-02	Differentially expressed in only male placentas
ENSG00000185565		LSAMP	2.60	4.34E-03	Differentially expressed in only male placentas
ENSG00000139329		LUM	1.49	1.25E-02	Differentially expressed in only male placentas
ENSG00000160932		LY6E	1.24	6.01E-03	Differentially expressed in only male placentas
ENSG00000183833		MAATS1	2.17	2.23E-03	Differentially expressed in only male placentas

ENSG00000204103		MAFB		1.12	2.87E-05	Differentially expressed in only male placentas
ENSG00000105695		MAG		3.76	1.06E-02	Differentially expressed in only male placentas
ENSG00000165072		MAMDC2		1.32	7.07E-05	Differentially expressed in only male placentas
ENSG00000069535	X-chromosome	MAOB		2.99	1.72E-02	Differentially expressed in only male placentas
ENSG00000127241		MASP1		1.44	3.12E-02	Differentially expressed in only male placentas
ENSG00000102802		MEDAG		3.44	1.88E-02	Differentially expressed in only male placentas
ENSG00000163975		MELTF		3.05	1.57E-06	Differentially expressed in only male placentas
ENSG00000133816		MICAL2		1.10	1.51E-05	Differentially expressed in only male placentas
ENSG00000099812		MISP		3.52	1.12E-05	Differentially expressed in only male placentas
ENSG00000196611		MMP1		3.54	2.12E-04	Differentially expressed in only male placentas
ENSG00000262406		MMP12		9.05	3.12E-10	Differentially expressed in only male placentas
ENSG00000123342		MMP19		1.76	2.39E-04	Differentially expressed in only male placentas
ENSG00000149573		MPZL2		2.23	5.72E-03	Differentially expressed in only male placentas
ENSG00000011028		MRC2		1.04	2.26E-02	Differentially expressed in only male placentas
ENSG00000187193		MT1X		1.55	2.66E-02	Differentially expressed in only male placentas
ENSG00000157601		MX1		2.46	4.15E-08	Differentially expressed in only male placentas
ENSG00000183486		MX2		2.02	1.48E-04	Differentially expressed in only male placentas
ENSG00000182534		MXRA7		1.89	1.56E-06	Differentially expressed in only male placentas
ENSG00000134323		MYCN		1.08	1.53E-02	Differentially expressed in only male placentas
ENSG00000169994		MYO7B		1.30	1.52E-02	Differentially expressed in only male placentas
ENSG00000177791		MYOZ1		1.64	9.73E-03	Differentially expressed in only male placentas
ENSG00000149294		NCAM1		2.12	1.40E-02	Differentially expressed in only male placentas
ENSG00000124479	X-chromosome	NDP		8.58	2.09E-04	Differentially expressed in only male placentas
ENSG00000162711		NLRP3		1.13	2.62E-03	Differentially expressed in only male placentas

ENSG00000183691		NOG	4.32	4.10E-17	Differentially expressed in only male placentas
ENSG00000007171		NOS2	1.97	1.43E-03	Differentially expressed in only male placentas
ENSG00000185269		NOTUM	3.99	5.35E-28	Differentially expressed in only male placentas
ENSG00000153234		NR4A2	1.21	7.77E-03	Differentially expressed in only male placentas
ENSG00000074590		NUAK1	1.11	4.97E-03	Differentially expressed in only male placentas
ENSG00000163545		NUAK2	1.17	3.47E-04	Differentially expressed in only male placentas
ENSG00000089127		OAS1	1.81	2.01E-05	Differentially expressed in only male placentas
ENSG00000111335		OAS2	1.89	3.41E-07	Differentially expressed in only male placentas
ENSG00000111331		OAS3	1.87	7.37E-07	Differentially expressed in only male placentas
ENSG00000135114		OASL	2.44	7.46E-05	Differentially expressed in only male placentas
ENSG00000127083		OMD	4.65	5.56E-04	Differentially expressed in only male placentas
ENSG00000180914		OXTR	3.10	2.06E-04	Differentially expressed in only male placentas
ENSG00000149380		P4HA3	2.42	9.23E-04	Differentially expressed in only male placentas
ENSG00000142623		PADI1	4.57	3.41E-07	Differentially expressed in only male placentas
ENSG00000122133		PAEP	4.47	1.20E-02	Differentially expressed in only male placentas
ENSG00000059378		PARP12	1.02	9.00E-03	Differentially expressed in only male placentas
ENSG00000138496		PARP9	1.28	9.30E-05	Differentially expressed in only male placentas
ENSG00000105717		PBX4	2.05	3.06E-02	Differentially expressed in only male placentas
ENSG00000183570		PCBP3	2.23	2.50E-02	Differentially expressed in only male placentas
ENSG00000102109	X-chromosome	PCSK1N	4.34	8.62E-07	Differentially expressed in only male placentas
ENSG00000140479		PCSK6	1.40	1.80E-07	Differentially expressed in only male placentas
ENSG00000197646		PDCD1LG2	1.35	3.30E-03	Differentially expressed in only male placentas
ENSG00000186862		PDZD7	1.28	4.50E-02	Differentially expressed in only male placentas
ENSG00000067057		PFKP	1.29	8.39E-03	Differentially expressed in only male placentas

ENSG00000102174	X-chromosome	PHEX		2.23	1.30E-02	Differentially expressed in only male placentas
ENSG00000179761		PIPOX		2.06	5.67E-05	Differentially expressed in only male placentas
ENSG00000146070		PLA2G7		3.29	1.06E-15	Differentially expressed in only male placentas
ENSG00000145287		PLAC8		3.14	7.86E-17	Differentially expressed in only male placentas
ENSG00000011422		PLAUR		1.33	3.03E-03	Differentially expressed in only male placentas
ENSG00000152952		PLOD2		1.10	3.12E-05	Differentially expressed in only male placentas
ENSG00000124225		PMEPA1		1.30	2.51E-02	Differentially expressed in only male placentas
ENSG00000130822	X-chromosome	PNCK		1.36	1.11E-02	Differentially expressed in only male placentas
ENSG00000240694		PNMA2		2.26	4.54E-02	Differentially expressed in only male placentas
ENSG00000100344		PNPLA3		1.66	4.95E-02	Differentially expressed in only male placentas
ENSG00000114631		PODXL2		1.63	2.26E-02	Differentially expressed in only male placentas
ENSG00000196604		POTEF		1.54	4.11E-04	Differentially expressed in only male placentas
ENSG00000196834		POTEI		1.09	3.04E-02	Differentially expressed in only male placentas
ENSG00000119698		PPP4R4		3.36	4.12E-04	Differentially expressed in only male placentas
ENSG00000057657		PRDM1		2.31	1.01E-03	Differentially expressed in only male placentas
ENSG00000186652		PRG2		5.24	4.09E-20	Differentially expressed in only male placentas
ENSG00000172179		PRL		6.47	2.53E-03	Differentially expressed in only male placentas
ENSG00000176532		PRR15		2.14	2.11E-04	Differentially expressed in only male placentas
ENSG00000106772		PRUNE2		6.45	9.65E-11	Differentially expressed in only male placentas
ENSG00000050628		PTGER3		4.22	1.06E-15	Differentially expressed in only male placentas
ENSG00000148344		PTGES		1.05	1.24E-05	Differentially expressed in only male placentas
ENSG00000087494		PTHLH		6.30	4.50E-06	Differentially expressed in only male placentas
ENSG00000120899		PTK2B		1.36	1.17E-05	Differentially expressed in only male placentas
ENSG00000204179		PTPN20		2.00	5.77E-03	Differentially expressed in only male placentas

ENSG00000054356		PTPRN	5.25	7.77E-03	Differentially expressed in only male placentas
ENSG00000153233		PTPRR	3.01	2.38E-04	Differentially expressed in only male placentas
ENSG00000183010		PYCR1	2.36	3.43E-11	Differentially expressed in only male placentas
ENSG00000115828		QPCT	1.42	4.59E-02	Differentially expressed in only male placentas
ENSG00000116260		QSOX1	1.62	4.69E-06	Differentially expressed in only male placentas
ENSG00000132329		RAMP1	2.53	1.61E-04	Differentially expressed in only male placentas
ENSG00000113319		RASGRF2	1.30	5.01E-10	Differentially expressed in only male placentas
ENSG00000078328		RBFOX1	4.10	3.10E-03	Differentially expressed in only male placentas
ENSG00000132819		RBM38	1.04	4.22E-02	Differentially expressed in only male placentas
ENSG00000114115		RBP1	1.99	1.25E-02	Differentially expressed in only male placentas
ENSG00000138207		RBP4	5.19	5.51E-06	Differentially expressed in only male placentas
ENSG00000068615		REEP1	1.38	5.78E-03	Differentially expressed in only male placentas
ENSG00000169891	X-chromosome	REPS2	1.44	1.79E-02	Differentially expressed in only male placentas
ENSG00000116741		RGS2	1.05	9.47E-04	Differentially expressed in only male placentas
ENSG00000117152		RGS4	2.54	1.81E-02	Differentially expressed in only male placentas
ENSG00000119729		RHOQ	1.19	2.69E-02	Differentially expressed in only male placentas
ENSG00000258818		RNASE4	1.53	2.62E-03	Differentially expressed in only male placentas
ENSG00000115963		RND3	1.06	8.47E-03	Differentially expressed in only male placentas
ENSG00000169071		ROR2	1.26	1.95E-03	Differentially expressed in only male placentas
ENSG00000166592		RRAD	1.30	2.05E-02	Differentially expressed in only male placentas
ENSG00000134321		RSAD2	2.04	4.64E-05	Differentially expressed in only male placentas
ENSG00000186907		RTN4RL2	2.85	1.32E-06	Differentially expressed in only male placentas
ENSG00000171509		RXFP1	5.44	3.87E-06	Differentially expressed in only male placentas
ENSG00000205413		SAMD9	1.33	1.82E-05	Differentially expressed in only male placentas

ENSG00000177409	SAMD9L	1.26	2.97E-03	Differentially expressed in only male placentas
ENSG00000168079	SCARA5	4.92	1.70E-07	Differentially expressed in only male placentas
ENSG00000159307	SCUBE1	2.36	2.99E-03	Differentially expressed in only male placentas
ENSG00000146197	SCUBE3	2.38	9.73E-03	Differentially expressed in only male placentas
ENSG00000146555	SDK1	1.11	2.98E-02	Differentially expressed in only male placentas
ENSG00000112902	SEMA5A	1.30	7.70E-03	Differentially expressed in only male placentas
ENSG00000196136	SERPINA3	8.77	1.88E-08	Differentially expressed in only male placentas
ENSG00000206075	SERPINB5	3.02	3.32E-02	Differentially expressed in only male placentas
ENSG00000106366	SERPINE1	1.51	1.17E-04	Differentially expressed in only male placentas
ENSG00000135919	SERPINE2	2.65	3.13E-08	Differentially expressed in only male placentas
ENSG00000149131	SERPING1	1.42	2.29E-06	Differentially expressed in only male placentas
ENSG00000175793	SFN	2.75	3.48E-02	Differentially expressed in only male placentas
ENSG00000104332	SFRP1	2.06	2.79E-07	Differentially expressed in only male placentas
ENSG00000131370	SH3BP5	1.21	2.88E-04	Differentially expressed in only male placentas
ENSG00000174705	SH3PXD2B	1.39	2.77E-05	Differentially expressed in only male placentas
ENSG00000138944	SHISAL1	6.90	1.08E-04	Differentially expressed in only male placentas
ENSG00000142178	SIK1	1.61	1.46E-06	Differentially expressed in only male placentas
ENSG00000275993	SIK1B	2.02	2.88E-12	Differentially expressed in only male placentas
ENSG00000070915	SLC12A3	3.53	7.00E-04	Differentially expressed in only male placentas
ENSG00000221955	SLC12A8	3.36	2.43E-06	Differentially expressed in only male placentas
ENSG00000108932	SLC16A6	1.73	3.91E-05	Differentially expressed in only male placentas
ENSG00000197496	SLC2A10	1.08	5.43E-03	Differentially expressed in only male placentas
ENSG00000173262	SLC2A14	3.25	1.79E-03	Differentially expressed in only male placentas
ENSG00000104635	SLC39A14	1.53	2.05E-06	Differentially expressed in only male placentas

ENSG00000033867		SLC4A7	1.32	2.70E-09	Differentially expressed in only male placentas
ENSG00000132164		SLC6A11	2.49	1.96E-03	Differentially expressed in only male placentas
ENSG00000174640		SLCO2A1	1.02	4.90E-02	Differentially expressed in only male placentas
ENSG00000124107		SLPI	4.65	8.74E-04	Differentially expressed in only male placentas
ENSG00000088826		SMOX	1.65	3.29E-02	Differentially expressed in only male placentas
ENSG00000065609		SNAP91	4.40	2.67E-03	Differentially expressed in only male placentas
ENSG00000112096		SOD2	1.09	2.46E-02	Differentially expressed in only male placentas
ENSG00000109610		SOD3	1.01	3.13E-02	Differentially expressed in only male placentas
ENSG00000176170		SPHK1	1.84	7.14E-04	Differentially expressed in only male placentas
ENSG00000152377		SPOCK1	2.54	6.22E-03	Differentially expressed in only male placentas
ENSG00000118785		SPP1	1.22	4.59E-02	Differentially expressed in only male placentas
ENSG00000111728		ST8SIA1	1.69	1.59E-02	Differentially expressed in only male placentas
ENSG00000136011		STAB2	6.26	6.52E-10	Differentially expressed in only male placentas
ENSG00000115415		STAT1	1.04	3.47E-04	Differentially expressed in only male placentas
ENSG00000196562		SULF2	1.40	5.64E-03	Differentially expressed in only male placentas
ENSG00000164744		SUN3	2.74	3.92E-02	Differentially expressed in only male placentas
ENSG00000149043		SYT8	3.49	7.52E-09	Differentially expressed in only male placentas
ENSG00000166863		TAC3	5.13	1.82E-05	Differentially expressed in only male placentas
ENSG00000168394		TAP1	1.41	5.57E-07	Differentially expressed in only male placentas
ENSG00000204267		TAP2	1.34	1.48E-06	Differentially expressed in only male placentas
ENSG00000148737		TCF7L2	1.01	7.71E-05	Differentially expressed in only male placentas
ENSG00000102265	X-chromosome	TIMP1	1.26	1.44E-05	Differentially expressed in only male placentas
ENSG00000100234		TIMP3	2.34	1.38E-09	Differentially expressed in only male placentas
ENSG00000163659		TIPARP	1.39	9.80E-04	Differentially expressed in only male placentas

ENSG00000104953		TLE6	1.35	3.29E-04	Differentially expressed in only male placentas
ENSG00000139364		TMEM132B	4.03	1.50E-03	Differentially expressed in only male placentas
ENSG00000181234		TMEM132C	2.70	6.82E-03	Differentially expressed in only male placentas
ENSG00000181458		TMEM45A	1.83	1.76E-05	Differentially expressed in only male placentas
ENSG00000147027	X-chromosome	TMEM47	3.02	1.75E-02	Differentially expressed in only male placentas
ENSG00000105696		TMEM59L	4.81	1.65E-02	Differentially expressed in only male placentas
ENSG00000041982		TNC	1.79	4.43E-03	Differentially expressed in only male placentas
ENSG00000120949		TNFRSF8	3.13	3.84E-02	Differentially expressed in only male placentas
ENSG00000121858		TNFSF10	1.20	6.63E-03	Differentially expressed in only male placentas
ENSG00000120337		TNFSF18	2.61	4.10E-02	Differentially expressed in only male placentas
ENSG00000130598		TNNI2	3.53	5.01E-08	Differentially expressed in only male placentas
ENSG00000106785		TRIM14	1.51	1.14E-04	Differentially expressed in only male placentas
ENSG00000119283		TRIM67	4.08	1.34E-02	Differentially expressed in only male placentas
ENSG00000206557		TRIM71	2.25	1.95E-07	Differentially expressed in only male placentas
ENSG00000117472		TSPAN1	3.26	9.35E-03	Differentially expressed in only male placentas
ENSG00000168234		TTC39C	1.30	8.36E-04	Differentially expressed in only male placentas
ENSG00000118271		TTR	3.69	3.82E-02	Differentially expressed in only male placentas
ENSG00000137267		TUBB2A	1.49	3.62E-08	Differentially expressed in only male placentas
ENSG00000156587		UBE2L6	1.17	5.12E-04	Differentially expressed in only male placentas
ENSG00000154277		UCHL1	1.99	4.30E-02	Differentially expressed in only male placentas
ENSG00000109814		UGDH	1.06	3.09E-03	Differentially expressed in only male placentas
ENSG00000111981		ULBP1	3.56	4.18E-02	Differentially expressed in only male placentas
ENSG00000184979		USP18	2.49	1.92E-06	Differentially expressed in only male placentas
ENSG00000132821		VSTM2L	4.38	4.28E-06	Differentially expressed in only male placentas

ENSG00000169884		WNT10B	5.27	1.48E-04	Differentially expressed in only male placentas
ENSG00000162552		WNT4	2.83	7.02E-03	Differentially expressed in only male placentas
ENSG00000143816		WNT9A	1.70	8.34E-04	Differentially expressed in only male placentas
ENSG00000184937		WT1	4.28	1.43E-03	Differentially expressed in only male placentas
ENSG00000132530		XAF1	1.16	8.83E-03	Differentially expressed in only male placentas
ENSG00000173578		XCR1	2.97	2.90E-11	Differentially expressed in only male placentas
ENSG00000177494		ZBED2	3.64	2.93E-02	Differentially expressed in only male placentas
ENSG00000204644		ZFP57	3.82	5.45E-03	Differentially expressed in only male placentas
ENSG00000141497		ZMYND15	1.41	1.37E-02	Differentially expressed in only male placentas
ENSG00000101493		ZNF516	1.31	4.05E-02	Differentially expressed in only male placentas
ENSG00000283088		AC010487.3	-7.62	4.73E-02	Differentially expressed in only male placentas
ENSG00000273496		AC011841.1	-3.15	7.36E-03	Differentially expressed in only male placentas
ENSG00000283515		AC020915.5	-2.19	4.18E-02	Differentially expressed in only male placentas
ENSG00000198211		AC092143.1	-5.43	4.42E-10	Differentially expressed in only male placentas
ENSG00000135298		ADGRB3	-2.46	2.95E-02	Differentially expressed in only male placentas
ENSG00000214595		EML6	-1.29	2.60E-03	Differentially expressed in only male placentas
ENSG00000010932		FMO1	-2.11	4.97E-02	Differentially expressed in only male placentas
ENSG00000109158		GABRA4	-1.26	1.57E-02	Differentially expressed in only male placentas
ENSG00000171189		GRIK1	-2.68	4.50E-02	Differentially expressed in only male placentas
ENSG00000172789		HOXC5	-1.34	1.81E-02	Differentially expressed in only male placentas
ENSG00000133124	X-chromosome	IRS4	-2.54	4.58E-02	Differentially expressed in only male placentas
ENSG00000162267		ITIH3	-2.15	4.64E-02	Differentially expressed in only male placentas
ENSG00000147509		RGS20	-1.55	3.42E-02	Differentially expressed in only male placentas
ENSG00000115268		RPS15	-1.19	8.80E-08	Differentially expressed in only male placentas

ENSG00000101276	SLC52A3	-1.20	1.52E-02	Differentially expressed in only male placentas
ENSG00000072041	SLC6A15	-1.23	5.92E-04	Differentially expressed in only male placentas
ENSG00000104321	TRPA1	-2.97	1.01E-02	Differentially expressed in only male placentas
ENSG00000179909	ZNF154	-1.23	2.62E-02	Differentially expressed in only male placentas
ENSG00000188629	ZNF177	-2.41	3.18E-02	Differentially expressed in only male placentas

TABLE S4. SLE dyregulated protein-coding genes located in sex chromosome or mitochondrial DNA

Gene ID	Gene location in sex chromosome or mitochondrial DNA	Gene symbol	Female placenta		Male placenta		Comments
			log2 (SLE/NT)	FDR-adjusted *P* -Value	log2 (SLE/NT)	FDR-adjusted *P* -Value	
ENSG00000228253	MT	MT-ATP8	1.04	1.42E-02			Differentially expressed in only female placentas
ENSG00000198727	MT	MT-CYB	1.22	1.52E-02			Differentially expressed in only female placentas
ENSG00000198763	MT	MT-ND2	1.34	1.64E-04			Differentially expressed in only female placentas
ENSG00000198840	MT	MT-ND3	1.14	8.67E-04			Differentially expressed in only female placentas
ENSG00000157514	X-chromosome	TSC22D3	1.17	8.74E-03			Differentially expressed in only female placentas
ENSG00000173698	X-chromosome	ADGRG2	-2.36	3.83E-02			Differentially expressed in only female placentas
ENSG00000126767	X-chromosome	ELK1	-1.18	3.60E-02			Differentially expressed in only female placentas
ENSG00000147381	X-chromosome	MAGEA4	-2.34	4.45E-04			Differentially expressed in only female placentas
ENSG00000170965	X-chromosome	PLAC1	-1.11	8.53E-04			Differentially expressed in only female placentas
ENSG00000011201	X-chromosome	ANOS1			2.37	2.18E-02	Differentially expressed in only male placentas
ENSG00000101938	X-chromosome	CHRDL1			10.99	1.55E-06	Differentially expressed in only male placentas
ENSG00000147119	X-chromosome	CHST7			1.19	3.58E-02	Differentially expressed in only male placentas
ENSG00000183690	X-chromosome	EFHC2			6.53	1.29E-08	Differentially expressed in only male placentas
ENSG00000125675	X-chromosome	GRIA3			6.24	4.16E-03	Differentially expressed in only male placentas
ENSG00000089472	X-chromosome	HEPH			6.24	4.00E-05	Differentially expressed in only male placentas
ENSG00000182195	X-chromosome	LDOC1			2.71	6.82E-03	Differentially expressed in only male placentas
ENSG00000069535	X-chromosome	MAOB			2.99	1.72E-02	Differentially expressed in only male placentas
ENSG00000124479	X-chromosome	NDP			8.58	2.09E-04	Differentially expressed in only male placentas
ENSG00000102109	X-chromosome	PCSK1N			4.34	8.62E-07	Differentially expressed in only male placentas
ENSG00000102174	X-chromosome	PHEX			2.23	1.30E-02	Differentially expressed in only male placentas
ENSG00000130822	X-chromosome	PNCK			1.36	1.11E-02	Differentially expressed in only male placentas
ENSG00000169891	X-chromosome	REPS2			1.44	1.79E-02	Differentially expressed in only male placentas
ENSG00000102265	X-chromosome	TIMP1			1.26	1.44E-05	Differentially expressed in only male placentas
ENSG00000147027	X-chromosome	TMEM47			3.02	1.75E-02	Differentially expressed in only male placentas
ENSG00000133124	X-chromosome	IRS4			-2.54	4.58E-02	Differentially expressed in only male placentas

TABLE S5. SLE dysregulated biological functions in female placentas

Category	Term	Description	*P* value	Symbols
GO Biological Processes	GO:0036293	response to decreased oxygen levels	1.09E-07	ANGPT2,AQP3,BMP2,CCN2,KCNA5,CYTB,PTGS2,THBS1,VEGFA, P2RX2,APOLD1,JUN,PPL,SLC1A3,ANO3,ADRA2A,F3,GH2,PDK4,SLA, NR4A3,ADCY5
GO Biological Processes	GO:0048545	response to steroid hormone	1.82E-07	KLF9,CLDN4,CPS1,CCN2,DUSP1,ND3,PER1,PTGS2,THBS1,ZFP36, NR4A3,ADRA2A,GH2,NR4A1,PDK4,PTGER2,ADCY5,BMP2,ELK1, SLC1A3,CXCL1,HAS2
GO Biological Processes	GO:0070482	response to oxygen levels	2.30E-07	ANGPT2,AQP3,BMP2,CCN2,KCNA5,CYTB,PTGS2,THBS1,VEGFA, P2RX2,APOLD1
GO Biological Processes	GO:0001666	response to hypoxia	6.53E-07	ANGPT2,AQP3,BMP2,KCNA5,CYTB,PTGS2,THBS1,VEGFA,P2RX2, APOLD1
GO Biological Processes	GO:1901654	response to ketone	1.46E-06	ADCY5,KLF9,CLDN4,CPS1,DUSP1,ELK1,PTGER2,THBS1
GO Biological Processes	GO:0009612	response to mechanical stimulus	3.51E-06	ANGPT2,JUN,KCNA5,PPL,PTGS2,SLC1A3,THBS1,ANO3
GO Biological Processes	GO:0038066	p38MAPK cascade	3.66E-06	BMP2,DUSP1,PER1,VEGFA,ZFP36,ADRA1B,ADRA2A, CCN2,CCN1,OSM,THBS1,PROK1,FGD2,GH2,PTGS2,NR4A3,OTUB2, ARMT1
GO Biological Processes	GO:0032870	cellular response to hormone stimulus	6.43E-06	ADRA2A,KLF9,CPS1,DUSP1,GH2,NR4A1,ND3,PDK4, PER1,PTGER2,ZFP36,NR4A3
GO Biological Processes	GO:0031960	response to corticosteroid	6.52E-06	KLF9,CPS1,CCN2,DUSP1,ND3,PTGS2,ZFP36

GO Biological Processes	GO:0070848	response to growth factor	9.67E-06	ANGPT2,BMP2,CPS1,CCN2,ELK1,HAS2,NR4A1,IBSP,CCN1,JUN,THBS1,VEGFA,ZFP36,F3,LGALS3BP,PTX3,P3H2,PAPLN,SNED1,AMIGO1, DUSP1,ND2,PDK4,CBSL
GO Biological Processes	GO:0071774	response to fibroblast growth factor	1.07E-05	CPS1,CCN2,ELK1,NR4A1,THBS1,ZFP36
GO Biological Processes	GO:0046683	response to organophosphorus	1.93E-05	CPS1,DUSP1,JUN,PER1,PTGS2,P2RX2,ANGPT2,ELK1,ND3,SLC1A3, USP2,KCNA5,NR4A3,DHRS2
GO Biological Processes	GO:0001568	blood vessel development	1.99E-05	ANGPT2,CCN2,F3,FOXS1,HAS2,NR4A1,CCN1,JUN,PTGS2,THBS1, VEGFA,APOLD1,PROK1,BMP2,KLF9,ZFP36,NR4A3,ADRA2A,CLDN4, PPL,SLC1A3,PAPLN,AQP3,OSM,CD163,PSG9,KCNA5,PTX3,DUSP1,
GO Biological Processes	GO:0000165	MAPK cascade	2.10E-05	ADRA1B,ADRA2A,BMP2,CCN2,DUSP1,CCN1,OSM,PER1,THBS1, VEGFA,ZFP36,PROK1,FGD2
GO Biological Processes	GO:0043408	regulation of MAPK cascade	2.49E-05	ADRA1B,ADRA2A,BMP2,CCN2,DUSP1,CCN1,OSM,PER1,THBS1, VEGFA,PROK1,FGD2
GO Biological Processes	GO:0043281	regulation of cysteine-type endopeptidase activity involved in apoptotic process	3.06E-05	CCN2,F3,NR4A1,CCN1,PTGS2,THBS1,VEGFA
GO Biological Processes	GO:0071363	cellular response to growth factor stimulus	3.08E-05	ANGPT2,BMP2,CPS1,CCN2,HAS2,NR4A1,IBSP,CCN1,JUN,THBS1,VEGFA,ZFP36
GO Biological Processes	GO:0048514	blood vessel morphogenesis	3.26E-05	ANGPT2,CCN2,F3,HAS2,NR4A1,CCN1,JUN,PTGS2,THBS1,VEGFA,APOLD1,PROK1
GO Biological Processes	GO:0071407	cellular response to organic cyclic compound	3.37E-05	ADCY5,BMP2,KLF9,CPS1,ELK1,ND3,PER1,PTGS2,SLC1A3,ZFP36, NR4A3
GO Biological Processes	GO:0051384	response to glucocorticoid	3.69E-05	KLF9,CPS1,DUSP1,ND3,PTGS2,ZFP36

GO Biological Processes	GO:0014074	response to purine-containing compound	3.83E-05	CPS1,DUSP1,JUN,PER1,PTGS2,P2RX2
GO Biological Processes	GO:0048511	rhythmic process	3.86E-05	KLF9,CLDN4,HAS2,JUN,PER1,PTX3,USP2,PROK1
GO Biological Processes	GO:0050678	regulation of epithelial cell proliferation	3.94E-05	BMP2,KLF9,F3,HAS2,NR4A1,THBS1,VEGFA,ZFP36,NR4A3
GO Biological Processes	GO:0001525	angiogenesis	4.24E-05	ANGPT2,CCN2,F3,NR4A1,CCN1,JUN,PTGS2,THBS1,VEGFA,APOLD1, PROK1
GO Biological Processes	GO:0070293	renal absorption	4.56E-05	AQP3,CLDN4,HAS2,ADRA2A,MYO1A,SLC1A3,ABCA8,CCN2,PDK4,PTGS2,VEGFA,NR4A3
GO Biological Processes	GO:1900744	regulation of p38MAPK cascade	5.19E-05	BMP2,DUSP1,PER1,VEGFA
GO Biological Processes	GO:0043434	response to peptide hormone	6.23E-05	CPS1,CCN2,GH2,NR4A1,CYTB,PDK4,PTGS2,NR4A3,KLF15,AQP3,JUN,ZFP36,CBSL,BMP2,CSF2RB,FKBP5,CXCL1,RGS1,SLAMF8,ADCY5, SLC1A3,AMIGO1,PTX3
GO Biological Processes	GO:2000116	regulation of cysteine-type endopeptidase activity	6.36E-05	CCN2,F3,NR4A1,CCN1,PTGS2,THBS1,VEGFA
GO Biological Processes	GO:0007169	transmembrane receptor protein tyrosine kinase signaling pathway	6.46E-05	ADRA2A,ANGPT2,BMP2,CCN2,F3,GH2,PDK4,SLA,THBS1,VEGFA,NR4A3
GO Biological Processes	GO:0009743	response to carbohydrate	9.54E-05	ADCY5,ADRA2A,ANGPT2,CCN2,PTGS2,THBS1,P2RX2
GO Biological Processes	GO:0009611	response to wounding	1.06E-04	ADRA2A,CLDN4,CCN2,F3,CCN1,PPL,SLC1A3,THBS1,VEGFA,ZFP36

GO Biological Processes	GO:0044344	cellular response to fibroblast growth factor stimulus	1.07E-04	CPS1,CCN2,NR4A1,THBS1,ZFP36
GO Biological Processes	GO:0090303	positive regulation of wound healing	1.08E-04	ADRA2A,CLDN4,F3,THBS1
GO Biological Processes	GO:0030335	positive regulation of cell migration	1.12E-04	ADRA2A,BMP2,CLDN4,F3,HAS2,CCN1,PTGS2,THBS1,VEGFA,NR4A3
GO Biological Processes	GO:0043405	regulation of MAP kinase activity	1.12E-04	ADRA2A,BMP2,DUSP1,THBS1,VEGFA,FGD2
GO Biological Processes	GO:0050673	epithelial cell proliferation	1.13E-04	BMP2,KLF9,F3,HAS2,NR4A1,THBS1,VEGFA,ZFP36,NR4A3
GO Biological Processes	GO:0050801	ion homeostasis	1.16E-04	ADCY5,ADRA1B,CP,CPS1,KCNA5,MT2A,PDK4,PTGER2,SLC1A3,P2RX2,SLAMF8,ATP13A4,AQP3,CLDN4,ATP8,CYTB,ANO3
GO Biological Processes	GO:0003012	muscle system process	1.17E-04	ADRA1B,ADRA2A,CCN2,NR4A1,KCNA5,PTGS2,NR4A3,P2RX2,KLF15
GO Biological Processes	GO:0003018	vascular process in circulatory system	1.18E-04	ADRA1B,ADRA2A,CPS1,KCNA5,PTGS2,SLC1A3,VEGFA
GO Biological Processes	GO:0009314	response to radiation	1.27E-04	ANGPT2,DUSP1,ELK1,JUN,ND3,PER1,PTGS2,SLC1A3,USP2
GO Biological Processes	GO:0052547	regulation of peptidase activity	1.42E-04	CLDN4,CCN2,F3,NR4A1,CCN1,PTGS2,THBS1,VEGFA,PAPLN
GO Biological Processes	GO:0010035	response to inorganic substance	1.45E-04	AQP3,CPS1,DUSP1,JUN,KCNA5,MT2A,CYTB,PTGS2,THBS1,NR4A3, SLC1A3, SLAMF8,DHRS2,TSC22D3,ANO3

GO Biological Processes	GO:0071396	cellular response to lipid	1.47E-04	ADCY5,KLF9,CPS1,ELK1,CXCL1,ND3,PDK4,PER1,ZFP36,NR4A3
GO Biological Processes	GO:0070542	response to fatty acid	1.48E-04	CPS1,CCN2,PDK4,PTGS2
GO Biological Processes	GO:0050922	negative regulation of chemotaxis	1.58E-04	ANGPT2,DUSP1,THBS1,SLAMF8,F3,VEGFA,ELK1,CXCL1,NR4A1,CCN1,IBSP,JUN,ASB2,BMP2
GO Biological Processes	GO:2000147	positive regulation of cell motility	1.62E-04	ADRA2A,BMP2,CLDN4,F3,HAS2,CCN1,PTGS2,THBS1,VEGFA,NR4A3
GO Biological Processes	GO:0045600	positive regulation of fat cell differentiation	1.67E-04	BMP2,PTGS2,ZFP36,ZBTB16,CCN2,DUSP1,ELK1,NR4A1,CCN1,JUN,THBS1,NR4A3,ADCY5,PTGER2,VEGFA,PDK4,ADRA2A,SLC1A3,USP2, P2RX2,KLF9,CXCL1,OSM,DHRS2, P3H2
GO Biological Processes	GO:0019229	regulation of vasoconstriction	1.67E-04	ADRA1B,ADRA2A,KCNA5,PTGS2
GO Biological Processes	GO:0050926	regulation of positive chemotaxis	1.69E-04	ANGPT2,F3,VEGFA
GO Biological Processes	GO:0010038	response to metal ion	1.71E-04	AQP3,CPS1,DUSP1,JUN,MT2A,CYTB,PTGS2,THBS1
GO Biological Processes	GO:0043410	positive regulation of MAPK cascade	1.86E-04	ADRA1B,ADRA2A,BMP2,CCN2,OSM,THBS1,VEGFA,PROK1,FGD2
GO Biological Processes	GO:0007263	nitric oxide mediated signal transduction	1.90E-04	THBS1,VEGFA,RASD1,P3H2,ADCY5,PER1
GO Biological Processes	GO:0051272	positive regulation of cellular component movement	1.91E-04	ADRA2A,BMP2,CLDN4,F3,HAS2,CCN1,PTGS2,THBS1,VEGFA,NR4A3

GO Biological Processes	GO:0040017	positive regulation of locomotion	1.96E-04	ADRA2A,BMP2,CLDN4,F3,HAS2,CCN1,PTGS2,THBS1,VEGFA,NR4A3
GO Biological Processes	GO:0003013	circulatory system process	2.02E-04	ADRA1B,ADRA2A,CPS1,CCN2,KCNA5,PTGER2,PTGS2,SLC1A3,VEGFA,P2RX2
GO Biological Processes	GO:1903036	positive regulation of response to wounding	2.34E-04	ADRA2A,CLDN4,F3,THBS1
GO Biological Processes	GO:0055080	cation homeostasis	2.40E-04	ADCY5,ADRA1B,CP,KCNA5,MT2A,PDK4,PTGER2,SLC1A3,P2RX2,SLAMF8,ATP13A4
GO Biological Processes	GO:0050679	positive regulation of epithelial cell proliferation	2.45E-04	BMP2,F3,HAS2,NR4A1,VEGFA,NR4A3
GO Biological Processes	GO:0061035	regulation of cartilage development	2.47E-04	BMP2,CCN2,CCN1,ZBTB16,HAS2,IBSP,PTX3,PAPLN,ANGPT2,DUSP1, THBS1,VEGFA,NR4A3,KLF15,PTGS2,MATR3,ASB2
GO Biological Processes	GO:0009991	response to extracellular stimulus	2.59E-04	AQP3,CPS1,GH2,JUN,CYTB,PDK4,PTGS2,ZFP36,CBSL
GO Biological Processes	GO:0010942	positive regulation of cell death	2.62E-04	BMP2,CCN2,DUSP1,ELK1,NR4A1,CCN1,JUN,PTGS2,THBS1,ZBTB16
GO Biological Processes	GO:0007623	circadian rhythm	2.65E-04	KLF9,CLDN4,JUN,PER1,USP2,PROK1
GO Biological Processes	GO:0030198	extracellular matrix organization	2.76E-04	BMP2,CCN2,HAS2,IBSP,CCN1,PTX3,PAPLN
GO Biological Processes	GO:1901652	response to peptide	2.78E-04	CPS1,CCN2,GH2,NR4A1,CYTB,PDK4,PTGS2,NR4A3,KLF15

GO Biological Processes	GO:0043062	extracellular structure organization	2.82E-04	BMP2,CCN2,HAS2,IBSP,CCN1,PTX3,PAPLN
GO Biological Processes	GO:0098771	inorganic ion homeostasis	2.82E-04	ADCY5,ADRA1B,CP,KCNA5,MT2A,PDK4,PTGER2,SLC1A3,P2RX2, SLAMF8,ATP13A4
GO Biological Processes	GO:0009617	response to bacterium	2.92E-04	BMP2,CPS1,CSF2RB,FKBP5,CXCL1,NR4A1,JUN,PTGS2,RGS1,ZFP36 , SLAMF8
GO Biological Processes	GO:0045229	external encapsulating structure organization	2.93E-04	BMP2,CCN2,HAS2,IBSP,CCN1,PTX3,PAPLN
GO Biological Processes	GO:0008015	blood circulation	2.99E-04	ADRA1B,ADRA2A,CPS1,CCN2,KCNA5,PTGER2,PTGS2,VEGFA,P2R X2
GO Biological Processes	GO:0097746	blood vessel diameter maintenance	3.13E-04	ADRA1B,ADRA2A,CPS1,KCNA5,PTGS2
GO Biological Processes	GO:0035296	regulation of tube diameter	3.13E-04	ADRA1B,ADRA2A,CPS1,KCNA5,PTGS2
GO Biological Processes	GO:0035767	endothelial cell chemotaxis	3.18E-04	NR4A1,THBS1,VEGFA
GO Biological Processes	GO:0061036	positive regulation of cartilage development	3.18E-04	BMP2,CCN1,ZBTB16
GO Biological Processes	GO:0043154	negative regulation of cysteine-type endopeptidase activity involved in apoptotic process	3.19E-04	NR4A1,PTGS2,THBS1,VEGFA
GO Biological Processes	GO:0009746	response to hexose	3.23E-04	ADCY5,ADRA2A,ANGPT2,CCN2,PTGS2,THBS1

GO Biological Processes	GO:0035150	regulation of tube size	3.24E-04	ADRA1B,ADRA2A,CPS1,KCNA5,PTGS2
GO Biological Processes	GO:0071417	cellular response to organonitrogen compound	3.33E-04	ADCY5,CPS1,GH2,NR4A1,PDK4,PTGS2,SLC1A3,NR4A3,KLF15,AMIGO1
GO Biological Processes	GO:0050920	regulation of chemotaxis	3.56E-04	ANGPT2,DUSP1,F3,THBS1,VEGFA,SLAMF8
GO Biological Processes	GO:0007189	adenylate cyclase-activating G protein-coupled receptor signaling pathway	3.68E-04	ADCY5,ADRA1B,ADRA2A,PTGER2,ADGRG2,RGS1,THBS1,P2RX2, GH2
GO Biological Processes	GO:0034284	response to monosaccharide	3.73E-04	ADCY5,ADRA2A,ANGPT2,CCN2,PTGS2,THBS1
GO Biological Processes	GO:0030003	cellular cation homeostasis	3.91E-04	ADCY5,ADRA1B,CP,KCNA5,MT2A,PTGER2,SLC1A3,P2RX2,SLAMF8, ATP13A4
GO Biological Processes	GO:0009416	response to light stimulus	3.98E-04	DUSP1,ELK1,ND3,PER1,PTGS2,SLC1A3,USP2
GO Biological Processes	GO:0045444	fat cell differentiation	4.09E-04	BMP2,NR4A1,PTGS2,ZFP36,ZBTB16,NR4A3
GO Biological Processes	GO:0042310	vasoconstriction	4.23E-04	ADRA1B,ADRA2A,KCNA5,PTGS2
GO Biological Processes	GO:0007188	adenylate cyclase-modulating G protein-coupled receptor signaling pathway	4.38E-04	ADCY5,ADRA1B,ADRA2A,PTGER2,RGS1,ADGRG2
GO Biological Processes	GO:0010837	regulation of keratinocyte proliferation	4.52E-04	KLF9,HAS2,ZFP36

GO Biological Processes	GO:2000117	negative regulation of cysteine-type endopeptidase activity	4.62E-04	NR4A1,PTGS2,THBS1,VEGFA
GO Biological Processes	GO:0006873	cellular ion homeostasis	4.78E-04	ADCY5,ADRA1B,CP,KCNA5,MT2A,PTGER2,SLC1A3,P2RX2,SLAMF8, ATP13A4
GO Biological Processes	GO:0052548	regulation of endopeptidase activity	4.78E-04	CCN2,F3,NR4A1,CCN1,PTGS2,THBS1,VEGFA,PAPLN
GO Biological Processes	GO:0051403	stress-activated MAPK cascade	5.12E-04	BMP2,CCN2,DUSP1,PER1,VEGFA,ZFP36
GO Biological Processes	GO:0032496	response to lipopolysaccharide	5.80E-04	CPS1,CSF2RB,CXCL1,NR4A1,JUN,PTGS2,ZFP36
GO Biological Processes	GO:0031098	stress-activated protein kinase signaling cascade	6.08E-04	BMP2,CCN2,DUSP1,PER1,VEGFA,ZFP36
GO Biological Processes	GO:0006936	muscle contraction	6.32E-04	ADRA1B,ADRA2A,CCN2,NR4A1,KCNA5,PTGS2,P2RX2
GO Biological Processes	GO:1901699	cellular response to nitrogen compound	6.34E-04	ADCY5,CPS1,GH2,NR4A1,PDK4,PTGS2,SLC1A3,NR4A3,KLF15, AMIGO1
GO Biological Processes	GO:0002042	cell migration involved in sprouting angiogenesis	6.47E-04	NR4A1,PTGS2,THBS1,VEGFA
GO Biological Processes	GO:1901655	cellular response to ketone	6.47E-04	ADCY5,KLF9,ELK1,PTGER2
GO Biological Processes	GO:0051591	response to cAMP	6.47E-04	CPS1,DUSP1,JUN,PER1

GO Biological Processes	GO:0090257	regulation of muscle system process	6.61E-04	ADRA1B,ADRA2A,CCN2,NR4A1,PTGS2,NR4A3
GO Biological Processes	GO:1903524	positive regulation of blood circulation	6.64E-04	ADRA1B,CCN2,PTGER2
GO Biological Processes	GO:0033574	response to testosterone	7.13E-04	DUSP1,ELK1,THBS1
GO Biological Processes	GO:0006937	regulation of muscle contraction	7.15E-04	ADRA1B,ADRA2A,CCN2,NR4A1,PTGS2
GO Biological Processes	GO:1903522	regulation of blood circulation	7.32E-04	ADRA1B,ADRA2A,CCN2,KCNA5,PTGER2,PTGS2
GO Biological Processes	GO:0006875	cellular metal ion homeostasis	7.40E-04	ADCY5,ADRA1B,CP,KCNA5,MT2A,PTGER2,SLC1A3,P2RX2,ATP13A4
GO Biological Processes	GO:0002237	response to molecule of bacterial origin	8.10E-04	CPS1,CSF2RB,CXCL1,NR4A1,JUN,PTGS2,ZFP36
GO Biological Processes	GO:0031667	response to nutrient levels	8.14E-04	AQP3,CPS1,GH2,JUN,CYTB,PDK4,PTGS2,ZFP36
GO Biological Processes	GO:0031589	cell-substrate adhesion	8.37E-04	ANGPT2,CCN2,HAS2,CCN1,THBS1,VEGFA,SNED1
GO Biological Processes	GO:0043534	blood vessel endothelial cell migration	8.80E-04	ANGPT2,NR4A1,PTGS2,THBS1,VEGFA
GO Biological Processes	GO:0048661	positive regulation of smooth muscle cell proliferation	9.45E-04	JUN,PTGS2,THBS1,NR4A3

GO Biological Processes	GO:0048660	regulation of smooth muscle cell proliferation	9.49E-04	BMP2,JUN,PTGS2,THBS1,NR4A3
GO Biological Processes	GO:0001934	positive regulation of protein phosphorylation	9.73E-04	ADRA2A,BMP2,CCN2,GH2,CCN1,OSM,PTGS2,THBS1,VEGFA,FGD2
GO Biological Processes	GO:0043616	keratinocyte proliferation	9.92E-04	KLF9,HAS2,ZFP36
GO Biological Processes	GO:0006935	chemotaxis	9.94E-04	ANGPT2,DUSP1,F3,CXCL1,NR4A1,CCN1,THBS1,VEGFA,SLAMF8
GO Biological Processes	GO:0042330	taxis	1.03E-03	ANGPT2,DUSP1,F3,CXCL1,NR4A1,CCN1,THBS1,VEGFA,SLAMF8
GO Biological Processes	GO:0048659	smooth muscle cell proliferation	1.05E-03	BMP2,JUN,PTGS2,THBS1,NR4A3
GO Biological Processes	GO:0001936	regulation of endothelial cell proliferation	1.07E-03	BMP2,F3,NR4A1,THBS1,VEGFA
GO Biological Processes	GO:0050878	regulation of body fluid levels	1.11E-03	ADRA2A,AQP3,CLDN4,F3,HAS2,THBS1,VEGFA
GO Biological Processes	GO:0045778	positive regulation of ossification	1.12E-03	BMP2,CCN1,ZBTB16
GO Biological Processes	GO:0030155	regulation of cell adhesion	1.18E-03	ANGPT2,BMP2,DUSP1,HAS2,IBSP,CCN1,THBS1,VEGFA,ZBTB16,NR4A3
GO Biological Processes	GO:0030162	regulation of proteolysis	1.19E-03	ADRA2A,CLDN4,CCN2,F3,NR4A1,CCN1,PTGS2,THBS1,VEGFA,PAPLN

GO Biological Processes	GO:0001938	positive regulation of endothelial cell proliferation	1.20E-03	BMP2,F3,NR4A1,VEGFA
GO Biological Processes	GO:0002526	acute inflammatory response	1.24E-03	F3,OSM,PTGS2,CD163
GO Biological Processes	GO:0007565	female pregnancy	1.29E-03	ANGPT2,CLDN4,PSG9,PTGS2,VEGFA
GO Biological Processes	GO:0032872	regulation of stress-activated MAPK cascade	1.32E-03	BMP2,CCN2,DUSP1,PER1,VEGFA
GO Biological Processes	GO:0043406	positive regulation of MAP kinase activity	1.33E-03	ADRA2A,THBS1,VEGFA,FGD2
GO Biological Processes	GO:0048871	multicellular organismal homeostasis	1.35E-03	AQP3,CLDN4,CCN2,HAS2,PDK4,PTGS2,VEGFA,NR4A3
GO Biological Processes	GO:0070302	regulation of stress-activated protein kinase signaling cascade	1.42E-03	BMP2,CCN2,DUSP1,PER1,VEGFA
GO Biological Processes	GO:0042493	response to drug	1.42E-03	CPS1,JUN,CYTB,PTGS2,SLC1A3,THBS1,SLAMF8
GO Biological Processes	GO:0001822	kidney development	1.46E-03	ANGPT2,BMP2,HAS2,VEGFA,ZBTB16,KLF15
GO Biological Processes	GO:0001935	endothelial cell proliferation	1.51E-03	BMP2,F3,NR4A1,THBS1,VEGFA
GO Biological Processes	GO:0030282	bone mineralization	1.56E-03	BMP2,IBSP,CCN1,PTGS2

GO Biological Processes	GO:0071385	cellular response to glucocorticoid stimulus	1.65E-03	KLF9,ND3,ZFP36
GO Biological Processes	GO:0048008	platelet-derived growth factor receptor signaling pathway	1.65E-03	F3,VEGFA,NR4A3
GO Biological Processes	GO:0071383	cellular response to steroid hormone stimulus	1.65E-03	KLF9,ND3,PER1,ZFP36,NR4A3
GO Biological Processes	GO:0071902	positive regulation of protein serine/threonine kinase activity	1.69E-03	ADRA2A,BMP2,THBS1,VEGFA,FGD2
GO Biological Processes	GO:0072001	renal system development	1.70E-03	ANGPT2,BMP2,HAS2,VEGFA,ZBTB16,KLF15
GO Biological Processes	GO:0050891	multicellular organismal water homeostasis	1.74E-03	AQP3,CLDN4,HAS2
GO Biological Processes	GO:0055065	metal ion homeostasis	1.75E-03	ADCY5,ADRA1B,CP,KCNA5,MT2A,PTGER2,SLC1A3,P2RX2,ATP13A4
GO Biological Processes	GO:0002688	regulation of leukocyte chemotaxis	1.76E-03	DUSP1,THBS1,VEGFA,SLAMF8
GO Biological Processes	GO:0080135	regulation of cellular response to stress	1.82E-03	BMP2,CCN2,DUSP1,PER1,PTGS2,VEGFA,NR4A3,OTUB2,ARMT1
GO Biological Processes	GO:0043065	positive regulation of apoptotic process	1.89E-03	BMP2,DUSP1,NR4A1,CCN1,JUN,PTGS2,THBS1,ZBTB16
GO Biological Processes	GO:0060326	cell chemotaxis	1.97E-03	DUSP1,CXCL1,NR4A1,THBS1,VEGFA,SLAMF8

GO Biological Processes	GO:0009749	response to glucose	1.99E-03	ADCY5,ADRA2A,ANGPT2,CCN2,THBS1
GO Biological Processes	GO:0042060	wound healing	2.00E-03	ADRA2A,CLDN4,F3,CCN1,PPL,THBS1,VEGFA
GO Biological Processes	GO:0071384	cellular response to corticosteroid stimulus	2.11E-03	KLF9,ND3,ZFP36
GO Biological Processes	GO:0043068	positive regulation of programmed cell death	2.26E-03	BMP2,DUSP1,NR4A1,CCN1,JUN,PTGS2,THBS1,ZBTB16
GO Biological Processes	GO:0044706	multi-multicellular organism process	2.29E-03	ANGPT2,CLDN4,PSG9,PTGS2,VEGFA
GO Biological Processes	GO:0010565	regulation of cellular ketone metabolic process	2.34E-03	BMP2,PDK4,PTGS2,NR4A3
GO Biological Processes	GO:0010810	regulation of cell-substrate adhesion	2.34E-03	ANGPT2,HAS2,CCN1,THBS1,VEGFA
GO Biological Processes	GO:0061041	regulation of wound healing	2.40E-03	ADRA2A,CLDN4,F3,THBS1
GO Biological Processes	GO:0030104	water homeostasis	2.42E-03	AQP3,CLDN4,HAS2
GO Biological Processes	GO:0097529	myeloid leukocyte migration	2.43E-03	DUSP1,CXCL1,THBS1,VEGFA,SLAMF8
GO Biological Processes	GO:0001101	response to acid chemical	2.46E-03	CPS1,CCN2,VEGFA,AMIGO1

GO Biological Processes	GO:0003170	heart valve development	2.53E-03	BMP2,CCN1,MATR3
GO Biological Processes	GO:0019216	regulation of lipid metabolic process	2.57E-03	ADRA2A,BMP2,CCN1,PDK4,PTGS2,NR4A3
GO Biological Processes	GO:0050918	positive chemotaxis	2.64E-03	ANGPT2,F3,VEGFA
GO Biological Processes	GO:0045598	regulation of fat cell differentiation	2.74E-03	BMP2,PTGS2,ZFP36,ZBTB16
GO Biological Processes	GO:0045333	cellular respiration	2.78E-03	ATP8,CYTB,ND2,ND3,NR4A3
GO Biological Processes	GO:0006979	response to oxidative stress	2.79E-03	DUSP1,JUN,KCNA5,ND3,PTGS2,NR4A3,DHRS2
GO Biological Processes	GO:0046686	response to cadmium ion	2.88E-03	JUN,MT2A,CYTB
GO Biological Processes	GO:0006119	oxidative phosphorylation	2.88E-03	ATP8,CYTB,ND2,ND3
GO Biological Processes	GO:0030595	leukocyte chemotaxis	2.88E-03	DUSP1,CXCL1,THBS1,VEGFA,SLAMF8
GO Biological Processes	GO:0001655	urogenital system development	2.98E-03	ANGPT2,BMP2,HAS2,VEGFA,ZBTB16,KLF15
GO Biological Processes	GO:0007610	behavior	2.99E-03	ADCY5,JUN,PTGS2,SLC1A3,THBS1,NR4A3,USP2,P2RX2

GO Biological Processes	GO:0044057	regulation of system process	2.99E-03	ADRA1B,ADRA2A,CCN2,NR4A1,KCNA5,PTGER2,PTGS2,NR4A3
GO Biological Processes	GO:0042531	positive regulation of tyrosine phosphorylation of STAT protein	3.00E-03	GH2,OSM,VEGFA
GO Biological Processes	GO:0072006	nephron development	3.03E-03	ANGPT2,BMP2,VEGFA,KLF15
GO Biological Processes	GO:0042542	response to hydrogen peroxide	3.27E-03	DUSP1,JUN,KCNA5,NR4A3
GO Biological Processes	GO:0072593	reactive oxygen species metabolic process	3.27E-03	CCN2,CCN1,ND2,PDK4,THBS1
GO Biological Processes	GO:0033555	multicellular organismal response to stress	3.38E-03	ADRA2A,THBS1,P2RX2
GO Biological Processes	GO:0051592	response to calcium ion	3.60E-03	AQP3,DUSP1,CYTB,THBS1
GO Biological Processes	GO:0055067	monovalent inorganic cation homeostasis	3.69E-03	KCNA5,PDK4,SLC1A3,SLAMF8
GO Biological Processes	GO:0043535	regulation of blood vessel endothelial cell migration	3.77E-03	ANGPT2,PTGS2,THBS1,VEGFA
GO Biological Processes	GO:0033002	muscle cell proliferation	3.83E-03	BMP2,JUN,PTGS2,THBS1,NR4A3
GO Biological Processes	GO:0010631	epithelial cell migration	3.94E-03	ANGPT2,HAS2,NR4A1,PTGS2,THBS1,VEGFA

GO Biological Processes	GO:0003007	heart morphogenesis	4.03E-03	BMP2,HAS2,CCN1,VEGFA,ASB2
GO Biological Processes	GO:0043536	positive regulation of blood vessel endothelial cell migration	4.09E-03	PTGS2,THBS1,VEGFA
GO Biological Processes	GO:0010951	negative regulation of endopeptidase activity	4.10E-03	NR4A1,PTGS2,THBS1,VEGFA,PAPLN
GO Biological Processes	GO:0090132	epithelium migration	4.10E-03	ANGPT2,HAS2,NR4A1,PTGS2,THBS1,VEGFA
GO Biological Processes	GO:1901653	cellular response to peptide	4.15E-03	CPS1,GH2,NR4A1,PDK4,NR4A3,KLF15
GO Biological Processes	GO:0007193	adenylate cyclase-inhibiting G protein-coupled receptor signaling pathway	4.24E-03	ADCY5,ADRA2A,RGS1
GO Biological Processes	GO:0090049	regulation of cell migration involved in sprouting angiogenesis	4.24E-03	PTGS2,THBS1,VEGFA
GO Biological Processes	GO:0072503	cellular divalent inorganic cation homeostasis	4.30E-03	ADCY5,ADRA1B,KCNA5,MT2A,PTGER2,P2RX2,ATP13A4
GO Biological Processes	GO:0071900	regulation of protein serine/threonine kinase activity	4.32E-03	ADRA2A,BMP2,DUSP1,THBS1,VEGFA,FGD2
GO Biological Processes	GO:0090130	tissue migration	4.38E-03	ANGPT2,HAS2,NR4A1,PTGS2,THBS1,VEGFA
GO Biological Processes	GO:0045927	positive regulation of growth	4.60E-03	DIO3,FOXS1,GH2,KRT17,VEGFA

GO Biological Processes	GO:0050900	leukocyte migration	4.68E-03	DUSP1,CXCL1,THBS1,VEGFA,ASB2,SLAMF8
GO Biological Processes	GO:0046209	nitric oxide metabolic process	4.71E-03	CPS1,PTGS2,PTX3
GO Biological Processes	GO:0010466	negative regulation of peptidase activity	4.83E-03	NR4A1,PTGS2,THBS1,VEGFA,PAPLN
GO Biological Processes	GO:0009636	response to toxic substance	4.83E-03	CPS1,MT2A,CYTB,PTGS2,DHRS2
GO Biological Processes	GO:2001057	reactive nitrogen species metabolic process	4.88E-03	CPS1,PTGS2,PTX3
GO Biological Processes	GO:0051346	negative regulation of hydrolase activity	5.11E-03	NR4A1,PTGS2,PTX3,THBS1,VEGFA,PAPLN
GO Biological Processes	GO:1903034	regulation of response to wounding	5.26E-03	ADRA2A,CLDN4,F3,THBS1
GO Biological Processes	GO:0008285	negative regulation of cell population proliferation	5.27E-03	BMP2,KLF9,CXCL1,OSM,PTGS2,THBS1,ZBTB16,DHRS2,P3H2
GO Biological Processes	GO:0006970	response to osmotic stress	5.39E-03	TSC22D3,CYTB,PTGS2
GO Biological Processes	GO:0072507	divalent inorganic cation homeostasis	5.39E-03	ADCY5,ADRA1B,KCNA5,MT2A,PTGER2,P2RX2,ATP13A4
GO Biological Processes	GO:0031214	biomineral tissue development	5.49E-03	BMP2,IBSP,CCN1,PTGS2

GO Biological Processes	GO:0042509	regulation of tyrosine phosphorylation of STAT protein	5.57E-03	GH2,OSM,VEGFA
GO Biological Processes	GO:0110148	biomineralization	5.72E-03	BMP2,IBSP,CCN1,PTGS2
GO Biological Processes	GO:0046890	regulation of lipid biosynthetic process	5.72E-03	BMP2,CCN1,PDK4,PTGS2
GO Biological Processes	GO:0043086	negative regulation of catalytic activity	5.72E-03	BMP2,DUSP1,NR4A1,PTGS2,PTX3,THBS1,VEGFA,ZFP36,PAPLN
GO Biological Processes	GO:0019646	aerobic electron transport chain	5.75E-03	CYTB,ND2,ND3
GO Biological Processes	GO:0051781	positive regulation of cell division	6.12E-03	OSM,VEGFA,PROK1
GO Biological Processes	GO:0007260	tyrosine phosphorylation of STAT protein	6.12E-03	GH2,OSM,VEGFA
GO Biological Processes	GO:0022900	electron transport chain	6.20E-03	CYTB,ND2,ND3,DHRS2
GO Biological Processes	GO:0010634	positive regulation of epithelial cell migration	6.45E-03	HAS2,PTGS2,THBS1,VEGFA
GO Biological Processes	GO:0043542	endothelial cell migration	6.46E-03	ANGPT2,NR4A1,PTGS2,THBS1,VEGFA
GO Biological Processes	GO:0033273	response to vitamin	6.51E-03	AQP3,CYTB,PTGS2

GO Biological Processes	GO:0045860	positive regulation of protein kinase activity	6.53E-03	ADRA2A,BMP2,CCN1,THBS1,VEGFA,FGD2
GO Biological Processes	GO:0009266	response to temperature stimulus	6.70E-03	CYTB,PTGS2,THBS1,ANO3
GO Biological Processes	GO:0006941	striated muscle contraction	6.70E-03	ADRA1B,CCN2,NR4A1,KCNA5
GO Biological Processes	GO:0097306	cellular response to alcohol	6.71E-03	ADCY5,KLF9,PTGER2
GO Biological Processes	GO:0019217	regulation of fatty acid metabolic process	6.91E-03	PDK4,PTGS2,NR4A3
GO Biological Processes	GO:0045766	positive regulation of angiogenesis	6.97E-03	ANGPT2,F3,THBS1,VEGFA
GO Biological Processes	GO:1904018	positive regulation of vasculature development	6.97E-03	ANGPT2,F3,THBS1,VEGFA
GO Biological Processes	GO:0010959	regulation of metal ion transport	7.08E-03	ADRA2A,KCNA5,PER1,PTGS2,P2RX2,AMIGO1
GO Biological Processes	GO:0042773	ATP synthesis coupled electron transport	7.33E-03	CYTB,ND2,ND3
GO Biological Processes	GO:0042775	mitochondrial ATP synthesis coupled electron transport	7.33E-03	CYTB,ND2,ND3
GO Biological Processes	GO:0006942	regulation of striated muscle contraction	7.33E-03	ADRA1B,CCN2,NR4A1

GO Biological Processes	GO:0001503	ossification	7.50E-03	BMP2,CCN2,IBSP,CCN1,PTGS2,ZBTB16
GO Biological Processes	GO:0001823	mesonephros development	7.54E-03	BMP2,VEGFA,ZBTB16
GO Biological Processes	GO:0071375	cellular response to peptide hormone stimulus	7.56E-03	CPS1,GH2,NR4A1,PDK4,NR4A3
GO Biological Processes	GO:0010632	regulation of epithelial cell migration	7.67E-03	ANGPT2,HAS2,PTGS2,THBS1,VEGFA
GO Biological Processes	GO:0002040	sprouting angiogenesis	7.94E-03	NR4A1,PTGS2,THBS1,VEGFA
GO Biological Processes	GO:0009060	aerobic respiration	8.09E-03	ATP8,CYTB,ND2,ND3
GO Biological Processes	GO:0051216	cartilage development	8.39E-03	BMP2,CCN2,CCN1,ZBTB16
GO Biological Processes	GO:0050731	positive regulation of peptidyl-tyrosine phosphorylation	8.69E-03	ADRA2A,GH2,OSM,VEGFA
GO Biological Processes	GO:0007162	negative regulation of cell adhesion	8.79E-03	ANGPT2,BMP2,DUSP1,THBS1,VEGFA
GO Biological Processes	GO:0032103	positive regulation of response to external stimulus	8.96E-03	F3,OSM,PTGS2,THBS1,VEGFA,MATR3
GO Biological Processes	GO:0071674	mononuclear cell migration	9.16E-03	DUSP1,THBS1,ASB2,SLAMF8

GO Biological Processes	GO:0042594	response to starvation	9.32E-03	CPS1,JUN,PDK4,ZFP36
GO Biological Processes	GO:0010952	positive regulation of peptidase activity	9.81E-03	CLDN4,CCN2,F3,CCN1
GO Biological Processes	GO:0045785	positive regulation of cell adhesion	9.97E-03	HAS2,IBSP,CCN1,VEGFA,ZBTB16,NR4A3
GO Cellular Components	GO:0044853	plasma membrane raft	9.71E-06	ADRA1B,HAS2,KCNA5,MYO1A,PTGS2,CAVIN2,ADRA2A,CCN2,NR4A1,NR4A3,P2RX2,KLF15,CPS1,SLC1A3,VEGFA,PTGER2,MATR3,DHRS2, AGPAT5,PER1,AMIGO1
GO Cellular Components	GO:0031012	extracellular matrix	1.37E-04	ANGPT2,CCN2,F3,CCN1,LGALS3BP,PTX3,THBS1,VEGFA,P3H2,PAPLN
GO Cellular Components	GO:0030312	external encapsulating structure	1.39E-04	ANGPT2,CCN2,F3,CCN1,LGALS3BP,PTX3,THBS1,VEGFA,P3H2,PAPLN
GO Cellular Components	GO:0005901	caveola	4.42E-04	ADRA1B,KCNA5,PTGS2,CAVIN2
GO Cellular Components	GO:0009925	basal plasma membrane	7.18E-04	ADRA2A,AQP3,CLDN4,MYO1A,SLC1A3,ABCA8
GO Cellular Components	GO:0045178	basal part of cell	1.04E-03	ADRA2A,AQP3,CLDN4,MYO1A,SLC1A3,ABCA8
GO Cellular Components	GO:0062023	collagen-containing extracellular matrix	2.06E-03	ANGPT2,CCN2,F3,CCN1,LGALS3BP,THBS1,P3H2
GO Cellular Components	GO:0005637	nuclear inner membrane	2.11E-03	PTGS2,MATR3,P2RX2

GO Cellular Components	GO:0019866	organelle inner membrane	2.26E-03	CPS1,ATP8,CYTB,ND2,ND3,PTGS2,MATR3,P2RX2
GO Cellular Components	GO:0098857	membrane microdomain	2.89E-03	ADRA1B,HAS2,KCNA5,MYO1A,PTGS2,CAVIN2
GO Cellular Components	GO:0045121	membrane raft	2.89E-03	ADRA1B,HAS2,KCNA5,MYO1A,PTGS2,CAVIN2
GO Cellular Components	GO:0016529	sarcoplasmic reticulum	3.12E-03	THBS1,RASD1,P3H2
GO Cellular Components	GO:0098800	inner mitochondrial membrane protein complex	3.35E-03	ATP8,CYTB,ND2,ND3
GO Cellular Components	GO:0005635	nuclear envelope	3.46E-03	ADRA1B,NR4A1,PTGS2,MATR3,DHRS2,P2RX2,AGPAT5
GO Cellular Components	GO:0016528	sarcoplasm	4.39E-03	THBS1,RASD1,P3H2
GO Cellular Components	GO:0005746	mitochondrial respirasome	6.12E-03	CYTB,ND2,ND3
GO Cellular Components	GO:0098803	respiratory chain complex	6.32E-03	CYTB,ND2,ND3
GO Cellular Components	GO:0031965	nuclear membrane	8.10E-03	ADRA1B,NR4A1,PTGS2,MATR3,P2RX2
GO Cellular Components	GO:0070469	respirasome	8.44E-03	CYTB,ND2,ND3

GO Molecular Functions	GO:0008083	growth factor activity	5.34E-06	BMP2,CCN2,GH2,CXCL1,OSM,VEGFA,PROK1,ANGPT2,NR4A1,IBSP, THBS1,CSF2RB,DIO3,FOXS1,KRT17,ADRA2A
GO Molecular Functions	GO:0001968	fibronectin binding	2.12E-04	CCN2,THBS1,VEGFA
GO Molecular Functions	GO:0015399	primary active transmembrane transporter activity	9.73E-04	CYTB,ND2,ND3,ABCA8,ATP13A4
GO Molecular Functions	GO:0022804	active transmembrane transporter activity	1.59E-03	CYTB,ND2,ND3,SLC1A3,ABCA8,SLC35E1,ATP13A4
GO Molecular Functions	GO:0050840	extracellular matrix binding	1.65E-03	CCN1,THBS1,VEGFA
GO Molecular Functions	GO:0019838	growth factor binding	2.96E-03	CCN2,DUSP1,CCN1,THBS1
GO Molecular Functions	GO:0005178	integrin binding	3.11E-03	CCN2,IBSP,CCN1,THBS1
GO Molecular Functions	GO:0015453	oxidoreduction-driven active transmembrane transporter activity	3.38E-03	CYTB,ND2,ND3
GO Molecular Functions	GO:0015318	inorganic molecular entity transmembrane transporter activity	3.75E-03	AQP3,CLDN4,KCNA5,ATP8,CYTB,SLC1A3,P2RX2,ANO3,ATP13A4
GO Molecular Functions	GO:0016491	oxidoreductase activity	3.92E-03	CP,DIO3,CYTB,ND2,ND3,PTGS2,DHRS2,P3H2,CBSL
GO Molecular Functions	GO:0048018	receptor ligand activity	4.25E-03	BMP2,CCN2,GH2,CXCL1,OSM,VEGFA,PROK1

GO Molecular Functions	GO:0030546	signaling receptor activator activity	4.64E-03	BMP2,CCN2,GH2,CXCL1,OSM,VEGFA,PROK1
GO Molecular Functions	GO:0008201	heparin binding	5.15E-03	CCN2,CCN1,THBS1,VEGFA
GO Molecular Functions	GO:1901681	sulfur compound binding	5.22E-03	CCN2,CCN1,THBS1,VEGFA,CBSL
GO Molecular Functions	GO:0030545	signaling receptor regulator activity	7.59E-03	BMP2,CCN2,GH2,CXCL1,OSM,VEGFA,PROK1
KEGG Pathway	hsa05012	Parkinson's disease	3.24E-04	ADCY5,ATP8,CYTB,ND2,ND3,PTGS2
KEGG Pathway	ko04151	PI3K-Akt signaling pathway	5.80E-04	ANGPT2,GH2,NR4A1,IBSP,OSM,THBS1,VEGFA,ELK1,JUN
KEGG Pathway	hsa04060	Cytokine-cytokine receptor interaction	9.46E-04	BMP2,CSF2RB,GH2,CXCL1,OSM,VEGFA
KEGG Pathway	hsa05200	Pathways in cancer	1.34E-03	ADCY5,BMP2,JUN,PTGER2,PTGS2,VEGFA,ZBTB16,FKBP5,KRT17,ELK1,CXCL1
KEGG Pathway	hsa04510	Focal adhesion	1.48E-03	ELK1,IBSP,JUN,THBS1,VEGFA
KEGG Pathway	hsa04066	HIF-1 signaling pathway	1.56E-03	ANGPT2,F3,KCNA5,VEGFA,JUN
KEGG Pathway	hsa00190	Oxidative phosphorylation	2.34E-03	ATP8,CYTB,ND2,ND3

KEGG Pathway	hsa04915	Estrogen signaling pathway	3.19E-03	ADCY5,FKBP5,JUN,KRT17
KEGG Pathway	hsa05140	Leishmania infection	3.52E-03	ELK1,JUN,PTGS2
KEGG Pathway	ko05140	Leishmaniasis	3.52E-03	ELK1,JUN,PTGS2
KEGG Pathway	hsa04921	Oxytocin signaling pathway	4.63E-03	ADCY5,ELK1,JUN,PTGS2
KEGG Pathway	hsa04723	Retrograde endocannabinoid signaling	5.26E-03	ADCY5,ND2,ND3,PTGS2
KEGG Pathway	hsa04080	Neuroactive ligand-receptor interaction	6.09E-03	ADRA1B,ADRA2A,GH2,PTGER2,P2RX2
KEGG Pathway	ko05323	Rheumatoid arthritis	6.32E-03	CXCL1,JUN,VEGFA
KEGG Pathway	hsa04912	GnRH signaling pathway	6.71E-03	ADCY5,ELK1,JUN
KEGG Pathway	ko04657	IL-17 signaling pathway	6.91E-03	CXCL1,JUN,PTGS2
KEGG Pathway	ko04933	AGE-RAGE signaling pathway in diabetic complications	8.21E-03	F3,JUN,VEGFA
KEGG Pathway	hsa04713	circadian entrainment	8.44E-03	ADCY5,PER1,RASD1

TABLE S6. SLE dysregulated biological functions in male placentas

Category	Term	Description	*P* value	Symbols
GO Biological Processes	GO:0001819	positive regulation of cytokine production	1.70E-15	ALOX15B,C3,TNFRSF8,CLU,EGR1,F2R,HLA-DPA1,HLA-G,IL1B,IL15, IRF7,LUM,MMP12,NOS2,OAS1,OAS2,OAS3,OSM,PAEP,SERPINE1, PTGS2,STAT1,THBS1,NR4A3,SPHK1,IL1RL1,CD83,IL27RA,ISG15,IL17D, INAVA,SULF2,IFIH1,CD276,RSAD2,NLRP3,CLNK,FN1,HLA-DRB1, INHA,INHBB,ITGB6,ZFP36,IL1R2,PDCD1LG2,SLC2A10
GO Biological Processes	GO:0044706	multi-multicellular organism process	2.27E-14	ADRA2C,ARHGDIB,AVPR1A,PRDM1,CNR1,FOS,FOSB,IGFBP2,IL1B, OXTR,SERPINE2,PRL,PTGS2,PTHLH,RGS2,SPP1,TAC3,TIMP1,VEGFA, FOSL1,CITED2,CORIN,WNT4,RXFP1,TLE6
GO Biological Processes	GO:0030155	regulation of cell adhesion	4.03E-14	ARHGDIB,CD9,CDSN,EGR3,PTK2B,FN1,HLA-DPA1,HLA-DQA1,HLA-DQB1,HLA-DRA,HLA-DRB1,HLA-G, TNC,IGFBP2,CCN1,IL1B,CXCL8, IL15,LAMA3,LGALS3,MELTF,MMP12,SERPINE1,SERPINE2,PLAUR, PTPRR,SFRP1,SPOCK1,THBS1,VEGFA,ZBTB16,NR4A3,TNFSF18, SEMA5A,CD83,CHST2,IL27RA,NUAK1,FSTL3,CITED2,ADAMDEC1, WNT4,PDCD1LG2,CD276,NLRP3,PRDM1,CD22,CNR1,CCN2,INHA, SPHK1,IL1RL1,CLNK,GCNT1,PODXL2,EGR1,MAFB,RSAD2,ULBP1, ISG15,CLU
GO Biological Processes	GO:0001817	regulation of cytokine production	8.69E-14	ALOX15B,C3,TNFRSF8,CLU,EGR1,F2R,FN1,HLA-DPA1,HLA-DRB1,HLA-G,IL1B,IL15,INHA,INHBB,IRF7,ITGB6,LUM,MMP12,NOS2,OAS1,OAS2,OAS3,OSM,PAEP,SERPINE1,PTGS2,STAT1,THBS1,ZFP36,IL1R2,NR4A3,SPHK1,IL1RL1,CD83,IL27RA,ISG15,IL17D,INAVA,SULF2,IFIH1, PDCD1LG2,CD276,SLC2A10,RSAD2,NLRP3,CLNK
GO Biological Processes	GO:0001568	blood vessel development	1.02E-13	AQP1,ADGRB3,PRDM1,C3,CDH2,COMP,CCN2,GADD45A,EGR1,EGR3,PTK2B,FLT1,FN1,HK2,HLA-G,NR4A1,HSPG2,CCN1,IL1B,CXCL8,JUN, LOX,MMP19,SERPINE1,PRL,PTGS2,SFRP1,STAT1,TCF7L2,THBS1, VEGFA,WT1,FOSL1,ALDH1A2,SPHK1,SEMA5A,NOG,RAMP1,CITED2, TIPARP,WNT4,STAB2,SLC2A10,PROK1,HSPB6

GO Biological Processes	GO:0048525	negative regulation of viral process	1.08E-13	IFIT1,LY6E,CIITA,MX1,OAS1,OAS2,OAS3,PTX3,SLPI,STAT1,ZFP36,IFITM1,OASL,ISG15,TRIM14,IFIH1,RSAD2,HLA-DRB1,CXCL8,IL27RA,IFI27,IRF7,MMP12,USP18,ITGB6,JUN,NCAM1,CXCR6,CLEC5A,PARP9,LRRC15,CLU,IFIT3,IL15,MX2,FOSL1,IFI44,IFI44L,DDIT4,FOS,IL1B,IL2RB,TNFSF10,CLDN19,CXCL3,NLRP3,TNFRSF8,THBS1,INAVA,IFI35
GO Biological Processes	GO:0007565	female pregnancy	7.88E-13	ADRA2C,ARHGDIB,PRDM1,CNR1,FOS,FOSB,IGFBP2,IL1B,OXTR,PRL,PTGS2,PTHLH,RGS2,SPP1,TAC3,TIMP1,VEGFA,FOSL1,CITED2,CORIN,WNT4,TLE6
GO Biological Processes	GO:0051346	negative regulation of hydrolase activity	8.81E-13	SERPINA3,AQP1,SERPING1,C3,CST6,SFN,NR4A1,ITIH3,ANOS1,LGALS3,SERPINE1,SERPINB5,SERPINE2,PLAUR,PTGS2,PTX3,RGS2,SLPI,SPOCK1,THBS1,TIMP1,TIMP3,VEGFA,SLC39A14,PCSK1N,GMIP,PPP4R4,BIRC7,A2ML1,ANXA8L1,CNR1,GADD45A,GRM7,IFIT1,IL1B,RPS15,SFRP1,UCHL1,ZFP36,GPRC5A,SH3BP5,MYOZ1,DEPTOR,PARP9,CCN2,F2R,CCN1,TNFSF10,EGLN3,NLRP3,FN1,GAS1,IL1R2,CLU,PTK2B,MELTF,WNT10B,TRIM67,CD9,ISLR,QSOX1,LEFTY2,AOC1,ALDOC,LGALS3BP,TTR,QPCT,PLAC8,BIN2
GO Biological Processes	GO:0043086	negative regulation of catalytic activity	9.20E-13	SERPINA3,AQP1,SERPING1,C3,CNR1,CST6,GADD45A,SFN,GRM7,NR4A1,IFIT1,IL1B,ITIH3,ANOS1,LGALS3,SERPINE1,SERPINB5,SERPINE2,PLAUR,PTGS2,PTX3,RGS2,RPS15,SFRP1,SLPI,SPOCK1,THBS1,TIMP1,TIMP3,UCHL1,VEGFA,ZFP36,GPRC5A,SH3BP5,SLC39A14,PCSK1N,GMIP,PPP4R4,MYOZ1,DEPTOR,BIRC7,PARP9,A2ML1,ANXA8L1
GO Biological Processes	GO:0009612	response to mechanical stimulus	1.00E-12	AQP1,TNFRSF8,CDH2,GADD45A,ENDOG,PTK2B,FOS,FOSB,TNC,IGFBP2,IL1B,JUN,MAG,SERPINE2,PTGS2,SLC1A3,STAT1,THBS1,FOSL1,TRPA1,CITED2,ANKRD1,PDZD7,AOC1,ADCY1,AVPR1A,CD9,FOXO1,KCNH1,KRT14,MAOB,MT1X,PHEX,PYCR1,SOD2,SOD3,NR4A3,SPHK1,PTGES,FSTL3,CPNE7,SYT8,LOX,NOS2,OXTR,SFRP1,SLC6A11,UCHL1
GO Biological Processes	GO:0019221	cytokine-mediated signaling pathway	1.93E-12	CCR1,EGR1,PTK2B,XCR1,CXCL3,IFI27,IL1B,IL2RB,CXCL8,IL15,IRF7,MMP12,MX1,OAS1,OAS2,OAS3,OSM,PTPRN,CCL18,STAT1,IL1R2,IFITM1,OASL,SPHK1,TNFSF18,IL1RL1,CRLF1,IL27RA,ISG15,CXCR6,USP18,PARP9,IL17RE,FPR3,HLA-DPA1,HLA-DQA1,HLA-DQB1,HLA-DRA,HLA-DRB1,COMP,THBS1,ZFP36,NOG,ADCY1,AQP1,AVPR1A,CD22,NOS2,RGS2,VEGFA,RASD1,MYOZ1,LOX

GO Biological Processes	GO:0008285	negative regulation of cell population prolif	2.38E-12	ADORA3,ALOX15B,ASCL2,KLF9,CD9,TNFRSF8,F2R,PTK2B,FLT1,SFN, HLA-DRB1,HLA-G,IFI35,IFIT3,IGFBP6,IL1B,CXCL8,IL15,ROR2,OSM, SERPINE2,PRL,PTGS2,PTHLH,RBP4,SFRP1,SOD2,STAT1,THBS1, WNT10B,WNT9A,WT1,ZBTB16,FOSL1,IFITM1,ALDH1A2,NOG,PTGES,CITED2,LDOC1,PDCD1LG2,ATOH8,CLDN19
GO Biological Processes	GO:0010951	negative regulation of endopeptidase activit	3.62E-12	SERPINA3,AQP1,SERPING1,C3,CST6,SFN,NR4A1,ITIH3,ANOS1, SERPINE1,SERPINB5,SERPINE2,PLAUR,PTGS2,SLPI,SPOCK1,THBS1, TIMP1,TIMP3,VEGFA,PCSK1N,BIRC7,A2ML1,ANXA8L1
GO Biological Processes	GO:0048514	blood vessel morphogenesis	3.66E-12	AQP1,ADGRB3,PRDM1,C3,CDH2,COMP,CCN2,GADD45A,EGR3,PTK2B, FLT1,FN1,HK2,HLA-G,NR4A1,HSPG2,CCN1,IL1B,CXCL8,JUN,LOX, MMP19,SERPINE1,PRL,PTGS2,SFRP1,STAT1,THBS1,VEGFA,WT1,SPHK1,SEMA5A,NOG,RAMP1,CITED2,TIPARP,WNT4,STAB2,PROK1,HSPB6
GO Biological Processes	GO:0010466	negative regulation of peptidase activity	8.30E-12	SERPINA3,AQP1,SERPING1,C3,CST6,SFN,NR4A1,ITIH3,ANOS1,SERPINE1,SERPINB5,SERPINE2,PLAUR,PTGS2,SLPI,SPOCK1,THBS1,TIMP1,TIMP3,VEGFA,PCSK1N,BIRC7,A2ML1,ANXA8L1
GO Biological Processes	GO:0071496	cellular response to external stimulus	1.46E-11	AOC1,AQP1,AVPR1A,TNFRSF8,CD68,GADD45A,FOXO1,FOS,TNC,IL1B, IL15,INHBB,JUN,KCNB1,MAG,NR4A2,PHEX,PTGS2,SFRP1,FOSL1,NUAK1,ANKRD1,WNT4,NUAK2,SIK1,SIK1B,CNR1,CYP26A1,IGFBP2,PRL,SOD2,SPP1,STAT1,ZFP36,ALDH1A2,PCSK1N
GO Biological Processes	GO:0050792	regulation of viral process	2.19E-11	HLA-DRB1,IFIT1,CXCL8,LY6E,CIITA,MX1,OAS1,OAS2,OAS3,PTX3,SLPI, STAT1,ZFP36,IFITM1,OASL,ISG15,TRIM14,IFIH1,RSAD2
GO Biological Processes	GO:0052548	regulation of endopeptidase activity	2.82E-11	SERPINA3,AQP1,SERPING1,C3,CST6,CCN2,F2R,SFN,NR4A1,CCN1,ITIH3,ANOS1,SERPINE1,SERPINB5,SERPINE2,PLAUR,PTGS2,SLPI,SPOCK1, THBS1,TIMP1,TIMP3,VEGFA,TNFSF10,PCSK1N,BIRC7,EGLN3,NLRP3,A2ML1,ANXA8L1
GO Biological Processes	GO:0052547	regulation of peptidase activity	2.92E-11	SERPINA3,AQP1,SERPING1,C3,CST6,CCN2,F2R,FN1,SFN,NR4A1,CCN1,ITIH3,ANOS1,SERPINE1,SERPINB5,SERPINE2,PLAUR,PTGS2,SLPI,SPOCK1,THBS1,TIMP1,TIMP3,VEGFA,TNFSF10,PCSK1N,BIRC7,EGLN3,NLRP3,A2ML1,ANXA8L1

GO Biological Processes	GO:0070848	response to growth factor	4.61E-11	COMP,CCN2,EGR1,EGR3,FLT1,FOS,GALNT3,GAS1,NR4A1,TNC,CCN1,IL1B,CXCL8,ITGB6,JUN,LOX,LUM,ROR2,PCSK6,SFRP1,LEFTY2,THBS1,VEGFA,ZFP36,CILP,SPHK1,NOG,FSTL3,CITED2,ANKRD1,WNT4,DDIT4,SULF2,PMEPA1,SLC2A10,CHRDL1,TRIM71,HTRA4,FAM83G,INHA,INHBB,DKK1,ATOH8
GO Biological Processes	GO:0070106	interleukin-27-mediated signaling pathway	1.14E-10	MX1,OAS1,OAS2,STAT1,OASL,IL27RA
GO Biological Processes	GO:0034340	response to type I interferon	1.16E-10	IFI27,IFIT1,IRF7,MMP12,MX1,OAS1,OAS2,OAS3,STAT1,IFITM1,ISG15,USP18
GO Biological Processes	GO:0045861	negative regulation of proteolysis	1.30E-10	SERPINA3,AQP1,SERPING1,C3,CST6,GAS1,SFN,NR4A1,ITIH3,ANOS1,SERPINE1,SERPINB5,SERPINE2,PLAUR,PTGS2,SLPI,SPOCK1,THBS1,TIMP1,TIMP3,VEGFA,IL1R2,PCSK1N,BIRC7,A2ML1,ANXA8L1
GO Biological Processes	GO:0009617	response to bacterium	1.41E-10	AQP1,C3,CD68,CNR1,F2R,FKBP5,FMO1,FOS,GGT5,CXCL3,HLA-DRB1,NR4A1,IL1B,CXCL8,JUN,MAOB,NOS2,OAS1,OAS2,OAS3,SERPINE1,PRG2,PTGS2,RGS1,SLPI,SOD2,ZFP36,IL27RA,PTGES,ISG15,IFI44,LDOC1,ANKRD1,PLAC8,STAB2,INAVA,PDCD1LG2,NLRP3,CMPK2
GO Biological Processes	GO:0030162	regulation of proteolysis	1.65E-10	SERPINA3,AQP1,SERPING1,C3,CLU,CST6,CCN2,F2R,PTK2B,FN1,GAS1,SFN,NR4A1,CCN1,IL1B,ITIH3,ANOS1,MELTF,SERPINE1,SERPINB5,SERPINE2,PLAUR,PTGS2,SLPI,SPOCK1,THBS1,TIMP1,TIMP3,VEGFA,WNT10B,IL1R2,TNFSF10,PCSK1N,BIRC7,EGLN3,NLRP3,A2ML1,TRIM67,ANXA8L1
GO Biological Processes	GO:0071363	cellular response to growth factor stimulus	1.89E-10	COMP,CCN2,EGR1,EGR3,FLT1,FOS,GALNT3,GAS1,NR4A1,CCN1,IL1B,CXCL8,ITGB6,JUN,LOX,ROR2,PCSK6,SFRP1,LEFTY2,THBS1,VEGFA,ZFP36,CILP,SPHK1,NOG,FSTL3,CITED2,ANKRD1,WNT4,DDIT4,SULF2,PMEPA1,SLC2A10,CHRDL1,TRIM71,HTRA4,FAM83G
GO Biological Processes	GO:0016032	viral process	2.29E-10	HLA-DRB1,IFI27,IFIT1,CXCL8,IRF7,ITGB6,JUN,LY6E,CIITA,MX1,NCAM1,OAS1,OAS2,OAS3,PTX3,SLPI,STAT1,ZFP36,IFITM1,OASL,ISG15,TRIM14,CXCR6,CLEC5A,IFIH1,PARP9,RSAD2,LRRC15
GO Biological Processes	GO:1903900	regulation of viral life cycle	2.86E-10	HLA-DRB1,IFIT1,CXCL8,LY6E,CIITA,MX1,OAS1,OAS2,OAS3,PTX3,SLPI,IFITM1,OASL,ISG15,TRIM14,IFIH1,RSAD2

GO Biological Processes	GO:0022407	regulation of cell-cell adhesion	3.00E-10	CD9,CDSN,EGR3,HLA-DPA1,HLA-DQA1,HLA-DQB1,HLA-DRA,HLA-DRB1,HLA-G,IGFBP2,IL1B,IL15,LGALS3,SERPINE2,PLAUR,PTPRR,VEGFA,ZBTB16,NR4A3,TNFSF18,CD83,CHST2,IL27RA,FSTL3,CITED2,WNT4,PDCD1LG2,CD276,NLRP3
GO Biological Processes	GO:0048608	reproductive structure development	3.92E-10	ALOX15B,ASCL2,PRDM1,C3,TNC,CCN1,INHA,INHBB,ROR2,SERPINB5,SERPINE2,PTGS2,PTPRN,PTX3,RBP4,SFRP1,SPP1,VEGFA,WT1,FOSL1,TNFSF10,NOG,CYP7B1,FSTL3,CITED2,TIPARP,WNT4,GREB1L,CYP26A1,EGR1,GCNT1,STAT1,ZBTB16,ALDH1A2,CRLF1,SULF2,LY6E,ARHGDIB,CNR1,OXTR,PAEP,TIMP1,CDH2,MYCN,CELSR1,MICAL2,TRIM71,DKK1
GO Biological Processes	GO:1903706	regulation of hemopoiesis	4.06E-10	PRDM1,CCR1,EGR3,PTK2B,FOS,HLA-DRA,HLA-DRB1,HLA-G,IL15,INHA,IRF7,JUN,LGALS3,LOX,ROR2,SFRP1,STAT1,ZFP36,ZBTB16,TNFSF18,CD83,ISG15,MAFB,FSTL3,IL17D,NLRP3,EGR1,IL1B,ITGB6,VEGFA,CITED2,WNT4,RSAD2,SERPING1,C3,CD22,CLU,DNASE1L3,HLA-DPA1,HLA-DQA1,HLA-DQB1,IFI35,NOS2,MASP1,PTX3,RBP4,NR4A3,IL27RA,INAVA,ULBP1,CLNK,CD68,THBS1,IL1RL1
GO Biological Processes	GO:0061458	reproductive system development	4.60E-10	ALOX15B,ASCL2,PRDM1,C3,TNC,CCN1,INHA,INHBB,ROR2,SERPINB5,SERPINE2,PTGS2,PTPRN,PTX3,RBP4,SFRP1,SPP1,VEGFA,WT1,FOSL1,TNFSF10,NOG,CYP7B1,FSTL3,CITED2,TIPARP,WNT4,GREB1L
GO Biological Processes	GO:0009611	response to wounding	5.13E-10	ADORA3,ADRA2C,SERPING1,CD9,CCR1,COMP,CCN2,F2R,FN1,TNC,CCN1,INHBB,ITGB6,LOX,MAG,MMP12,SERPINE1,SERPINE2,PLAUR,SLC1A3,SPP1,THBS1,TIMP1,VEGFA,ZFP36,NOG,WNT4,SULF2,MYOZ1,SCUBE1,CLDN19,RTN4RL2,MELTF,AQP1,SFN,HK2,OAS2,OXTR,PRL,GAS1,IL1R2,PCSK6,PHEX,CORIN,PCSK1N,ASPRV1,SPOCK1,GADD45A,SERPINB5
GO Biological Processes	GO:0071357	cellular response to type I interferon	5.40E-10	IFI27,IFIT1,IRF7,MMP12,OAS1,OAS2,OAS3,STAT1,IFITM1,ISG15,USP18
GO Biological Processes	GO:0045785	positive regulation of cell adhesion	6.97E-10	EGR3,PTK2B,FN1,HLA-DPA1,HLA-DQA1,HLA-DQB1,HLA-DRA,HLA-DRB1,HLA-G,IGFBP2,CCN1,IL1B,IL15,PLAUR,SFRP1,VEGFA,ZBTB16,NR4A3,TNFSF18,CD83,CHST2,IL27RA,FSTL3,CITED2,WNT4,PDCD1LG2,CD276,NLRP3
GO Biological Processes	GO:0001503	ossification	8.21E-10	CCR1,COMP,CCN2,PTK2B,TNC,CCN1,LOX,ROR2,OMD,PHEX,PTGS2,PTHLH,RPS15,SFRP1,SPP1,WNT10B,ZBTB16,IFITM1,NOG,ISG15,MRC2,FSTL3,DKK1,CLEC5A,WNT4,CHRDL1,NOTUM,FOXO1,ITGB6
GO Biological Processes	GO:0009991	response to extracellular stimulus	9.31E-10	AOC1,AVPR1A,CD68,CNR1,CYP26A1,FOXO1,FOS,TNC,IGFBP2,IL15,INHBB,JUN,KCNB1,NR4A2,PHEX,PRL,PTGS2,SFRP1,SOD2,SPP1,STAT1,ZFP36,FOSL1,ALDH1A2,NUAK1,PCSK1N,WNT4,NUAK2,SIK1,SIK1B

GO Biological Processes	GO:0007178	transmembrane receptor protein serine/threo	1.07E-09	COMP,EGR1,FOS,CCN1,INHA,INHBB,ITGB6,JUN,LOX,ROR2,PCSK6,SFRP1,LEFTY2,THBS1,CILP,NOG,FSTL3,CITED2,DKK1,PMEPA1,SLC2A10,ATOH8,CHRDL1,HTRA4,FAM83G
GO Biological Processes	GO:0045071	negative regulation of viral genome replicat	1.26E-09	IFIT1,MX1,OAS1,OAS2,OAS3,SLPI,IFITM1,OASL,ISG15,IFIH1,RSAD2
GO Biological Processes	GO:0002237	response to molecule of bacterial origin	1.27E-09	CD68,CNR1,F2R,FMO1,FOS,GGT5,CXCL3,NR4A1,IL1B,CXCL8,JUN,MAOB,NOS2,SERPINE1,PTGS2,SLPI,SOD2,ZFP36,PTGES,LDOC1,ANKRD1,INAVA,PDCD1LG2,NLRP3,CMPK2
GO Biological Processes	GO:0032496	response to lipopolysaccharide	1.98E-09	CD68,CNR1,F2R,FMO1,FOS,GGT5,CXCL3,NR4A1,IL1B,CXCL8,JUN,MAOB,NOS2,SERPINE1,PTGS2,SLPI,SOD2,ZFP36,PTGES,LDOC1,ANKRD1,PDCD1LG2,NLRP3,CMPK2
GO Biological Processes	GO:0031214	biomineral tissue development	2.27E-09	CCR1,COMP,PTK2B,FOXO1,CCN1,ITGB6,LOX,ROR2,OMD,PHEX,PTGS2,PTHLH,SPP1,WNT10B,ISG15,WNT4,NOTUM
GO Biological Processes	GO:0019058	viral life cycle	2.43E-09	HLA-DRB1,IFI27,IFIT1,CXCL8,ITGB6,LY6E,CIITA,MX1,NCAM1,OAS1,OAS2,OAS3,PTX3,SLPI,IFITM1,OASL,ISG15,TRIM14,CXCR6,CLEC5A,IFIH1,RSAD2,LRRC15
GO Biological Processes	GO:0110148	biomineralization	2.72E-09	CCR1,COMP,PTK2B,FOXO1,CCN1,ITGB6,LOX,ROR2,OMD,PHEX,PTGS2,PTHLH,SPP1,WNT10B,ISG15,WNT4,NOTUM
GO Biological Processes	GO:0010035	response to inorganic substance	5.17E-09	AOC1,ADCY1,AQP1,AVPR1A,CD9,ENDOG,PTK2B,FOXO1,FOS,FOSB,IGFBP2,JUN,KCNH1,KRT14,MAOB,MT1X,PHEX,PTGS2,PYCR1,SOD2,SOD3,STAT1,THBS1,NR4A3,FOSL1,SPHK1,TRPA1,PTGES,FSTL3,CPNE7,SYT8
GO Biological Processes	GO:0060337	type I interferon signaling pathway	5.95E-09	IFI27,IRF7,MMP12,OAS1,OAS2,OAS3,STAT1,IFITM1,ISG15,USP18
GO Biological Processes	GO:0030282	bone mineralization	7.87E-09	CCR1,COMP,PTK2B,CCN1,LOX,ROR2,OMD,PHEX,PTGS2,PTHLH,WNT10B,ISG15,WNT4,NOTUM
GO Biological Processes	GO:0022409	positive regulation of cell-cell adhesion	8.20E-09	EGR3,HLA-DPA1,HLA-DQA1,HLA-DQB1,HLA-DRA,HLA-DRB1,HLA-G,IGFBP2,IL1B,IL15,PLAUR,ZBTB16,NR4A3,CD83,CHST2,IL27RA,FSTL3,CITED2,PDCD1LG2,CD276,NLRP3

GO Biological Processes	GO:0009615	response to virus	8.78E-09	CLU,IFI27,IFIT1,IFIT3,IL15,IRF7,ITGB6,MMP12,MX1,MX2,OAS1,OAS2,OAS3,STAT1,FOSL1,IFITM1,OASL,ISG15,IFI44,IFI44L,DDIT4,IFIH1,PARP9,RSAD2
GO Biological Processes	GO:0045069	regulation of viral genome replication	1.15E-08	IFIT1,CXCL8,MX1,OAS1,OAS2,OAS3,SLPI,IFITM1,OASL,ISG15,IFIH1,RSAD2
GO Biological Processes	GO:0001525	angiogenesis	1.44E-08	AQP1,ADGRB3,C3,CCN2,GADD45A,EGR3,PTK2B,FLT1,FN1,HK2,HLA-G,NR4A1,HSPG2,CCN1,IL1B,CXCL8,JUN,MMP19,SERPINE1,PRL,PTGS2,SFRP1,STAT1,THBS1,VEGFA,SPHK1,SEMA5A,RAMP1,STAB2,PROK1,HSPB6
GO Biological Processes	GO:0051607	defense response to virus	1.50E-08	IFI27,IFIT1,IFIT3,IL15,IRF7,MMP12,MX1,MX2,OAS1,OAS2,OAS3,STAT1,IFITM1,OASL,ISG15,IFI44L,DDIT4,IFIH1,PARP9,RSAD2
GO Biological Processes	GO:0140546	defense response to symbiont	1.50E-08	IFI27,IFIT1,IFIT3,IL15,IRF7,MMP12,MX1,MX2,OAS1,OAS2,OAS3,STAT1,IFITM1,OASL,ISG15,IFI44L,DDIT4,IFIH1,PARP9,RSAD2
GO Biological Processes	GO:0031668	cellular response to extracellular stimulus	1.84E-08	AOC1,AVPR1A,CD68,FOXO1,FOS,TNC,IL15,INHBB,JUN,KCNB1,NR4A2,PHEX,SFRP1,FOSL1,NUAK1,WNT4,NUAK2,SIK1,SIK1B
GO Biological Processes	GO:0019079	viral genome replication	2.74E-08	IFI27,IFIT1,CXCL8,MX1,OAS1,OAS2,OAS3,SLPI,IFITM1,OASL,ISG15,CXCR6,IFIH1,RSAD2
GO Biological Processes	GO:1902105	regulation of leukocyte differentiation	3.52E-08	PRDM1,CCR1,EGR3,FOS,HLA-DRA,HLA-DRB1,HLA-G,IL15,INHA,IRF7,JUN,LGALS3,ROR2,SFRP1,ZBTB16,TNFSF18,CD83,MAFB,FSTL3,NLRP3
GO Biological Processes	GO:0001655	urogenital system development	3.82E-08	ALOX15B,PRDM1,CYP26A1,EGR1,GCNT1,TNC,SERPINB5,RBP4,SFRP1,STAT1,VEGFA,WT1,ZBTB16,ALDH1A2,NOG,CRLF1,CYP7B1,FSTL3,TIPARP,WNT4,SULF2,GREB1L
GO Biological Processes	GO:0031667	response to nutrient levels	6.18E-08	AOC1,CD68,CNR1,CYP26A1,FOXO1,TNC,IGFBP2,IL15,INHBB,JUN,KCNB1,PHEX,PRL,PTGS2,SFRP1,SOD2,SPP1,STAT1,ZFP36,ALDH1A2,NUAK1,PCSK1N,WNT4,NUAK2,SIK1,SIK1B
GO Biological Processes	GO:1903039	positive regulation of leukocyte cell-cell adl	7.27E-08	EGR3,HLA-DPA1,HLA-DQA1,HLA-DQB1,HLA-DRA,HLA-DRB1,HLA-G,IGFBP2,IL1B,IL15,ZBTB16,NR4A3,CD83,CHST2,IL27RA,PDCD1LG2,CD276,NLRP3

GO Biological Processes	GO:0002521	leukocyte differentiation	7.34E-08	PRDM1,CCR1,EGR1,EGR3,PTK2B,FOS,HLA-DRA,HLA-DRB1,HLA-G,IL1B,IL15,INHA,IRF7,ITGB6,JUN,LGALS3,ROR2,SFRP1,VEGFA,ZBTB16,TNFSF18,CD83,MAFB,FSTL3,CITED2,WNT4,RSAD2,NLRP3
GO Biological Processes	GO:1901342	regulation of vasculature development	7.42E-08	AQP1,ADGRB3,C3,GADD45A,PTK2B,FLT1,HK2,HLA-G,HSPG2,IL1B,CXCL8,SERPINE1,PRL,SFRP1,STAT1,THBS1,VEGFA,SPHK1,SEMA5A,WNT4,PROK1,HSPB6
GO Biological Processes	GO:0050865	regulation of cell activation	8.78E-08	PRDM1,CD9,CD22,CNR1,CCN2,EGR3,HLA-DPA1,HLA-DQA1,HLA-DQB1,HLA-DRA,HLA-DRB1,HLA-G,IGFBP2,IL1B,IL15,INHA,LGALS3,SERPINE2,SFRP1,THBS1,ZBTB16,NR4A3,SPHK1,TNFSF18,IL1RL1,CD83,IL27RA,PDCD1LG2,CD276,NLRP3,
GO Biological Processes	GO:0007159	leukocyte cell-cell adhesion	1.85E-07	EGR3,GCNT1,HLA-DPA1,HLA-DQA1,HLA-DQB1,HLA-DRA,HLA-DRB1,HLA-G,IGFBP2,IL1B,IL15,LGALS3,ZBTB16,NR4A3,TNFSF18,CD83,CHST2,IL27RA,PODXL2,PDCD1LG2,CD276,NLRP3
GO Biological Processes	GO:0045765	regulation of angiogenesis	2.39E-07	AQP1,ADGRB3,C3,GADD45A,PTK2B,FLT1,HK2,HLA-G,HSPG2,IL1B,CXCL8,SERPINE1,PRL,SFRP1,STAT1,THBS1,VEGFA,SPHK1,SEMA5A,PROK1,HSPB6
GO Biological Processes	GO:0030278	regulation of ossification	3.86E-07	CCR1,COMP,PTK2B,CCN1,OMD,SFRP1,WNT10B,ZBTB16,ISG15,DKK1,WNT4,NOTUM
GO Biological Processes	GO:0050870	positive regulation of T cell activation	4.83E-07	EGR3,HLA-DPA1,HLA-DQA1,HLA-DQB1,HLA-DRA,HLA-DRB1,HLA-G,IGFBP2,IL1B,IL15,ZBTB16,CD83,IL27RA,PDCD1LG2,CD276,NLRP3
GO Biological Processes	GO:1903131	mononuclear cell differentiation	5.09E-07	PRDM1,EGR1,EGR3,PTK2B,HLA-DRA,HLA-DRB1,HLA-G,IL1B,IL15,INHA,IRF7,ITGB6,JUN,LGALS3,SFRP1,VEGFA,ZBTB16,TNFSF18,CD83,MAFB,WNT4,RSAD2,NLRP3
GO Biological Processes	GO:0048002	antigen processing and presentation of pept	6.24E-07	HLA-C,HLA-DPA1,HLA-DQA1,HLA-DQB1,HLA-DRA,HLA-DRB1,HLA-G,TAP1,TAP2
GO Biological Processes	GO:1903037	regulation of leukocyte cell-cell adhesion	6.29E-07	EGR3,HLA-DPA1,HLA-DQA1,HLA-DQB1,HLA-DRA,HLA-DRB1,HLA-G,IGFBP2,IL1B,IL15,LGALS3,ZBTB16,NR4A3,TNFSF18,CD83,CHST2,IL27RA,PDCD1LG2,CD276,NLRP3
GO Biological Processes	GO:0042493	response to drug	6.34E-07	ADCY1,PTK2B,FOS,FOSB,IGFBP2,IL1B,JUN,LOX,MAOB,NOS2,OXTR,PTGS2,SFRP1,SLC1A3,SLC6A11,SOD2,STAT1,THBS1,UCHL1,FOSL1,TRPA1,ANKRD1

GO Biological Processes	GO:0002694	regulation of leukocyte activation	7.02E-07	PRDM1,CD22,CNR1,EGR3,HLA-DPA1,HLA-DQA1,HLA-DQB1,HLA-DRA,HLA-DRB1,HLA-G,IGFBP2,IL1B,IL15,INHA,LGALS3,SFRP1,THBS1,ZBTB16,NR4A3,SPHK1,TNFSF18,IL1RL1,CD83,IL27RA,PDCD1LG2,CD276,NLRP3,CLNK
GO Biological Processes	GO:0010038	response to metal ion	8.16E-07	AOC1,ADCY1,AQP1,ENDOG,PTK2B,FOS,FOSB,IGFBP2,JUN,KCNH1,KRT14,MAOB,MT1X,PTGS2,SOD2,SOD3,THBS1,PTGES,FSTL3,CPNE7,SYT8
GO Biological Processes	GO:0060395	SMAD protein signal transduction	8.79E-07	FOS,INHA,INHBB,ITGB6,JUN,ROR2,LEFTY2,CILP,SLC2A10,ATOH8
GO Biological Processes	GO:0090092	regulation of transmembrane receptor prote	1.04E-06	CCN1,INHA,INHBB,LOX,PCSK6,SFRP1,LEFTY2,THBS1,CILP,NOG,FSTL3,CITED2,DKK1,PMEPA1,SLC2A10,CHRDL1,HTRA4
GO Biological Processes	GO:0019882	antigen processing and presentation	1.14E-06	CD68,HLA-C,HLA-DPA1,HLA-DQA1,HLA-DQB1,HLA-DRA,HLA-DRB1,HLA-G,TAP1,TAP2,THBS1
GO Biological Processes	GO:0042060	wound healing	1.73E-06	ADRA2C,SERPING1,CD9,COMP,F2R,FN1,TNC,CCN1,ITGB6,LOX,MMP12,SERPINE1,SERPINE2,PLAUR,THBS1,TIMP1,VEGFA,NOG,WNT4,MYOZ1,SCUBE1,CLDN19
GO Biological Processes	GO:1902107	positive regulation of leukocyte differentiati	1.91E-06	CCR1,EGR3,FOS,HLA-DRA,HLA-DRB1,HLA-G,IL15,JUN,LGALS3,ROR2,ZBTB16,CD83,NLRP3
GO Biological Processes	GO:1903708	positive regulation of hemopoiesis	1.91E-06	CCR1,EGR3,FOS,HLA-DRA,HLA-DRB1,HLA-G,IL15,JUN,LGALS3,ROR2,ZBTB16,CD83,NLRP3
GO Biological Processes	GO:0050863	regulation of T cell activation	1.96E-06	PRDM1,EGR3,HLA-DPA1,HLA-DQA1,HLA-DQB1,HLA-DRA,HLA-DRB1,HLA-G,IGFBP2,IL1B,IL15,LGALS3,ZBTB16,TNFSF18,CD83,IL27RA,PDCD1LG2,CD276,NLRP3
GO Biological Processes	GO:0031669	cellular response to nutrient levels	2.07E-06	AOC1,CD68,FOXO1,TNC,IL15,INHBB,JUN,KCNB1,PHEX,SFRP1,NUAK1,WNT4,NUAK2,SIK1,SIK1B
GO Biological Processes	GO:0072001	renal system development	2.35E-06	PRDM1,CYP26A1,EGR1,GCNT1,RBP4,SFRP1,STAT1,VEGFA,WT1,ZBTB16,ALDH1A2,NOG,CRLF1,FSTL3,TIPARP,WNT4,SULF2,GREB1L

GO Biological Processes	GO:0002252	immune effector process	3.25E-06	SERPING1,C3,CD22,CLU,DNASE1L3,PTK2B,HLA-DPA1,HLA-DQA1,HLA-DQB1,HLA-DRA,HLA-DRB1,HLA-G,IFI35,IL1B,IRF7,LGALS3,NOS2,MASP1,PTX3,RBP4,NR4A3,TNFSF18,IL27RA,INAVA,ULBP1,RSAD2,NLRP3,CLNK
GO Biological Processes	GO:0032481	positive regulation of type I interferon prod	3.93E-06	IRF7,MMP12,OAS1,OAS2,OAS3,STAT1,ISG15,IFIH1
GO Biological Processes	GO:0002399	MHC class II protein complex assembly	3.93E-06	HLA-DPA1,HLA-DQA1,HLA-DQB1,HLA-DRA,HLA-DRB1
GO Biological Processes	GO:0002503	peptide antigen assembly with MHC class I	3.93E-06	HLA-DPA1,HLA-DQA1,HLA-DQB1,HLA-DRA,HLA-DRB1
GO Biological Processes	GO:0042110	T cell activation	4.40E-06	PRDM1,EGR1,EGR3,HLA-DPA1,HLA-DQA1,HLA-DQB1,HLA-DRA,HLA-DRB1,HLA-G,IGFBP2,IL1B,IL15,LGALS3,ZBTB16,TNFSF18,CD83,IL27RA,MAFB,WNT4,PDCD1LG2,CD276,RSAD2,NLRP3
GO Biological Processes	GO:0030500	regulation of bone mineralization	4.47E-06	CCR1,COMP,PTK2B,CCN1,OMD,WNT10B,ISG15,WNT4,NOTUM
GO Biological Processes	GO:0001822	kidney development	6.39E-06	PRDM1,CYP26A1,EGR1,GCNT1,SFRP1,STAT1,VEGFA,WT1,ZBTB16,ALDH1A2,NOG,CRLF1,FSTL3,TIPARP,WNT4,SULF2,GREB1L
GO Biological Processes	GO:0002501	peptide antigen assembly with MHC protein	7.51E-06	HLA-DPA1,HLA-DQA1,HLA-DQB1,HLA-DRA,HLA-DRB1
GO Biological Processes	GO:0060700	regulation of ribonuclease activity	7.74E-06	OAS1,OAS2,OAS3,OASL
GO Biological Processes	GO:0045766	positive regulation of angiogenesis	7.97E-06	AQP1,C3,PTK2B,FLT1,HK2,IL1B,CXCL8,SERPINE1,THBS1,VEGFA,SPHK1,SEMA5A,HSPB6
GO Biological Processes	GO:1904018	positive regulation of vasculature developm	7.97E-06	AQP1,C3,PTK2B,FLT1,HK2,IL1B,CXCL8,SERPINE1,THBS1,VEGFA,SPHK1,SEMA5A,HSPB6

GO Biological Processes	GO:0002396	MHC protein complex assembly	1.01E-05	HLA-DPA1,HLA-DQA1,HLA-DQB1,HLA-DRA,HLA-DRB1
GO Biological Processes	GO:0002697	regulation of immune effector process	1.20E-05	SERPING1,C3,CD22,DNASE1L3,HLA-DRA,HLA-DRB1,HLA-G,IL1B,LGALS3,NOS2,RBP4,NR4A3,TNFSF18,IL27RA,INAVA,RSAD2,NLRP3,CLNK
GO Biological Processes	GO:0051249	regulation of lymphocyte activation	1.20E-05	PRDM1,CD22,EGR3,HLA-DPA1,HLA-DQA1,HLA-DQB1,HLA-DRA,HLA-DRB1,HLA-G,IGFBP2,IL1B,IL15,INHA,LGALS3,SFRP1,ZBTB16, TNFSF18,CD83,IL27RA,PDCD1LG2,CD276,NLRP3,CLNK
GO Biological Processes	GO:0046660	female sex differentiation	1.58E-05	INHA,INHBB,PTPRN,PTX3,RBP4,SFRP1,VEGFA,WT1,TIPARP,WNT4
GO Biological Processes	GO:0050867	positive regulation of cell activation	1.79E-05	CCN2,EGR3,HLA-DPA1,HLA-DQA1,HLA-DQB1,HLA-DRA,HLA-DRB1,HLA-G,IGFBP2,IL1B,IL15,THBS1,ZBTB16,NR4A3,IL1RL1,CD83,IL27RA,PDCD1LG2,CD276,NLRP3
GO Biological Processes	GO:0030217	T cell differentiation	1.90E-05	PRDM1,EGR1,EGR3,HLA-DRA,HLA-DRB1,HLA-G,IL1B,IL15,ZBTB16,TNFSF18,CD83,MAFB,WNT4,RSAD2,NLRP3
GO Biological Processes	GO:0071241	cellular response to inorganic substance	1.94E-05	AOC1,ADCY1,AQP1,ENDOG,FOXO1,FOS,FOSB,JUN,KCNH1,MT1X,PTGS2,FSTL3,CPNE7,SYT8
GO Biological Processes	GO:0071248	cellular response to metal ion	1.97E-05	AOC1,ADCY1,AQP1,ENDOG,FOS,FOSB,JUN,KCNH1,MT1X,PTGS2,FSTL3,CPNE7,SYT8
GO Biological Processes	GO:1903035	negative regulation of response to woundin	2.08E-05	SERPING1,CD9,SERPINE1,SERPINE2,SPP1,THBS1,WNT4,MYOZ1,CLDN19
GO Biological Processes	GO:0002504	antigen processing and presentation of pept	2.14E-05	HLA-DPA1,HLA-DQA1,HLA-DQB1,HLA-DRA,HLA-DRB1,THBS1
GO Biological Processes	GO:0008585	female gonad development	2.27E-05	INHA,INHBB,PTPRN,PTX3,SFRP1,VEGFA,WT1,TIPARP,WNT4

GO Biological Processes	GO:0046649	lymphocyte activation	2.36E-05	PRDM1,CD22,EGR1,EGR3,PTK2B,HLA-DPA1,HLA-DQA1,HLA-DQB1,HLA-DRA,HLA-DRB1,HLA-G,IGFBP2,IL1B,IL15,INHA,LGALS3,SFRP1,ZBTB16,TNFSF18,CD83,IL27RA,MAFB,WNT4,ULBP1,PDCD1LG2,CD276,RSAD2,NLRP3,CLNK
GO Biological Processes	GO:0045619	regulation of lymphocyte differentiation	2.65E-05	PRDM1,EGR3,HLA-DRA,HLA-DRB1,HLA-G,IL15,INHA,SFRP1,ZBTB16,TNFSF18,CD83,NLRP3
GO Biological Processes	GO:0070167	regulation of biomineral tissue developmen	2.68E-05	CCR1,COMP,PTK2B,CCN1,OMD,WNT10B,ISG15,WNT4,NOTUM
GO Biological Processes	GO:0030325	adrenal gland development	2.76E-05	LY6E,WT1,FSTL3,CITED2,WNT4
GO Biological Processes	GO:0002478	antigen processing and presentation of exog	2.96E-05	HLA-DPA1,HLA-DQA1,HLA-DQB1,HLA-DRA,HLA-DRB1,TAP1
GO Biological Processes	GO:0110149	regulation of biomineralization	3.16E-05	CCR1,COMP,PTK2B,CCN1,OMD,WNT10B,ISG15,WNT4,NOTUM
GO Biological Processes	GO:0046545	development of primary female sexual chara	3.42E-05	INHA,INHBB,PTPRN,PTX3,SFRP1,VEGFA,WT1,TIPARP,WNT4
GO Biological Processes	GO:0032728	positive regulation of interferon-beta produ	3.45E-05	IRF7,OAS1,OAS2,OAS3,ISG15,IFIH1
GO Biological Processes	GO:0061045	negative regulation of wound healing	3.64E-05	SERPING1,CD9,SERPINE1,SERPINE2,THBS1,WNT4,MYOZ1,CLDN19
GO Biological Processes	GO:0030098	lymphocyte differentiation	3.92E-05	PRDM1,EGR1,EGR3,PTK2B,HLA-DRA,HLA-DRB1,HLA-G,IL1B,IL15,INHA,SFRP1,ZBTB16,TNFSF18,CD83,MAFB,WNT4,RSAD2,NLRP3
GO Biological Processes	GO:0002696	positive regulation of leukocyte activation	4.03E-05	EGR3,HLA-DPA1,HLA-DQA1,HLA-DQB1,HLA-DRA,HLA-DRB1,HLA-G,IGFBP2,IL1B,IL15,THBS1,ZBTB16,NR4A3,IL1RL1,CD83,IL27RA,PDCD1LG2,CD276,NLRP3

GO Biological Processes	GO:0010755	regulation of plasminogen activation	4.18E-05	MELTF,SERPINE1,SERPINE2,THBS1
GO Biological Processes	GO:0007548	sex differentiation	4.73E-05	INHA,INHBB,ROR2,PTPRN,PTX3,RBP4,SFRP1,VEGFA,WT1,TNFSF10,FSTL3,CITED2,TIPARP,WNT4,GREB1L
GO Biological Processes	GO:0019932	second-messenger-mediated signaling	5.11E-05	ADCY1,AQP1,AVPR1A,CD22,CCR1,PTK2B,XCR1,CXCL8,NOS2,RGS2,THBS1,VEGFA,SPHK1,CXCR6,RASD1,MYOZ1
GO Biological Processes	GO:0050878	regulation of body fluid levels	5.33E-05	ADRA2C,AQP1,SERPING1,CD9,COMP,F2R,FN1,SFN,HK2,OAS2,OXTR,SERPINE1,SERPINE2,PLAUR,PRL,THBS1,VEGFA,SCUBE1
GO Biological Processes	GO:0071560	cellular response to transforming growth fa	6.95E-05	FOS,ITGB6,JUN,LOX,SFRP1,LEFTY2,THBS1,CILP,CITED2,ANKRD1,WNT4,PMEPA1,SLC2A10,HTRA4
GO Biological Processes	GO:0002468	dendritic cell antigen processing and presen	7.77E-05	CD68,HLA-DRA,HLA-DRB1,THBS1
GO Biological Processes	GO:0010757	negative regulation of plasminogen activati	7.93E-05	SERPINE1,SERPINE2,THBS1
GO Biological Processes	GO:0032609	interferon-gamma production	8.34E-05	HLA-DPA1,HLA-DRB1,IL1B,INHA,IL1RL1,IL27RA,ISG15,PDCD1LG2,CD276
GO Biological Processes	GO:0032649	regulation of interferon-gamma production	8.34E-05	HLA-DPA1,HLA-DRB1,IL1B,INHA,IL1RL1,IL27RA,ISG15,PDCD1LG2,CD276
GO Biological Processes	GO:0071559	response to transforming growth factor beta	8.91E-05	FOS,ITGB6,JUN,LOX,SFRP1,LEFTY2,THBS1,CILP,CITED2,ANKRD1,WNT4,PMEPA1,SLC2A10,HTRA4
GO Biological Processes	GO:0010955	negative regulation of protein processing	9.00E-05	GAS1,SERPINE1,SERPINE2,THBS1,IL1R2

GO Biological Processes	GO:1903318	negative regulation of protein maturation	9.00E-05	GAS1,SERPINE1,SERPINE2,THBS1,IL1R2
GO Biological Processes	GO:0002720	positive regulation of cytokine production i	9.02E-05	HLA-G,IL1B,NR4A3,INAVA,RSAD2,NLRP3,CLNK
GO Biological Processes	GO:0001649	osteoblast differentiation	9.74E-05	TNC,CCN1,LOX,PTHLH,RPS15,SFRP1,SPP1,WNT10B,IFITM1,NOG,MRC2,CLEC5A,WNT4
GO Biological Processes	GO:2000241	regulation of reproductive process	1.01E-04	ARHGDIB,CNR1,INHBB,OXTR,PAEP,SFRP1,TIMP1,VEGFA,WT1,CITED2,WNT4
GO Biological Processes	GO:0019884	antigen processing and presentation of exog	1.02E-04	HLA-DPA1,HLA-DQA1,HLA-DQB1,HLA-DRA,HLA-DRB1,TAP1
GO Biological Processes	GO:0019886	antigen processing and presentation of exog	1.07E-04	HLA-DPA1,HLA-DQA1,HLA-DQB1,HLA-DRA,HLA-DRB1
GO Biological Processes	GO:0042098	T cell proliferation	1.07E-04	HLA-DPA1,HLA-DRB1,HLA-G,IGFBP2,IL1B,IL15,LGALS3,TNFSF18,IL27RA,WNT4,PDCD1LG2,CD276
GO Biological Processes	GO:0046637	regulation of alpha-beta T cell differentiatio	1.09E-04	PRDM1,HLA-DRA,HLA-DRB1,ZBTB16,TNFSF18,CD83,NLRP3
GO Biological Processes	GO:1900046	regulation of hemostasis	1.09E-04	SERPING1,CD9,COMP,F2R,SERPINE1,SERPINE2,THBS1
GO Biological Processes	GO:0002443	leukocyte mediated immunity	1.12E-04	SERPING1,C3,CLU,DNASE1L3,HLA-DPA1,HLA-DQA1,HLA-DQB1,HLA-DRA,HLA-DRB1,HLA-G,IL1B,IRF7,NOS2,NR4A3,IL27RA,ULBP1,RSAD2,NLRP3,CLNK
GO Biological Processes	GO:0042129	regulation of T cell proliferation	1.13E-04	HLA-DPA1,HLA-DRB1,HLA-G,IGFBP2,IL1B,IL15,LGALS3,TNFSF18,IL27RA,PDCD1LG2,CD276

GO Biological Processes	GO:0090183	regulation of kidney development	1.25E-04	STAT1,VEGFA,WT1,NOG,WNT4
GO Biological Processes	GO:0045580	regulation of T cell differentiation	1.31E-04	PRDM1,EGR3,HLA-DRA,HLA-DRB1,HLA-G,IL15,ZBTB16,TNFSF18,CD83,NLRP3
GO Biological Processes	GO:0090287	regulation of cellular response to growth fa	1.33E-04	CCN1,IL1B,LOX,PCSK6,SFRP1,THBS1,NOG,FSTL3,CITED2,WNT4,SULF2,PMEPA1,SLC2A10,CHRDL1,HTRA4
GO Biological Processes	GO:1900047	negative regulation of hemostasis	1.45E-04	SERPING1,CD9,COMP,SERPINE1,SERPINE2,THBS1
GO Biological Processes	GO:0071222	cellular response to lipopolysaccharide	1.48E-04	CD68,CXCL3,IL1B,CXCL8,NOS2,SERPINE1,ZFP36,LDOC1,ANKRD1,PDCD1LG2,NLRP3,CMPK2
GO Biological Processes	GO:0032479	regulation of type I interferon production	1.49E-04	IRF7,MMP12,OAS1,OAS2,OAS3,STAT1,ISG15,IFIH1
GO Biological Processes	GO:0032606	type I interferon production	1.49E-04	IRF7,MMP12,OAS1,OAS2,OAS3,STAT1,ISG15,IFIH1
GO Biological Processes	GO:0002440	production of molecular mediator of immun	1.60E-04	CD22,HLA-DPA1,HLA-DQA1,HLA-DQB1,HLA-DRA,HLA-DRB1,HLA-G,IL1B,RBP4,NR4A3,IL27RA,INAVA,RSAD2,NLRP3,CLNK
GO Biological Processes	GO:0001823	mesonephros development	1.60E-04	SFRP1,VEGFA,WT1,ZBTB16,NOG,CRLF1,WNT4,GREB1L
GO Biological Processes	GO:0051592	response to calcium ion	1.63E-04	ADCY1,ENDOG,PTK2B,FOS,FOSB,KCNH1,THBS1,PTGES,CPNE7,SYT8
GO Biological Processes	GO:1990868	response to chemokine	1.72E-04	CCR1,PTK2B,XCR1,CXCL3,CXCL8,LOX,CCL18,CXCR6

GO Biological Processes	GO:1990869	cellular response to chemokine	1.72E-04	CCR1,PTK2B,XCR1,CXCL3,CXCL8,LOX,CCL18,CXCR6
GO Biological Processes	GO:0002819	regulation of adaptive immune response	1.94E-04	C3,HLA-DRA,HLA-DRB1,HLA-G,IL1B,IRF7,TNFSF18,IL1RL1,IL27RA,RSAD2,NLRP3
GO Biological Processes	GO:0002495	antigen processing and presentation of pept	1.98E-04	HLA-DPA1,HLA-DQA1,HLA-DQB1,HLA-DRA,HLA-DRB1
GO Biological Processes	GO:0002449	lymphocyte mediated immunity	2.00E-04	SERPING1,C3,CLU,HLA-DPA1,HLA-DQA1,HLA-DQB1,HLA-DRA,HLA-DRB1,HLA-G,IL1B,IRF7,IL27RA,ULBP1,RSAD2,NLRP3,CLNK
GO Biological Processes	GO:0009267	cellular response to starvation	2.24E-04	AOC1,FOXO1,INHBB,JUN,SFRP1,NUAK1,WNT4,NUAK2,SIK1,SIK1B
GO Biological Processes	GO:0002460	adaptive immune response based on somati	2.41E-04	SERPING1,C3,CLU,HLA-DPA1,HLA-DQA1,HLA-DQB1,HLA-DRA,HLA-DRB1,HLA-G,IL1B,IRF7,TNFSF18,IL1RL1,IL27RA,RSAD2,NLRP3
GO Biological Processes	GO:0071219	cellular response to molecule of bacterial or	2.49E-04	CD68,CXCL3,IL1B,CXCL8,NOS2,SERPINE1,ZFP36,LDOC1,ANKRD1,PDCD1LG2,NLRP3,CMPK2
GO Biological Processes	GO:0008406	gonad development	2.59E-04	INHA,INHBB,PTPRN,PTX3,SFRP1,VEGFA,WT1,TNFSF10,FSTL3,CITED2,TIPARP,WNT4
GO Biological Processes	GO:0090101	negative regulation of transmembrane recep	2.60E-04	SFRP1,CILP,NOG,FSTL3,DKK1,PMEPA1,SLC2A10,CHRDL1,HTRA4
GO Biological Processes	GO:0032608	interferon-beta production	2.72E-04	IRF7,OAS1,OAS2,OAS3,ISG15,IFIH1
GO Biological Processes	GO:0032648	regulation of interferon-beta production	2.72E-04	IRF7,OAS1,OAS2,OAS3,ISG15,IFIH1

GO Biological Processes	GO:0002711	positive regulation of T cell mediated immu	2.72E-04	HLA-DRA,HLA-DRB1,HLA-G,IL1B,RSAD2,NLRP3
GO Biological Processes	GO:0045621	positive regulation of lymphocyte differenti	2.78E-04	EGR3,HLA-DRA,HLA-DRB1,HLA-G,IL15,ZBTB16,CD83,NLRP3
GO Biological Processes	GO:0051251	positive regulation of lymphocyte activatior	2.82E-04	EGR3,HLA-DPA1,HLA-DQA1,HLA-DQB1,HLA-DRA,HLA-DRB1,HLA-G,IGFBP2,IL1B,IL15,ZBTB16,CD83,IL27RA,PDCD1LG2,CD276,NLRP3
GO Biological Processes	GO:0050670	regulation of lymphocyte proliferation	3.18E-04	CD22,HLA-DPA1,HLA-DRB1,HLA-G,IGFBP2,IL1B,IL15,LGALS3,TNFSF18,IL27RA,PDCD1LG2,CD276
GO Biological Processes	GO:0061041	regulation of wound healing	3.25E-04	SERPING1,CD9,F2R,SERPINE1,SERPINE2,THBS1,WNT4,MYOZ1,CLDN19
GO Biological Processes	GO:0045137	development of primary sexual characteristi	3.31E-04	INHA,INHBB,PTPRN,PTX3,SFRP1,VEGFA,WT1,TNFSF10,FSTL3,CITED2,TIPARP,WNT4
GO Biological Processes	GO:0016485	protein processing	3.31E-04	COMP,GAS1,MELTF,PCSK6,SERPINE1,PHEX,SERPINE2,THBS1,IL1R2,CORIN,PCSK1N,ASPRV1
GO Biological Processes	GO:0002824	positive regulation of adaptive immune resp	3.38E-04	C3,HLA-DRA,HLA-DRB1,HLA-G,IL1B,IL27RA,RSAD2,NLRP3
GO Biological Processes	GO:0002705	positive regulation of leukocyte mediated in	3.44E-04	C3,HLA-DRA,HLA-DRB1,HLA-G,IL1B,NOS2,RSAD2,NLRP3,CLNK
GO Biological Processes	GO:0032944	regulation of mononuclear cell proliferation	3.44E-04	CD22,HLA-DPA1,HLA-DRB1,HLA-G,IGFBP2,IL1B,IL15,LGALS3,TNFSF18,IL27RA,PDCD1LG2,CD276
GO Biological Processes	GO:0042594	response to starvation	3.66E-04	AOC1,FOXO1,INHBB,JUN,SFRP1,ZFP36,NUAK1,WNT4,NUAK2,SIK1,SIK1B

GO Biological Processes	GO:0002700	regulation of production of molecular medi	3.69E-04	CD22,HLA-G,IL1B,RBP4,NR4A3,IL27RA,INAVA,RSAD2,NLRP3,CLNK
GO Biological Processes	GO:0032069	regulation of nuclease activity	3.81E-04	OAS1,OAS2,OAS3,OASL
GO Biological Processes	GO:0032727	positive regulation of interferon-alpha prod	3.81E-04	IRF7,MMP12,STAT1,IFIH1
GO Biological Processes	GO:1903034	regulation of response to wounding	3.87E-04	SERPING1,CD9,F2R,SERPINE1,SERPINE2,SPP1,THBS1,WNT4,MYOZ1,CLDN19
GO Biological Processes	GO:0002822	regulation of adaptive immune response bas	4.06E-04	C3,HLA-DRA,HLA-DRB1,HLA-G,IL1B,TNFSF18,IL1RL1,IL27RA,RSAD2,NLRP3
GO Biological Processes	GO:0046632	alpha-beta T cell differentiation	4.33E-04	PRDM1,HLA-DRA,HLA-DRB1,ZBTB16,TNFSF18,CD83,RSAD2,NLRP3
GO Biological Processes	GO:0071277	cellular response to calcium ion	4.40E-04	ADCY1,ENDOG,FOS,FOSB,KCNH1,CPNE7,SYT8
GO Biological Processes	GO:0072203	cell proliferation involved in metanephros d	4.53E-04	EGR1,STAT1,WT1
GO Biological Processes	GO:2000018	regulation of male gonad development	4.53E-04	WT1,CITED2,WNT4
GO Biological Processes	GO:0002821	positive regulation of adaptive immune resp	4.59E-04	C3,HLA-DRA,HLA-DRB1,HLA-G,IL1B,IL27RA,RSAD2,NLRP3
GO Biological Processes	GO:0002699	positive regulation of immune effector proc	4.87E-04	C3,HLA-DRA,HLA-DRB1,HLA-G,IL1B,NOS2,RBP4,NR4A3,INAVA,RSAD2,NLRP3,CLNK

GO Biological Processes	GO:0002708	positive regulation of lymphocyte mediated	4.88E-04	C3,HLA-DRA,HLA-DRB1,HLA-G,IL1B,RSAD2,NLRP3,CLNK
GO Biological Processes	GO:0031639	plasminogen activation	5.39E-04	MELTF,SERPINE1,SERPINE2,THBS1
GO Biological Processes	GO:0070098	chemokine-mediated signaling pathway	5.44E-04	CCR1,PTK2B,XCR1,CXCL3,CXCL8,CCL18,CXCR6
GO Biological Processes	GO:0070613	regulation of protein processing	6.14E-04	GAS1,MELTF,SERPINE1,SERPINE2,THBS1,IL1R2
GO Biological Processes	GO:0039530	MDA-5 signaling pathway	6.16E-04	IRF7,OAS3,IFIH1
GO Biological Processes	GO:0019724	B cell mediated immunity	6.25E-04	SERPING1,C3,CLU,HLA-DPA1,HLA-DQA1,HLA-DQB1,HLA-DRA,HLA-DRB1,HLA-G,IRF7,IL27RA
GO Biological Processes	GO:0071216	cellular response to biotic stimulus	6.53E-04	CD68,CXCL3,IL1B,CXCL8,NOS2,SERPINE1,ZFP36,LDOC1,ANKRD1,PDCD1LG2,NLRP3,CMPK2
GO Biological Processes	GO:0001657	ureteric bud development	6.65E-04	SFRP1,VEGFA,WT1,NOG,CRLF1,WNT4,GREB1L
GO Biological Processes	GO:0045582	positive regulation of T cell differentiation	6.65E-04	EGR3,HLA-DRA,HLA-DRB1,HLA-G,ZBTB16,CD83,NLRP3
GO Biological Processes	GO:0030193	regulation of blood coagulation	6.66E-04	SERPING1,CD9,F2R,SERPINE1,SERPINE2,THBS1
GO Biological Processes	GO:0070663	regulation of leukocyte proliferation	6.77E-04	CD22,HLA-DPA1,HLA-DRB1,HLA-G,IGFBP2,IL1B,IL15,LGALS3,TNFSF18,IL27RA,PDCD1LG2,CD276

GO Biological Processes	GO:0002702	positive regulation of production of molecu	6.88E-04	HLA-G,IL1B,RBP4,NR4A3,INAVA,RSAD2,NLRP3,CLNK
GO Biological Processes	GO:0072163	mesonephric epithelium development	7.10E-04	SFRP1,VEGFA,WT1,NOG,CRLF1,WNT4,GREB1L
GO Biological Processes	GO:0072164	mesonephric tubule development	7.10E-04	SFRP1,VEGFA,WT1,NOG,CRLF1,WNT4,GREB1L
GO Biological Processes	GO:1903317	regulation of protein maturation	7.22E-04	GAS1,MELTF,SERPINE1,SERPINE2,THBS1,IL1R2
GO Biological Processes	GO:0070661	leukocyte proliferation	7.35E-04	CD22,CLU,HLA-DPA1,HLA-DRB1,HLA-G,IGFBP2,IL1B,IL15,LGALS3,TNFSF18,IL27RA,WNT4,PDCD1LG2,CD276
GO Biological Processes	GO:0001818	negative regulation of cytokine production	7.71E-04	FN1,HLA-DRB1,INHA,INHBB,OAS1,OAS3,THBS1,ZFP36,IL1R2,IL1RL1,CD83,IL27RA,PDCD1LG2,SLC2A10,NLRP3
GO Biological Processes	GO:0060993	kidney morphogenesis	8.08E-04	GCNT1,STAT1,VEGFA,WT1,NOG,WNT4,GREB1L
GO Biological Processes	GO:0007596	blood coagulation	8.51E-04	ADRA2C,SERPING1,CD9,COMP,F2R,FN1,SERPINE1,SERPINE2,PLAUR,THBS1,SCUBE1
GO Biological Processes	GO:0046651	lymphocyte proliferation	9.01E-04	CD22,HLA-DPA1,HLA-DRB1,HLA-G,IGFBP2,IL1B,IL15,LGALS3,TNFSF18,IL27RA,WNT4,PDCD1LG2,CD276
GO Biological Processes	GO:0002381	immunoglobulin production involved in imı	9.11E-04	HLA-DPA1,HLA-DQA1,HLA-DQB1,HLA-DRA,HLA-DRB1,IL27RA
GO Biological Processes	GO:0046631	alpha-beta T cell activation	9.33E-04	PRDM1,HLA-DRA,HLA-DRB1,IL15,ZBTB16,TNFSF18,CD83,RSAD2,NLRP3

GO Biological Processes	GO:0002718	regulation of cytokine production involved	9.73E-04	HLA-G,IL1B,NR4A3,INAVA,RSAD2,NLRP3,CLNK
GO Biological Processes	GO:0050818	regulation of coagulation	9.82E-04	SERPING1,CD9,F2R,SERPINE1,SERPINE2,THBS1
GO Biological Processes	GO:0032607	interferon-alpha production	9.88E-04	IRF7,MMP12,STAT1,IFIH1
GO Biological Processes	GO:0032647	regulation of interferon-alpha production	9.88E-04	IRF7,MMP12,STAT1,IFIH1
GO Biological Processes	GO:0032943	mononuclear cell proliferation	9.89E-04	CD22,HLA-DPA1,HLA-DRB1,HLA-G,IGFBP2,IL1B,IL15,LGALS3,TNFSF18,IL27RA,WNT4,PDCD1LG2,CD276
GO Biological Processes	GO:0007599	hemostasis	1.02E-03	ADRA2C,SERPING1,CD9,COMP,F2R,FN1,SERPINE1,SERPINE2,PLAUR,THBS1,SCUBE1
GO Biological Processes	GO:0050817	coagulation	1.02E-03	ADRA2C,SERPING1,CD9,COMP,F2R,FN1,SERPINE1,SERPINE2,PLAUR,THBS1,SCUBE1
GO Biological Processes	GO:0002367	cytokine production involved in immune res	1.03E-03	HLA-G,IL1B,NR4A3,INAVA,RSAD2,NLRP3,CLNK
GO Biological Processes	GO:1905939	regulation of gonad development	1.04E-03	WT1,CITED2,WNT4
GO Biological Processes	GO:0035270	endocrine system development	1.05E-03	CDH2,LY6E,WT1,ALDH1A2,NOG,FSTL3,CITED2,WNT4
GO Biological Processes	GO:0030195	negative regulation of blood coagulation	1.11E-03	SERPING1,CD9,SERPINE1,SERPINE2,THBS1

GO Biological Processes	GO:0045778	positive regulation of ossification	1.11E-03	CCN1,WNT10B,ZBTB16,ISG15,WNT4
GO Biological Processes	GO:0072088	nephron epithelium morphogenesis	1.14E-03	STAT1,VEGFA,WT1,NOG,WNT4,GREB1L
GO Biological Processes	GO:0002703	regulation of leukocyte mediated immunity	1.18E-03	C3,DNASE1L3,HLA-DRA,HLA-DRB1,HLA-G,IL1B,NOS2,IL27RA,RSAD2,NLRP3,CLNK
GO Biological Processes	GO:0046638	positive regulation of alpha-beta T cell diffe	1.22E-03	HLA-DRA,HLA-DRB1,ZBTB16,CD83,NLRP3
GO Biological Processes	GO:0042102	positive regulation of T cell proliferation	1.23E-03	HLA-DPA1,IGFBP2,IL1B,IL15,IL27RA,PDCD1LG2,CD276
GO Biological Processes	GO:0032760	positive regulation of tumor necrosis factor	1.31E-03	TNFRSF8,CLU,OAS1,OAS2,OAS3,THBS1,IFIH1
GO Biological Processes	GO:0043370	regulation of CD4-positive, alpha-beta T ce	1.33E-03	HLA-DRA,HLA-DRB1,TNFSF18,CD83,NLRP3
GO Biological Processes	GO:0001763	morphogenesis of a branching structure	1.38E-03	PRDM1,TNC,MYCN,SFRP1,VEGFA,WT1,NOG,CELSR1,WNT4,GREB1L
GO Biological Processes	GO:0072028	nephron morphogenesis	1.40E-03	STAT1,VEGFA,WT1,NOG,WNT4,GREB1L
GO Biological Processes	GO:0046661	male sex differentiation	1.45E-03	INHA,ROR2,SFRP1,WT1,TNFSF10,FSTL3,CITED2,WNT4,GREB1L
GO Biological Processes	GO:0046634	regulation of alpha-beta T cell activation	1.46E-03	PRDM1,HLA-DRA,HLA-DRB1,ZBTB16,TNFSF18,CD83,NLRP3

GO Biological Processes	GO:0050819	negative regulation of coagulation	1.59E-03	SERPING1,CD9,SERPINE1,SERPINE2,THBS1
GO Biological Processes	GO:0072075	metanephric mesenchyme development	1.62E-03	STAT1,WT1,WNT4
GO Biological Processes	GO:0072073	kidney epithelium development	1.63E-03	SFRP1,STAT1,VEGFA,WT1,NOG,CRLF1,WNT4,GREB1L
GO Biological Processes	GO:1903557	positive regulation of tumor necrosis factor	1.63E-03	TNFRSF8,CLU,OAS1,OAS2,OAS3,THBS1,IFIH1
GO Biological Processes	GO:0002706	regulation of lymphocyte mediated immunit	1.64E-03	C3,HLA-DRA,HLA-DRB1,HLA-G,IL1B,IL27RA,RSAD2,NLRP3,CLNK
GO Biological Processes	GO:0043372	positive regulation of CD4-positive, alpha-b	1.65E-03	HLA-DRA,HLA-DRB1,CD83,NLRP3
GO Biological Processes	GO:0007179	transforming growth factor beta receptor sig	1.66E-03	FOS,ITGB6,JUN,LOX,LEFTY2,THBS1,CITED2,PMEPA1,SLC2A10,HTRA4
GO Biological Processes	GO:2000243	positive regulation of reproductive process	1.95E-03	INHBB,OXTR,VEGFA,WT1,CITED2,WNT4
GO Biological Processes	GO:0016064	immunoglobulin mediated immune respons	1.99E-03	SERPING1,C3,CLU,HLA-DPA1,HLA-DQA1,HLA-DQB1,HLA-DRA,HLA-DRB1,IRF7,IL27RA
GO Biological Processes	GO:0001658	branching involved in ureteric bud morphog	2.03E-03	VEGFA,WT1,NOG,WNT4,GREB1L
GO Biological Processes	GO:0043367	CD4-positive, alpha-beta T cell differentiati	2.07E-03	HLA-DRA,HLA-DRB1,TNFSF18,CD83,RSAD2,NLRP3

GO Biological Processes	GO:0072006	nephron development	2.24E-03	EGR1,STAT1,VEGFA,WT1,NOG,WNT4,SULF2,GREB1L
GO Biological Processes	GO:0039528	cytoplasmic pattern recognition receptor sig	2.31E-03	IRF7,OAS3,OASL,IFIH1
GO Biological Processes	GO:0045591	positive regulation of regulatory T cell diffe	2.36E-03	HLA-DRA,HLA-DRB1,HLA-G
GO Biological Processes	GO:0090100	positive regulation of transmembrane recep	2.47E-03	CCN1,INHA,INHBB,LEFTY2,THBS1,CITED2,SLC2A10
GO Biological Processes	GO:0098586	cellular response to virus	2.49E-03	IRF7,MMP12,OAS1,OAS3,OASL,IFIH1
GO Biological Processes	GO:0002709	regulation of T cell mediated immunity	2.49E-03	HLA-DRA,HLA-DRB1,HLA-G,IL1B,RSAD2,NLRP3
GO Biological Processes	GO:0032640	tumor necrosis factor production	2.62E-03	TNFRSF8,CLU,OAS1,OAS2,OAS3,THBS1,ZFP36,IL27RA,IFIH1
GO Biological Processes	GO:0032680	regulation of tumor necrosis factor producti	2.62E-03	TNFRSF8,CLU,OAS1,OAS2,OAS3,THBS1,ZFP36,IL27RA,IFIH1
GO Biological Processes	GO:0035743	CD4-positive, alpha-beta T cell cytokine pr	2.80E-03	IL1B,RSAD2,NLRP3
GO Biological Processes	GO:0061138	morphogenesis of a branching epithelium	2.92E-03	TNC,MYCN,SFRP1,VEGFA,WT1,NOG,CELSR1,WNT4,GREB1L
GO Biological Processes	GO:0002753	cytoplasmic pattern recognition receptor sig	2.96E-03	IRF7,OAS3,OASL,INAVA,IFIH1

GO Biological Processes	GO:0060675	ureteric bud morphogenesis	3.18E-03	VEGFA,WT1,NOG,WNT4,GREB1L
GO Biological Processes	GO:0051604	protein maturation	3.21E-03	COMP,GAS1,MELTF,PCSK6,SERPINE1,PHEX,SERPINE2,THBS1,IL1R2,CORIN,PCSK1N,ASPRV1
GO Biological Processes	GO:0071706	tumor necrosis factor superfamily cytokine	3.25E-03	TNFRSF8,CLU,OAS1,OAS2,OAS3,THBS1,ZFP36,IL27RA,IFIH1
GO Biological Processes	GO:1903555	regulation of tumor necrosis factor superfar	3.25E-03	TNFRSF8,CLU,OAS1,OAS2,OAS3,THBS1,ZFP36,IL27RA,IFIH1
GO Biological Processes	GO:0048754	branching morphogenesis of an epithelial tu	3.26E-03	TNC,MYCN,VEGFA,WT1,NOG,CELSR1,WNT4,GREB1L
GO Biological Processes	GO:0072074	kidney mesenchyme development	3.29E-03	STAT1,WT1,WNT4
GO Biological Processes	GO:0072171	mesonephric tubule morphogenesis	3.41E-03	VEGFA,WT1,NOG,WNT4,GREB1L
GO Biological Processes	GO:2000242	negative regulation of reproductive process	3.41E-03	ARHGDIB,PAEP,TIMP1,WT1,WNT4
GO Biological Processes	GO:0045622	regulation of T-helper cell differentiation	3.46E-03	HLA-DRA,HLA-DRB1,TNFSF18,NLRP3
GO Biological Processes	GO:2000516	positive regulation of CD4-positive, alpha-b	3.46E-03	HLA-DRA,HLA-DRB1,CD83,NLRP3
GO Biological Processes	GO:0072080	nephron tubule development	3.50E-03	STAT1,VEGFA,WT1,NOG,WNT4,GREB1L

GO Biological Processes	GO:0072210	metanephric nephron development	3.79E-03	EGR1,STAT1,WT1,WNT4
GO Biological Processes	GO:0030501	positive regulation of bone mineralization	3.79E-03	CCN1,WNT10B,ISG15,WNT4
GO Biological Processes	GO:0060231	mesenchymal to epithelial transition	3.82E-03	STAT1,WT1,WNT4
GO Biological Processes	GO:0002250	adaptive immune response	3.84E-03	PRDM1,SERPING1,C3,CLU,PTK2B,HLA-C,HLA-DPA1,HLA-DQA1,HLA-DQB1,HLA-DRA,HLA-DRB1,HLA-G,IL1B,IRF7,TAP1,TAP2,TNFSF18, IL1RL1,IL27RA,PDCD1LG2,RSAD2,NLRP3
GO Biological Processes	GO:0061326	renal tubule development	4.11E-03	STAT1,VEGFA,WT1,NOG,WNT4,GREB1L
GO Biological Processes	GO:0007530	sex determination	4.41E-03	WT1,CITED2,WNT4
GO Biological Processes	GO:0046635	positive regulation of alpha-beta T cell activ	4.45E-03	HLA-DRA,HLA-DRB1,ZBTB16,CD83,NLRP3
GO Biological Processes	GO:2000514	regulation of CD4-positive, alpha-beta T ce	4.45E-03	HLA-DRA,HLA-DRB1,TNFSF18,CD83,NLRP3
GO Biological Processes	GO:0010812	negative regulation of cell-substrate adhesic	4.74E-03	MELTF,MMP12,SERPINE1,SPOCK1,THBS1
GO Biological Processes	GO:0072111	cell proliferation involved in kidney develo	5.04E-03	EGR1,STAT1,WT1
GO Biological Processes	GO:0002366	leukocyte activation involved in immune re	5.30E-03	DNASE1L3,PTK2B,HLA-DRA,HLA-DRB1,IFI35,LGALS3,NR4A3,TNFSF18,IL27RA,NLRP3,CLNK

GO Biological Processes	GO:0019722	calcium-mediated signaling	5.56E-03	AVPR1A,CD22,CCR1,PTK2B,XCR1,CXCL8,SPHK1,CXCR6,MYOZ1
GO Biological Processes	GO:0072078	nephron tubule morphogenesis	5.70E-03	VEGFA,WT1,NOG,WNT4,GREB1L
GO Biological Processes	GO:0030194	positive regulation of blood coagulation	5.73E-03	F2R,SERPINE1,THBS1
GO Biological Processes	GO:1900048	positive regulation of hemostasis	5.73E-03	F2R,SERPINE1,THBS1
GO Biological Processes	GO:0035710	CD4-positive, alpha-beta T cell activation	5.82E-03	HLA-DRA,HLA-DRB1,TNFSF18,CD83,RSAD2,NLRP3
GO Biological Processes	GO:0002263	cell activation involved in immune response	5.89E-03	DNASE1L3,PTK2B,HLA-DRA,HLA-DRB1,IFI35,LGALS3,NR4A3, TNFSF18,IL27RA,NLRP3,CLNK
GO Biological Processes	GO:0032729	positive regulation of interferon-gamma pro	6.04E-03	HLA-DPA1,IL1B,IL27RA,ISG15,CD276
GO Biological Processes	GO:0002726	positive regulation of T cell cytokine produ	6.47E-03	IL1B,RSAD2,NLRP3
GO Biological Processes	GO:0050820	positive regulation of coagulation	6.47E-03	F2R,SERPINE1,THBS1
GO Biological Processes	GO:0050671	positive regulation of lymphocyte proliferat	6.77E-03	HLA-DPA1,IGFBP2,IL1B,IL15,IL27RA,PDCD1LG2,CD276
GO Biological Processes	GO:0032946	positive regulation of mononuclear cell prol	7.03E-03	HLA-DPA1,IGFBP2,IL1B,IL15,IL27RA,PDCD1LG2,CD276

GO Biological Processes	GO:0061333	renal tubule morphogenesis	7.17E-03	VEGFA,WT1,NOG,WNT4,GREB1L
GO Biological Processes	GO:0072207	metanephric epithelium development	7.27E-03	STAT1,WT1,WNT4
GO Biological Processes	GO:0042730	fibrinolysis	7.27E-03	SERPING1,SERPINE1,THBS1
GO Biological Processes	GO:0042149	cellular response to glucose starvation	7.30E-03	NUAK1,NUAK2,SIK1,SIK1B
GO Biological Processes	GO:0060562	epithelial tube morphogenesis	7.48E-03	TNC,MYCN,SFRP1,VEGFA,WT1,NOG,CELSR1,MICAL2,CITED2,WNT4, GREB1L,TRIM71
GO Biological Processes	GO:0002221	pattern recognition receptor signaling pathw	7.78E-03	IFI35,IRF7,OAS1,OAS3,OASL,INAVA,IFIH1,RSAD2
GO Biological Processes	GO:0070169	positive regulation of biomineral tissue dev	7.85E-03	CCN1,WNT10B,ISG15,WNT4
GO Biological Processes	GO:0008584	male gonad development	7.88E-03	INHA,SFRP1,WT1,TNFSF10,FSTL3,CITED2,WNT4
GO Biological Processes	GO:0072273	metanephric nephron morphogenesis	8.12E-03	STAT1,WT1,WNT4
GO Biological Processes	GO:0046546	development of primary male sexual charac	8.17E-03	INHA,SFRP1,WT1,TNFSF10,FSTL3,CITED2,WNT4
GO Biological Processes	GO:0072009	nephron epithelium development	8.37E-03	STAT1,VEGFA,WT1,NOG,WNT4,GREB1L

GO Biological Processes	GO:0110151	positive regulation of biomineralization	8.43E-03	CCN1,WNT10B,ISG15,WNT4
GO Biological Processes	GO:0002456	T cell mediated immunity	9.11E-03	HLA-DRA,HLA-DRB1,HLA-G,IL1B,RSAD2,NLRP3
GO Biological Processes	GO:0003156	regulation of animal organ formation	9.99E-03	WT1,CITED2,DKK1
GO Biological Processes	GO:0045589	regulation of regulatory T cell differentiatio	9.99E-03	HLA-DRA,HLA-DRB1,HLA-G
GO Cellular Components	GO:0031012	extracellular matrix	2.42E-23	SERPINA3,APLP1,SERPING1,CDH2,CLU,COL12A1,COL17A1,COMP,CCN2,FN1,HSPG2,TNC,CCN1,ANOS1,LAMA3,LAMC2,LGALS3,LGALS3BP,LOX,LUM,MMP1,MMP12,MMP19,NCAM1,NDP,OMD,PCSK6,SERPINE1,SERPINE2,PRG2,PTX3,SFRP1,SLPI,SOD3,LEFTY2,THBS1,TIMP1,TIMP3,VEGFA,CILP,ADAMTS4,LAMB4,ABI3BP,ADAMDEC1,WNT4,CD248,LRRC15,HAPLN3,HMCN2,RTN4RL2,MXRA7,AOC1,SEMA5A,PODXL2,STAB2,SULF2,NLRP3,PTGES,PNPLA3,HLA-DRB1,KRT14,KRT17,RPS15,TUBB2A, ISG15,MATR3,HSPB6,CLDN19
GO Cellular Components	GO:0030312	external encapsulating structure	2.62E-23	SERPINA3,APLP1,SERPING1,CDH2,CLU,COL12A1,COL17A1,COMP,CCN2,FN1,HSPG2,TNC,CCN1,ANOS1,LAMA3,LAMC2,LGALS3,LGALS3BP,LOX,LUM,MMP1,MMP12,MMP19,NCAM1,NDP,OMD,PCSK6,SERPINE1,SERPINE2,PRG2,PTX3,SFRP1,SLPI,SOD3,LEFTY2,THBS1,TIMP1,TIMP3,VEGFA,CILP,ADAMTS4,LAMB4,ABI3BP,ADAMDEC1,WNT4,CD248,LRRC15,HAPLN3,HMCN2,RTN4RL2,MXRA7
GO Cellular Components	GO:0062023	collagen-containing extracellular matrix	3.01E-19	SERPINA3,APLP1,SERPING1,CDH2,CLU,COL12A1,COL17A1,COMP,CCN2,FN1,HSPG2,TNC,CCN1,LAMA3,LAMC2,LGALS3,LGALS3BP,LUM,NCAM1,NDP,OMD,PCSK6,SERPINE1,SERPINE2,PRG2,SFRP1,SLPI,SOD3,LEFTY2,THBS1,TIMP1,TIMP3,CILP,ADAMTS4,LAMB4,ABI3BP,ADAMDEC1,LRRC15,HMCN2,MXRA7

GO Cellular Components	GO:0005788	endoplasmic reticulum lumen	1.03E-11	SERPING1,C3,CDH2,CLU,COL12A1,COL17A1,CP,FMO1,FN1,TNC,IGFBP1,IGFBP4,CCN1,MELTF,PLAUR,PTGS2,QSOX1,SPP1,THBS1,TIMP1,FSTL3,WNT4,CHRDL1,ERP27,NOTUM,P4HA3
GO Cellular Components	GO:0098552	side of membrane	1.49E-11	ASTN1,CD9,CD22,CD69,CCR1,PTK2B,GEM,XCR1,HLA-C,HLA-DPA1,HLA-DQA1,HLA-DQB1,HLA-DRA,HLA-DRB1,HLA-G,IL2RB,ITGB6,NCAM1,SERPINE2,RGS1,RGS2,THBS1,IL1RL1,CRLF1,CD83,IL27RA,CXCR6,STAB2,CD248,SCUBE1,ULBP1,PDCD1LG2,CD276,ESYT3,SCARA5,GPIHBP1,RTN4RL2
GO Cellular Components	GO:0009897	external side of plasma membrane	1.01E-09	ASTN1,CD9,CD22,CD69,CCR1,XCR1,HLA-DRB1,HLA-G,IL2RB,ITGB6,NCAM1,SERPINE2,THBS1,IL1RL1,CRLF1,CD83,IL27RA,CXCR6,STAB2,CD248,SCUBE1,ULBP1,PDCD1LG2,CD276,SCARA5,GPIHBP1,RTN4RL2
GO Cellular Components	GO:0031091	platelet alpha granule	2.44E-09	SERPINA3,SERPING1,CD9,CLU,FN1,ISLR,SERPINE1,SERPINE2,QSOX1,LEFTY2,THBS1,TIMP1,VEGFA
GO Cellular Components	GO:0034774	secretory granule lumen	3.09E-09	SERPINA3,AOC1,ALDOC,SERPING1,C3,CLU,FN1,ISLR,ITIH3,LGALS3BP,SERPINE1,QSOX1,PTX3,SLPI,LEFTY2,THBS1,TIMP1,TIMP3,TTR,VEGFA,QPCT,PLAC8,BIN2
GO Cellular Components	GO:0060205	cytoplasmic vesicle lumen	3.68E-09	SERPINA3,AOC1,ALDOC,SERPING1,C3,CLU,FN1,ISLR,ITIH3,LGALS3BP,SERPINE1,QSOX1,PTX3,SLPI,LEFTY2,THBS1,TIMP1,TIMP3,TTR,VEGFA,QPCT,PLAC8,BIN2
GO Cellular Components	GO:0031983	vesicle lumen	4.14E-09	SERPINA3,AOC1,ALDOC,SERPING1,C3,CLU,FN1,ISLR,ITIH3,LGALS3BP,SERPINE1,QSOX1,PTX3,SLPI,LEFTY2,THBS1,TIMP1,TIMP3,TTR,VEGFA,QPCT,PLAC8,BIN2
GO Cellular Components	GO:0031093	platelet alpha granule lumen	9.25E-09	SERPINA3,SERPING1,CLU,FN1,ISLR,SERPINE1,QSOX1,LEFTY2,THBS1,TIMP1,VEGFA
GO Cellular Components	GO:0042611	MHC protein complex	1.32E-07	HLA-C,HLA-DPA1,HLA-DQA1,HLA-DQB1,HLA-DRA,HLA-DRB1,HLA-G
GO Cellular Components	GO:0071556	integral component of lumenal side of endo	3.01E-07	HLA-C,HLA-DPA1,HLA-DQA1,HLA-DQB1,HLA-DRA,HLA-DRB1,HLA-G

GO Cellular Components	GO:0098553	lumenal side of endoplasmic reticulum mem	3.01E-07	HLA-C,HLA-DPA1,HLA-DQA1,HLA-DQB1,HLA-DRA,HLA-DRB1,HLA-G
GO Cellular Components	GO:0098576	lumenal side of membrane	1.46E-06	HLA-C,HLA-DPA1,HLA-DQA1,HLA-DQB1,HLA-DRA,HLA-DRB1,HLA-G
GO Cellular Components	GO:0030139	endocytic vesicle	2.55E-06	AVPR1A,CD9,FLNB,GRIA3,HLA-C,HLA-DPA1,HLA-DQA1,HLA-DQB1,HLA-DRA,HLA-DRB1,HLA-G,ROR2,TAP1,TAP2,SPHK1,LPAR2,TRIM14,WNT4,STAB2
GO Cellular Components	GO:0030666	endocytic vesicle membrane	3.20E-06	CD9,GRIA3,HLA-C,HLA-DPA1,HLA-DQA1,HLA-DQB1,HLA-DRA,HLA-DRB1,HLA-G,ROR2,TAP1,TAP2,WNT4,STAB2
GO Cellular Components	GO:0042613	MHC class II protein complex	5.50E-06	HLA-DPA1,HLA-DQA1,HLA-DQB1,HLA-DRA,HLA-DRB1
GO Cellular Components	GO:0031227	intrinsic component of endoplasmic reticulu	2.11E-05	HLA-C,HLA-DPA1,HLA-DQA1,HLA-DQB1,HLA-DRA,HLA-DRB1,HLA-G,HSD11B1,TAP1,TAP2,LRRC8E,ESYT3
GO Cellular Components	GO:0012507	ER to Golgi transport vesicle membrane	6.02E-05	HLA-C,HLA-DPA1,HLA-DQA1,HLA-DQB1,HLA-DRA,HLA-DRB1,HLA-G
GO Cellular Components	GO:0030176	integral component of endoplasmic reticulu	6.58E-05	HLA-C,HLA-DPA1,HLA-DQA1,HLA-DQB1,HLA-DRA,HLA-DRB1,HLA-G,HSD11B1,TAP1,TAP2,LRRC8E
GO Cellular Components	GO:0000139	Golgi membrane	9.09E-05	RND3,GALNT3,GCNT1,HLA-C,HLA-DPA1,HLA-DQA1,HLA-DQB1,HLA-DRA,HLA-DRB1,HLA-G,NCAM1,QSOX1,ST8SIA1,CHST2,KDELR3,GALNT6,TMEM59L,ATL1,CHST7,PMEPA1,GALNT17,B3GNT7,NLRP3,GALNT13
GO Cellular Components	GO:0030669	clathrin-coated endocytic vesicle membrane	1.57E-04	CD9,HLA-DPA1,HLA-DQA1,HLA-DQB1,HLA-DRA,HLA-DRB1,ROR2
GO Cellular Components	GO:0045334	clathrin-coated endocytic vesicle	6.65E-04	CD9,HLA-DPA1,HLA-DQA1,HLA-DQB1,HLA-DRA,HLA-DRB1,ROR2

GO Cellular Components	GO:0030662	coated vesicle membrane	7.26E-04	CD9,HLA-C,HLA-DPA1,HLA-DQA1,HLA-DQB1,HLA-DRA,HLA-DRB1,HLA-G,ROR2,KDELR3
GO Cellular Components	GO:0030134	COPII-coated ER to Golgi transport vesicle	8.08E-04	HLA-C,HLA-DPA1,HLA-DQA1,HLA-DQB1,HLA-DRA,HLA-DRB1,HLA-G
GO Cellular Components	GO:0031300	intrinsic component of organelle membrane	1.28E-03	CNR1,HLA-C,HLA-DPA1,HLA-DQA1,HLA-DQB1,HLA-DRA,HLA-DRB1,HLA-G,HSD11B1,QSOX1,TAP1,TAP2,CHST2,LRRC8E,ESYT3,SUN3
GO Cellular Components	GO:0030665	clathrin-coated vesicle membrane	2.73E-03	CD9,HLA-DPA1,HLA-DQA1,HLA-DQB1,HLA-DRA,HLA-DRB1,ROR2
GO Cellular Components	GO:0005802	trans-Golgi network	3.40E-03	GCNT1,HLA-DPA1,HLA-DQA1,HLA-DQB1,HLA-DRA,HLA-DRB1,RGS20,CHST2,PCSK1N,RBFOX1,CHST7
GO Cellular Components	GO:0030135	coated vesicle	3.57E-03	ASTN1,CD9,HLA-C,HLA-DPA1,HLA-DQA1,HLA-DQB1,HLA-DRA,HLA-DRB1,HLA-G,ROR2,SNAP91,KDELR3
GO Cellular Components	GO:0098791	Golgi apparatus subcompartment	4.05E-03	GALNT3,GCNT1,HLA-DPA1,HLA-DQA1,HLA-DQB1,HLA-DRA,HLA-DRB1,RGS20,CHST2,PCSK1N,ATL1,RBFOX1,SULF2,CHST7
GO Cellular Components	GO:0031301	integral component of organelle membrane	4.23E-03	CNR1,HLA-C,HLA-DPA1,HLA-DQA1,HLA-DQB1,HLA-DRA,HLA-DRB1,HLA-G,HSD11B1,QSOX1,TAP1,TAP2,LRRC8E,SUN3
GO Cellular Components	GO:0098797	plasma membrane protein complex	4.26E-03	CDH2,CHRNE,PTK2B,GRIA3,HLA-C,HLA-DPA1,HLA-DQA1,HLA-DQB1,HLA-DRA,HLA-DRB1,HLA-G,HSPG2,IL2RB,INHA,ITGAD,ITGB6,KCNB1,KCNH1,KCNJ16,IL27RA,RAMP1
GO Cellular Components	GO:0030136	clathrin-coated vesicle	4.74E-03	ASTN1,CD9,HLA-DPA1,HLA-DQA1,HLA-DQB1,HLA-DRA,HLA-DRB1,ROR2,SNAP91
GO Cellular Components	GO:0030658	transport vesicle membrane	7.55E-03	HLA-C,HLA-DPA1,HLA-DQA1,HLA-DQB1,HLA-DRA,HLA-DRB1,HLA-G,PTPRN,SYT8

GO Molecular Functions	GO:0005539	glycosaminoglycan binding	2.12E-10	AOC1,APLP1,COMP,CCN2,FN1,CCN1,ANOS1,LAMC2,PCSK6,SERPINE2,PRG2,SFRP1,SOD3,THBS1,VEGFA,SEMA5A,PODXL2,STAB2,SULF2,NLRP3,HAPLN3
GO Molecular Functions	GO:0005201	extracellular matrix structural constituent	2.97E-09	COL12A1,COL17A1,COMP,FN1,HSPG2,TNC,CCN1,ANOS1,LAMA3,LAMC2,LUM,PRG2,THBS1,CILP,LAMB4,ABI3BP,HMCN2
GO Molecular Functions	GO:0042605	peptide antigen binding	7.05E-09	HLA-C,HLA-DPA1,HLA-DQA1,HLA-DQB1,HLA-DRA,HLA-DRB1,HLA-G,TAP1,TAP2
GO Molecular Functions	GO:0140375	immune receptor activity	1.24E-08	CCR1,FPR3,XCR1,HLA-DPA1,HLA-DQA1,HLA-DQB1,HLA-DRA,HLA-DRB1,IL2RB,IL1R2,IL1RL1,CRLF1,IL27RA,CXCR6,IL17RE
GO Molecular Functions	GO:0004866	endopeptidase inhibitor activity	3.93E-08	SERPINA3,SERPING1,C3,CST6,ITIH3,ANOS1,SERPINE1,SERPINB5,SERPINE2,SLPI,SPOCK1,TIMP1,TIMP3,PCSK1N,BIRC7,A2ML1
GO Molecular Functions	GO:0001730	2'-5'-oligoadenylate synthetase activity	6.55E-08	OAS1,OAS2,OAS3,OASL
GO Molecular Functions	GO:0030414	peptidase inhibitor activity	6.71E-08	SERPINA3,SERPING1,C3,CST6,ITIH3,ANOS1,SERPINE1,SERPINB5,SERPINE2,SLPI,SPOCK1,TIMP1,TIMP3,PCSK1N,BIRC7,A2ML1
GO Molecular Functions	GO:0008201	heparin binding	8.41E-08	AOC1,APLP1,COMP,CCN2,FN1,CCN1,ANOS1,LAMC2,PCSK6,SERPINE2,PRG2,SFRP1,SOD3,THBS1,VEGFA
GO Molecular Functions	GO:0061135	endopeptidase regulator activity	1.12E-07	SERPINA3,SERPING1,C3,CST6,ITIH3,ANOS1,SERPINE1,SERPINB5,SERPINE2,SLPI,SPOCK1,TIMP1,TIMP3,PCSK1N,BIRC7,A2ML1
GO Molecular Functions	GO:0004857	enzyme inhibitor activity	1.18E-07	SERPINA3,SERPING1,C3,CST6,SFN,GRM7,ITIH3,ANOS1,LGALS3,SERPINE1,SERPINB5,SERPINE2,RPS15,SLPI,SPOCK1,TIMP1,TIMP3,SH3BP5,PCSK1N,MYOZ1,BIRC7,PARP9,A2ML1
GO Molecular Functions	GO:0061134	peptidase regulator activity	2.19E-07	SERPINA3,SERPING1,C3,CST6,FN1,ITIH3,ANOS1,SERPINE1,SERPINB5,SERPINE2,SLPI,SPOCK1,TIMP1,TIMP3,PCSK1N,BIRC7,A2ML1

GO Molecular Functions	GO:0032395	MHC class II receptor activity	2.46E-07	HLA-DPA1,HLA-DQA1,HLA-DQB1,HLA-DRA,HLA-DRB1
GO Molecular Functions	GO:0033218	amide binding	2.84E-07	APBA2,AVPR1A,CLU,FKBP5,FOLH1,GRIA3,HLA-C,HLA-DPA1,HLA-DQA1,HLA-DQB1,HLA-DRA,HLA-DRB1,HLA-G,HSPG2,MAG, OXTR,TAP1,TAP2,PTGES,RAMP1,KDELR3,EPDR1,PNPLA3
GO Molecular Functions	GO:1901681	sulfur compound binding	3.80E-07	AOC1,APLP1,COMP,CCN2,FN1,CCN1,ANOS1,LAMC2,PCSK6,SERPINE2, PRG2,SFRP1,SOD3,THBS1,VEGFA,SEMA5A,PTGES,PNPLA3
GO Molecular Functions	GO:0004867	serine-type endopeptidase inhibitor activity	5.16E-07	SERPINA3,SERPING1,ITIH3,ANOS1,SERPINE1,SERPINB5,SERPINE2,SLPI,SPOCK1,PCSK1N,A2ML1
GO Molecular Functions	GO:0042277	peptide binding	2.05E-06	APBA2,AVPR1A,CLU,FOLH1,GRIA3,HLA-C,HLA-DPA1,HLA-DQA1,HLA-DQB1,HLA-DRA,HLA-DRB1,HLA-G,HSPG2,OXTR,TAP1,TAP2,PTGES, RAMP1,KDELR3
GO Molecular Functions	GO:0019955	cytokine binding	2.68E-06	CCR1,COMP,XCR1,IL2RB,THBS1,ZFP36,IL1R2,IL1RL1,NOG,CRLF1,IL27RA,CXCR6
GO Molecular Functions	GO:0004896	cytokine receptor activity	2.68E-05	CCR1,XCR1,IL2RB,IL1R2,IL1RL1,CRLF1,IL27RA,CXCR6,IL17RE
GO Molecular Functions	GO:0023026	MHC class II protein complex binding	6.29E-05	HLA-DPA1,HLA-DQA1,HLA-DQB1,HLA-DRA,HLA-DRB1
GO Molecular Functions	GO:0005198	structural molecule activity	8.75E-05	COL12A1,COL17A1,COMP,FN1,HLA-DRB1,HSPG2,TNC,CCN1,ANOS1, KRT14,KRT17,LAMA3,LAMC2,LUM,PRG2,RPS15,THBS1,TUBB2A,CILP, ISG15,MATR3,LAMB4,ABI3BP,HSPB6,CLDN19,HMCN2
GO Molecular Functions	GO:0023023	MHC protein complex binding	2.61E-04	HLA-DPA1,HLA-DQA1,HLA-DQB1,HLA-DRA,HLA-DRB1
GO Molecular Functions	GO:0019957	C-C chemokine binding	5.39E-04	CCR1,XCR1,ZFP36,CXCR6

GO Molecular Functions	GO:0070566	adenylyltransferase activity	1.46E-03	OAS1,OAS2,OAS3,OASL
GO Molecular Functions	GO:0019956	chemokine binding	1.85E-03	CCR1,XCR1,ZFP36,CXCR6
GO Molecular Functions	GO:0003823	antigen binding	2.08E-03	HLA-C,HLA-DPA1,HLA-DQA1,HLA-DQB1,HLA-DRA,HLA-DRB1,HLA-G,TAP1,TAP2
GO Molecular Functions	GO:0016493	C-C chemokine receptor activity	5.73E-03	CCR1,XCR1,CXCR6
GO Molecular Functions	GO:0030247	polysaccharide binding	7.27E-03	HLA-DRA,HLA-DRB1,PTX3
GO Molecular Functions	GO:0003725	double-stranded RNA binding	7.58E-03	OAS1,OAS2,OAS3,OASL,IFIH1
GO Molecular Functions	GO:0001637	G protein-coupled chemoattractant receptor	8.12E-03	CCR1,XCR1,CXCR6
GO Molecular Functions	GO:0004950	chemokine receptor activity	8.12E-03	CCR1,XCR1,CXCR6
KEGG Pathway	hsa05168	Herpes simplex infection	2.85E-12	C3,FOS,HLA-C,HLA-DPA1,HLA-DQA1,HLA-DQB1,HLA-DRA,HLA-DRB1,HLA-G,IFIT1,IL1B,IL15,IRF7,JUN,OAS1,OAS2,OAS3,STAT1,TAP1,TAP2,IFIH1,NOS2,PTGS2,FLT1,CXCL8,MMP1,VEGFA,ADCY1,EGR1,ETS2,IL2RB,WNT10B,WNT9A,ZFP36,IL1R2,FOSL1,WNT4,PTPRN,CD22,CDH2,MAG,NCAM1,PDCD1LG2,CD276,CLDN19,COMP,THBS1,TUBB2A,MRC2,POTEF,IL27RA,IL17D,LAMA3,LAMC2,CIITA,LAMB4,BIRC7,FPR3,MASP1,GADD45A,ISG15,PRG2,CD9,SPHK1
KEGG Pathway	hsa05164	Influenza A	1.72E-11	HLA-DPA1,HLA-DQA1,HLA-DQB1,HLA-DRA,HLA-DRB1,IL1B,CXCL8,IRF7,JUN,CIITA,MX1,OAS1,OAS2,OAS3,STAT1,TNFSF10,IFIH1,RSAD2,NLRP3,POTEF,FOS,IL2RB,IFIT1,CLDN19,CXCL3

KEGG Pathway	hsa05200	Pathways in cancer	1.63E-09	ADCY1,F2R,FOXO1,FN1,FOS,CXCL8,JUN,LAMA3,LAMC2,MMP1,NOS2,PTGER2,PTGER3,PTGS2,STAT1,TCF7L2,VEGFA,WNT10B,WNT9A,ZBTB16,LPAR2,ARNT2,LAMB4,WNT4,BIRC7,EGLN3,IL1B,IL1R2
KEGG Pathway	hsa05140	Leishmania infection	1.92E-09	C3,FOS,HLA-DPA1,HLA-DQA1,HLA-DQB1,HLA-DRA,HLA-DRB1,IL1B,JUN,NOS2,PTGS2,STAT1
KEGG Pathway	ko05140	Leishmaniasis	1.92E-09	C3,FOS,HLA-DPA1,HLA-DQA1,HLA-DQB1,HLA-DRA,HLA-DRB1,IL1B,JUN,NOS2,PTGS2,STAT1
KEGG Pathway	ko05323	Rheumatoid arthritis	2.12E-09	FLT1,FOS,HLA-DPA1,HLA-DQA1,HLA-DQB1,HLA-DRA,HLA-DRB1,IL1B,CXCL8,IL15,JUN,MMP1,VEGFA
KEGG Pathway	hsa05166	HTLV-I infection	7.36E-09	ADCY1,EGR1,ETS2,FOS,HLA-C,HLA-DPA1,HLA-DQA1,HLA-DQB1,HLA-DRA,HLA-DRB1,HLA-G,IL2RB,IL15,JUN,WNT10B,WNT9A,ZFP36,IL1R2,FOSL1,WNT4
KEGG Pathway	hsa04060	Cytokine-cytokine receptor interaction	1.81E-08	TNFRSF8,CCR1,FLT1,XCR1,CXCL3,IL1B,IL2RB,CXCL8,IL15,INHBB,OSM,PRL,CCL18,VEGFA,IL1R2,TNFSF10,TNFSF18,CXCR6,IL17D,IL17RE,ADCY1,PTK2B,STAT1
KEGG Pathway	hsa04940	Type I diabetes mellitus	2.27E-08	HLA-C,HLA-DPA1,HLA-DQA1,HLA-DQB1,HLA-DRA,HLA-DRB1,HLA-G,IL1B,PTPRN
KEGG Pathway	hsa04514	Cell adhesion molecules (CAMs)	9.11E-08	CD22,CDH2,HLA-C,HLA-DPA1,HLA-DQA1,HLA-DQB1,HLA-DRA,HLA-DRB1,HLA-G,MAG,NCAM1,PDCD1LG2,CD276,CLDN19
KEGG Pathway	hsa04145	Phagosome	9.86E-08	C3,COMP,HLA-C,HLA-DPA1,HLA-DQA1,HLA-DQB1,HLA-DRA,HLA-DRB1,HLA-G,TAP1,TAP2,THBS1,TUBB2A,MRC2,POTEF
KEGG Pathway	ko04659	Th17 cell differentiation	1.58E-07	FOS,HLA-DPA1,HLA-DQA1,HLA-DQB1,HLA-DRA,HLA-DRB1,IL1B,IL2RB,JUN,STAT1,IL27RA,IL17D
KEGG Pathway	hsa05332	Graft-versus-host disease	2.49E-07	HLA-C,HLA-DPA1,HLA-DQA1,HLA-DQB1,HLA-DRA,HLA-DRB1,HLA-G,IL1B

KEGG Pathway	ko05145	Toxoplasmosis	2.89E-07	HLA-DPA1,HLA-DQA1,HLA-DQB1,HLA-DRA,HLA-DRB1,LAMA3,LAMC2,CIITA,NOS2,STAT1,LAMB4,BIRC7
KEGG Pathway	ko04657	IL-17 signaling pathway	3.02E-07	FOS,FOSB,CXCL3,IL1B,CXCL8,JUN,MMP1,PTGS2,FOSL1,IL17D,IL17RE,SERPING1,C3,NOS2,NLRP3,EGR1,FOXO1,FN1,SERPINE1,STAT1,VEGFA,FLNB,POTEF,ADCY1,IRF7,SPP1,EGR3,PTK2B,HSPG2,IFIH1,IL15
KEGG Pathway	hsa04612	Antigen processing and presentation	4.32E-07	HLA-C,HLA-DPA1,HLA-DQA1,HLA-DQB1,HLA-DRA,HLA-DRB1,HLA-G,CIITA,TAP1,TAP2
KEGG Pathway	ko04080	Neuroactive ligand-receptor interaction	6.50E-07	ADORA3,ADRA2C,AVPR1A,CHRNE,CNR1,F2R,FPR3,GABRA4,GABRP,GRIA3,GRIK1,GRM7,OXTR,PRL,PTGER2,PTGER3,LPAR2,RXFP1
KEGG Pathway	ko04512	ECM-receptor interaction	7.84E-07	COMP,FN1,HSPG2,TNC,ITGB6,LAMA3,LAMC2,SPP1,THBS1,LAMB4
KEGG Pathway	hsa05330	Allograft rejection	2.15E-06	HLA-C,HLA-DPA1,HLA-DQA1,HLA-DQB1,HLA-DRA,HLA-DRB1,HLA-G
KEGG Pathway	ko05150	Staphylococcus aureus infection	3.00E-06	C3,FPR3,HLA-DPA1,HLA-DQA1,HLA-DQB1,HLA-DRA,HLA-DRB1,MASP1
KEGG Pathway	hsa05169	Epstein-Barr virus infection	7.97E-06	GADD45A,HLA-C,HLA-DPA1,HLA-DQA1,HLA-DQB1,HLA-DRA,HLA-DRB1,HLA-G,IRF7,JUN,OAS1,OAS2,OAS3,STAT1,TAP1,TAP2,ISG15
KEGG Pathway	hsa05310	Asthma	8.67E-06	HLA-DPA1,HLA-DQA1,HLA-DQB1,HLA-DRA,HLA-DRB1,PRG2
KEGG Pathway	ko05321	Inflammatory bowel disease (IBD)	9.39E-06	HLA-DPA1,HLA-DQA1,HLA-DQB1,HLA-DRA,HLA-DRB1,IL1B,JUN,STAT1
KEGG Pathway	hsa05162	Measles	1.01E-05	FOS,IL1B,IL2RB,IRF7,JUN,MX1,OAS1,OAS2,OAS3,STAT1,TNFSF10,IFIH1

KEGG Pathway	ko04151	PI3K-Akt signaling pathway	1.24E-05	COMP,F2R,FLT1,FN1,NR4A1,TNC,IL2RB,ITGB6,LAMA3,LAMC2,OSM,PRL,SPP1,THBS1,VEGFA,LPAR2,LAMB4,DDIT4
KEGG Pathway	ko04658	Th1 and Th2 cell differentiation	1.75E-05	FOS,HLA-DPA1,HLA-DQA1,HLA-DQB1,HLA-DRA,HLA-DRB1,IL2RB,JUN,STAT1
KEGG Pathway	hsa05320	Autoimmune thyroid disease	2.14E-05	HLA-C,HLA-DPA1,HLA-DQA1,HLA-DQB1,HLA-DRA,HLA-DRB1,HLA-G
KEGG Pathway	ko04510	Focal adhesion	2.19E-05	COMP,FLNB,FLT1,FN1,TNC,ITGB6,JUN,LAMA3,LAMC2,SPP1,THBS1,VEGFA,LAMB4
KEGG Pathway	ko05146	Amoebiasis	2.47E-05	ADCY1,FN1,IL1B,CXCL8,LAMA3,LAMC2,NOS2,IL1R2,LAMB4
KEGG Pathway	hsa04640	Hematopoietic cell lineage	2.68E-05	CD9,CD22,HLA-DPA1,HLA-DQA1,HLA-DQB1,HLA-DRA,HLA-DRB1,IL1B,IL1R2
KEGG Pathway	ko05133	Pertussis	3.01E-05	SERPING1,C3,FOS,IL1B,CXCL8,JUN,NOS2,NLRP3
KEGG Pathway	ko04933	AGE-RAGE signaling pathway in diabetic c	3.16E-05	EGR1,FOXO1,FN1,IL1B,CXCL8,JUN,SERPINE1,STAT1,VEGFA
KEGG Pathway	ko05152	Tuberculosis	3.51E-05	C3,HLA-DPA1,HLA-DQA1,HLA-DQB1,HLA-DRA,HLA-DRB1,IL1B,CIITA,NOS2,STAT1,SPHK1,MRC2
KEGG Pathway	hsa05416	Viral myocarditis	4.35E-05	HLA-C,HLA-DPA1,HLA-DQA1,HLA-DQB1,HLA-DRA,HLA-DRB1,HLA-G
KEGG Pathway	hsa05205	Proteoglycans in cancer	5.66E-05	FLNB,FN1,HSPG2,LUM,PLAUR,THBS1,TIMP3,VEGFA,WNT10B,WNT9A,WNT4,SMOX,POTEF

KEGG Pathway	M00056	O-glycan biosynthesis, mucin type core	6.29E-05	GALNT3,GCNT1,GALNT6,GALNT17,GALNT13
KEGG Pathway	hsa05202	Transcriptional misregulation in cancer	1.23E-04	GADD45A,FOXO1,FLT1,GRIA3,IL2RB,CXCL8,MYCN,WT1,ZBTB16,IL1R2,NR4A3,ARNT2
KEGG Pathway	ko00512	Mucin type O-glycan biosynthesis	1.25E-04	GALNT3,GCNT1,GALNT6,GALNT17,GALNT13
KEGG Pathway	hsa00512	O-Glycan biosynthesis	1.25E-04	GALNT3,GCNT1,GALNT6,GALNT17,GALNT13
KEGG Pathway	hsa04672	Intestinal immune network for IgA producti	1.29E-04	HLA-DPA1,HLA-DQA1,HLA-DQB1,HLA-DRA,HLA-DRB1,IL15
KEGG Pathway	ko00330	Arginine and proline metabolism	1.45E-04	AOC1,MAOB,NOS2,PYCR1,SMOX,P4HA3
KEGG Pathway	hsa05132	Salmonella infection	1.49E-04	FLNB,FOS,CXCL3,IL1B,CXCL8,JUN,NOS2,POTEF
KEGG Pathway	ko05142	Chagas disease (American trypanosomiasis	2.43E-04	ADCY1,C3,FOS,IL1B,CXCL8,JUN,NOS2,SERPINE1
KEGG Pathway	ko04610	Complement and coagulation cascades	2.81E-04	SERPING1,C3,CLU,F2R,SERPINE1,PLAUR,MASP1
KEGG Pathway	hsa05222	Small cell lung cancer	4.10E-04	FN1,LAMA3,LAMC2,NOS2,PTGS2,LAMB4,BIRC7
KEGG Pathway	hsa05160	Hepatitis C	4.25E-04	IFIT1,CXCL8,IRF7,MX1,OAS1,OAS2,OAS3,STAT1,RSAD2,CLDN19

KEGG Pathway	hsa04621	NOD-like receptor signaling pathway	4.46E-04	CXCL3,IL1B,CXCL8,IRF7,JUN,OAS1,OAS2,OAS3,STAT1,NLRP3
KEGG Pathway	ko04310	Wnt signaling pathway	5.24E-04	JUN,SFRP1,TCF7L2,WNT10B,WNT9A,FOSL1,DKK1,WNT4,NOTUM
KEGG Pathway	ko05031	Amphetamine addiction	7.82E-04	FOS,FOSB,GRIA3,JUN,MAOB,ARC,ADCY1,F2R,OXTR,PTGER2,PTGER3,HCAR3,HCAR2,CLU,PTGS2,RGS2,POTEF
KEGG Pathway	hsa04072	Phospholipase D signaling pathway	1.39E-03	ADCY1,AVPR1A,F2R,PTK2B,GRM7,CXCL8,SPHK1,LPAR2,NLRP3,NOS2,OXTR,PTGER3
KEGG Pathway	ko04024	cAMP signaling pathway	1.43E-03	ADCY1,F2R,FOS,GRIA3,JUN,OXTR,PTGER2,PTGER3,HCAR3,HCAR2
KEGG Pathway	ko04620	Toll-like receptor signaling pathway	1.46E-03	FOS,IL1B,CXCL8,IRF7,JUN,SPP1,STAT1
KEGG Pathway	hsa05161	Hepatitis B	2.34E-03	EGR3,PTK2B,FOS,HSPG2,CXCL8,IRF7,JUN,STAT1,IFIH1
KEGG Pathway	hsa04062	Chemokine signaling pathway	2.82E-03	ADCY1,CCR1,PTK2B,XCR1,CXCL3,CXCL8,CCL18,STAT1,CXCR6
KEGG Pathway	hsa04066	HIF-1 signaling pathway	3.14E-03	FLT1,HK2,NOS2,SERPINE1,TIMP1,VEGFA,EGLN3
KEGG Pathway	ko05219	Bladder cancer	4.15E-03	CXCL8,MMP1,THBS1,VEGFA
KEGG Pathway	hsa04921	Oxytocin signaling pathway	4.61E-03	ADCY1,CLU,FOS,JUN,OXTR,PTGS2,RGS2,POTEF

KEGG Pathway	ko04115	p53 signaling pathway	5.05E-03	GADD45A,SFN,SERPINE1,SERPINB5,THBS1
KEGG Pathway	ko04723	Retrograde endocannabinoid signaling	5.82E-03	ADCY1,CNR1,GABRA4,GABRP,GRIA3,PTGS2
KEGG Pathway	M00001	Glycolysis (Embden-Meyerhof pathway), gl	7.27E-03	ALDOC,HK2,PFKP
KEGG Pathway	ko05144	Malaria	7.85E-03	COMP,IL1B,CXCL8,THBS1
KEGG Pathway	ko04668	TNF signaling pathway	8.01E-03	FOS,CXCL3,IL1B,IL15,JUN,PTGS2
KEGG Pathway	ko04020	Calcium signaling pathway	9.42E-03	ADCY1,AVPR1A,F2R,PTK2B,NOS2,OXTR,PTGER3,SPHK1

TABLE S7. Correlation between SLE-dysregulated genes and SLEDAI scores.

**Gene Name**	r^2	***P***
APOLD1	0.0800	0.50
C3	0.0623	0.55
CTGF	0.0106	0.81
DUSP1	0.2611	0.20
FN1	0.2820	0.18
GH2	0.0159	0.77
MMP1	0.0518	0.59
PLAC1	0.4766	0.06
PSG4	0.0107	0.81
VEGFA	0.0004	0.96
